# Identification of shared single copy nuclear genes in *Arabidopsis, Populus, Vitis *and *Oryza *and their phylogenetic utility across various taxonomic levels

**DOI:** 10.1186/1471-2148-10-61

**Published:** 2010-02-24

**Authors:** Jill M Duarte, P Kerr Wall, Patrick P Edger, Lena L Landherr, Hong Ma, J Chris Pires, Jim Leebens-Mack, Claude W dePamphilis

**Affiliations:** 1 Department of Biology and the Huck Institutes of the Life Sciences, The Pennsylvania State University, University Park, PA, 16802, USA; 2 Division of Biological Sciences, University of Missouri-Columbia, Columbia, MO 65211-7310, USA; 3 Department of Plant Biology, University of Georgia, Athens, GA 30602-7271, USA; 4 State Key Laboratory of Genetic Engineering, the Institute of Plant Biology, School of Life Sciences, Institutes of Biomedical Sciences, Center for Evolutionary Biology, Fudan University, 220 Handan Road, Shanghai 200433, PR China; 5 BASF Plant Science, Research Triangle Park, NC, 27709, USA

## Abstract

**Background:**

Although the overwhelming majority of genes found in angiosperms are members of gene families, and both gene- and genome-duplication are pervasive forces in plant genomes, some genes are sufficiently distinct from all other genes in a genome that they can be operationally defined as 'single copy'. Using the gene clustering algorithm MCL-tribe, we have identified a set of 959 single copy genes that are shared single copy genes in the genomes of *Arabidopsis thaliana, Populus trichocarpa, Vitis vinifera *and *Oryza sativa*. To characterize these genes, we have performed a number of analyses examining GO annotations, coding sequence length, number of exons, number of domains, presence in distant lineages, such as *Selaginella *and *Physcomitrella*, and phylogenetic analysis to estimate copy number in other seed plants and to demonstrate their phylogenetic utility. We then provide examples of how these genes may be used in phylogenetic analyses to reconstruct organismal history, both by using extant coverage in EST databases for seed plants and *de novo *amplification via RT-PCR in the family Brassicaceae.

**Results:**

There are 959 single copy nuclear genes shared in *Arabidopsis*, *Populus*, *Vitis *and *Oryza *["APVO SSC genes"]. The majority of these genes are also present in the *Selaginella *and *Physcomitrella *genomes. Public EST sets for 197 species suggest that most of these genes are present across a diverse collection of seed plants, and appear to exist as single or very low copy genes, though exceptions are seen in recently polyploid taxa and in lineages where there is significant evidence for a shared large-scale duplication event. Genes encoding proteins localized in organelles are more commonly single copy than expected by chance, but the evolutionary forces responsible for this bias are unknown.

Regardless of the evolutionary mechanisms responsible for the large number of shared single copy genes in diverse flowering plant lineages, these genes are valuable for phylogenetic and comparative analyses. Eighteen of the APVO SSC single copy genes were amplified in the Brassicaceae using RT-PCR and directly sequenced. Alignments of these sequences provide improved resolution of Brassicaceae phylogeny compared to recent studies using plastid and ITS sequences. An analysis of sequences from 13 APVO SSC genes from 69 species of seed plants, derived mainly from public EST databases, yielded a phylogeny that was largely congruent with prior hypotheses based on multiple plastid sequences. Whereas single gene phylogenies that rely on EST sequences have limited bootstrap support as the result of limited sequence information, concatenated alignments result in phylogenetic trees with strong bootstrap support for already established relationships. Overall, these single copy nuclear genes are promising markers for phylogenetics, and contain a greater proportion of phylogenetically-informative sites than commonly used protein-coding sequences from the plastid or mitochondrial genomes.

**Conclusions:**

Putatively orthologous, shared single copy nuclear genes provide a vast source of new evidence for plant phylogenetics, genome mapping, and other applications, as well as a substantial class of genes for which functional characterization is needed. Preliminary evidence indicates that many of the shared single copy nuclear genes identified in this study may be well suited as markers for addressing phylogenetic hypotheses at a variety of taxonomic levels.

## Background

### Gene duplication as a pervasive force in the molecular evolution of angiosperms

As a result of pervasive and recurring small-scale duplications (e.g. local, tandem, segmental) and whole genome duplications [1-3], which may be followed by functional divergence, many nuclear genes in angiosperms are members of gene families and may exhibit copy number variation. This complicates the identification of potentially orthologous nuclear genes that could be used for applications such as molecular systematics and mapping markers. However, there is a small subset of genes that appear to persist in low copy numbers, ranging from 1-4 copies per taxa [4-6]. In the extreme case are genes that have resolved to single copy within a few million years after duplication in many independent lineages. There is evidence from *Arabidopsis *that genes that become single copy following genome duplication are more likely to return to single copy status after subsequent genome duplications [7]. This suggests that there could be a small subset of single copy nuclear genes that are single copy throughout much of angiosperm diversity.

However, dosage-insensitive products are also likely at random to repeatedly return to single copy [8]. If a small subset (< 15) of sequenced genomes is sampled for shared single-copy genes, it is likely that a subset of these genes would be merely shared by chance in single copy by all the surveyed genomes. As would be predicted, dosage-insensitive gene categories more frequently exhibit copy number variation compared to dosage-sensitive genes [9]. Thus, dosage-insensitive genes may be shared by chance in single copy when comparing a few plant genomes, but in general would vary greatly in copy number throughout the plant kingdom. As the number of surveyed genomes increases, we predict that the number of dosage-insensitive genes in the shared single copy list will steadily decrease. Genes that encode dosage-insensitive products should ideally not be used as phylogenetic markers. Dosage-insensitive genes, shared in single-copy, may be paralogs that have randomly lost alternate copies since the shared duplication, which are known to produce artifacts in phylogenetic reconstruction, including inaccurate reconstructions of organismal history [10]. However, other genes, which we would term conserved single copy genes, that are truly shared in single copy (i.e. strict orthologs) throughout seed plants are ideal nuclear phylogenetic markers. Conserved single copy genes have previously never been characterized in a large scale, and possible common mechanisms that repeatedly and convergently return these genes to single copy are still unknown.

### An ancient history of genome duplication in plant genomes

Whole genome duplications have been inferred in all angiosperm genomes sequenced to date. Analysis of the *Arabidopsis thaliana *genome provides evidence based on synteny for at least three whole genome duplications [1,11-14]. Analysis of the *Oryza sativa *genome suggests two genome duplications in the evolutionary history of the genome, one close to the divergence of the Poaceae and another older duplication [15,16]. The *Populus trichocarpa *genome shows evidence for three whole genome duplications [17]. The *Vitis vinifera *genome is currently interpreted to show evidence of an ancient hexaploidy event with no recent whole genome duplications [18]. These results, in addition to evidence from analysis of ESTs in a number of species throughout the angiosperm tree of life, suggests that polyploidy has occurred in most if not all major extant angiosperm lineages [1-3]. Evidence for frequent gene duplication is also seen in the evolutionary history of numerous gene families that have expanded during the diversification of the angiosperms [19-22]. Gene families that retain duplicated genes can provide rich evidence about species relationships. If the rate of gene duplication and loss is modest relative to the rate of speciation, gene duplications should result in duplicate gene trees that are reciprocally monophyletic. Additionally, the position of a gene duplication in a gene tree can itself be valuable phylogenetic information. But obtaining sequences from throughout multiple gene families for large numbers of target species is costly as well as being experimentally and analytically challenging. The prevalence of duplication in flowering plants means that orthologous loci without retained duplicates in one or more flowering plant lineages may be rare. Especially for phylogenetics, it has been considered important to identify orthologous sequences. Evidence from yeast indicates that phylogenies based on paralogous genes with asymmetric divergence are misrepresentations of the organismal phylogeny [10]. Duplication has been well documented to result in rate asymmetries in paralogs [10,23,24]. This rate variation that can confound phylogenetic analysis by introducing long branch attraction artifacts [10,25]. Although strict orthologs cannot be identified without intensive study and identification of locus position, co-orthologs that are single or low-copy may reduce artifacts introduced by asymmetric divergence after duplication [10] and can be easier to amplify and sequence for phylogenetic studies than genes that are members of gene families.

### Previously identified single copy nuclear genes in flowering plants

Relatively few single copy nuclear genes (in the context of the entire genome) have been well studied in flowering plants. Shared single copy nuclear genes in flowering plants are in the unique position of being the closest semblance of strict orthologs in their genomes, and therefore are of great interest. Given the amount of duplication present in flowering plant genomes and their evolutionary history, orthologous sequences that are only separated by speciation events and have not been duplicated since the most recent common ancestor can be considered to be rare, and the number of genes that can be considered orthologous decreases dramatically as we compare increasingly distant lineages. The identification of orthologous sequences is especially relevant for molecular systematics, since the addition of easily amplified and phylogenetically informative sequences to current datasets allows for the independent testing of phylogenetic hypotheses using as much data as possible. A recent study using high-throughput techniques to identify orthologous sequences in animals showed improved resolution of the animal tree of life when putatively single copy nuclear genes were used to determine the phylogeny and was able to test a variety of different phylogenetic hypotheses [26].

There has been a significant amount of attention paid towards the prospect of identifying single copy nuclear genes in flowering plants, primarily for their potential use as phylogenetic markers [4-6,27-32]. A number of low copy nuclear genes have been previously identified in flowering plants, including the phytochromes, *ADH, TPI, GAP3DH, LEAFY, ACCase, PGK, petD, GBSSI, GPAT, ncpGS, GIGANTEA, GPA1, AGB1, PPR *and *RBP2*, primarily for their use as phylogenetic markers [5,32-46]. Evidence from wheat indicates that duplicated low-copy genomic regions, which may include low-copy genes similar to those in the present study, are rapidly eliminated following polyploidization [47]. In the rare instances in which duplicated copies of single or low copy genes are maintained over long evolutionary periods (tens of millions of years), paralogs show distinct patterns of functional and/or expression divergence. For example, over expression of LEAFY generally results in early flowering [48,49] and cases in which LEAFY is present in duplicate (typically in recent polyploids), expression patterns are typically complementary, suggesting that subfunctionalization may be necessary for the maintenance of both loci [50,51].

### Current tools for phylogenetic analysis in angiosperms

Molecular systematics in flowering plants has been dominated by the use of phylogenetic markers derived from the plastid genome (e.g., *rbc*L, *mat*K. *ndh*F, *trn*L-F) or ribosomal DNA (18S, 26S, ITS, ETS). The predominant use of plastid and ribosomal DNA markers limits the number of genes available phylogenetic analysis. Typically only angiosperms with sequenced genomes are included in taxon sets for large eukaryotic tree of life datasets [52,53]. Although the majority of phylogenetic markers used in angiosperms are from the plastid or mitochondrial genomes, low copy nuclear genes have been sought after as phylogenetic and mapping markers [4,29,30,54]. The incorporation of nuclear markers to a combined dataset including plastid and ribosomal DNA markers should improve the robustness of phylogenetic reconstructions at all taxonomic levels by increasing the total number of informative characters (i.e. increasing phylogenetic signal) [30]. For example, a combined analysis of multiple low-copy nuclear genes did provide an improved and robust phylogeny that failed to fully resolve using cpDNA and nrDNA sequence data [55]. Further, nuclear genes are necessary to detect hybridization, introgression events, and ancient allopolyploidization events [30,56]. Ribosomal DNA markers, which undergo concerted evolution, are unreliable for reconstructing ancient allopolyploidization events [30]. However, both recent and ancient gene duplications have complicated the identification of low-copy genes. Nonetheless, the nuclear genome is an important source of genetic diversity that can be used to establish phylogenetic relationships between species, genera, families, and deeper lineages, and resolve the timing of landmark events such as the origin of angiosperms [57,58] and origin of eukaryotes[52,59,60]. For instance, previous studies indicate that intron sequences from nuclear genes such as *LFY*, *ACCase*, *PGK*, *petD*, *GBSSI*, *GPAT*, *ncpGS *and others are at least as useful as ITS or plastid intron/spacer sequences in resolving family-level phylogenies and in many cases are more informative than ITS and plastid intron or spacer sequences [34,42,43]. However, because of lineage-specific duplications, datasets using protein-coding nuclear genes to resolve relationships across all angiosperms are limited (but see *PHYC *[57,58]; Pires, unpublished data) and analyses of organellar and ribosomal markers are more typical. Recently, whole plastid genome sequencing, has been used to decipher the angiosperm tree of life [61,62].

Studies using genes that are members of gene families are excellent for identifying duplication events that can be used as synapomorphies and using the greatest amount of data available, since the majority of genes in the nuclear genome of flowering plants are members of gene families [2,18,63-67]. However, there are several technical limitations concerning the use of genes that are members of larger gene families. In large informatic-based studies, it can be difficult to eliminate noise, identify closest co-orthologs and select appropriate models of sequence evolution. In traditional PCR-based amplification of phylogenetic markers, the co-occurrence of paralogs results in increased efforts to isolate and sequence all available members of the gene family, with increased time spent in amplification and cloning to separate sequences from different members of the same gene family. However, it is possible that members of gene families can be used for phylogenetic purposes if copy number is stable and orthology can be easily assessed[32].

In this paper, we describe analyses of a set of shared single copy nuclear genes identified in four sequenced angiosperm genomes. The first analysis utilizes EST contigs and sequences from the TIGR Plant Transcript Assemblies to investigate the utility of 18 shared single copy nuclear genes for deep phylogenetic analysis, as well as to identify the occurrence and timing of lineage-specific duplications. This application of the shared single copy genes will provide information about whether these genes are suitable for use as phylogenetic markers and whether these genes have the ability to provide phylogenetically informative sequences. The second analysis is a family-level phylogeny in the Brassicaceae based on sequences for a set of shared single copy genes that have been amplified by RT-PCR and sequenced. The mustard family (Brassicaceae, 338 genera, 3,700 species) is an ideal system to test the utility of these shared single-copy nuclear genes for phylogenetic studies and to test if these genes have repeatedly returned to a single copy state following multiple whole genome duplication and diploidization events. The *Arabidopsis thaliana *genome harbors signatures of at least three rounds of whole genome duplication [1,11-14], and the "diploid" *Brassica *species have undergone additional duplication events [68,69]. A paleopolyploid event occurred approximately 40 mya near the origin of the family (the alpha event in *Arabidopsis*, [70]), while the *Arabidopsis *beta event occurred within the order Brassicales following the divergence from papaya [8,71], and a more recent putative triplication event is shared within the tribe Brassiceae that occurred 7.9-14.6 mya [68-70].

### Summary

In the face of local, tandem, segmental, and whole genome duplications, non-random gene loss may maintain some genes in low copy number [7,65,72]. The number of single copy genes shared among the four genomes investigated here is higher than expected if one assumes that all duplicate gene pairs are equally likely to lose one copy following genome-wide duplication events [72]. This observation could be explained by selection for the retention of dosage-sensitive duplicates to maintain dosage balance following genome duplication in which case the shared single copy genes may represent a random subset of genes that are free to be lost following duplication. Alternatively, there may be selection to conserve some genes as singletons in plant genomes. Of course, these are not mutually exclusive hypotheses. In either case, we predict that RT-PCR amplification of multiple single copy nuclear genes will yield many single copy genes with sufficient phylogenetic information to resolve family-level phylogenies. At the same time, researchers must remain aware of the impact that paralogous genes may have on phylogenetic inference.

## Results

### Shared single copy genes are numerous in angiosperm genomes, as well as in other plant lineages

**Figure 1:**
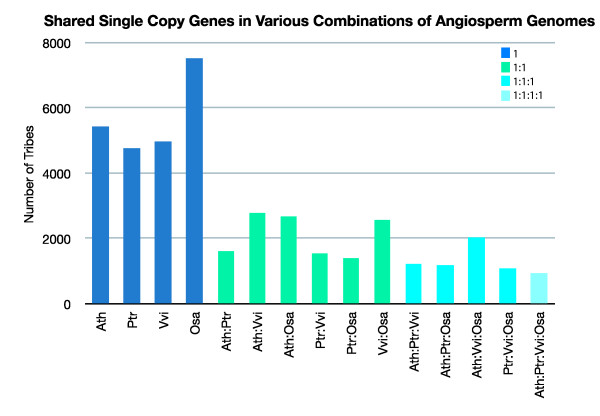
**Shared single copy genes in various combinations of angiosperm genomes**. Angiosperm genomes are abbreviated as follows: Ath - *Arabidopsis thaliana*; Ptr - *Populus trichocarpa*; Vvi - *Vitis vinifera*; Osa - *Orysa sativa*. The number of tribes represents the number of PlantTribes found at medium stringency (3.0) that contain a single member from each of the genomes sampled.

We first determined the number of single copy genes in each of the four species with whole genome sequences: *Arabidopsis*, *Populus*, *Vitis *and *Oryza*, and found 4762 to 7542 genes (Figure 1 and Additional File 1). The numbers of single copy genes shared between any two of the four species were much smaller, from 1424 to 2796, particularly when *Populus *was included. Single copy genes shared in any three species or all four species decreased further, but only slightly (Figure 1). This suggests that the entire shared single copy genes list is not composed of a random set of genes, with 959 genes shared between *Arabidopsis*, *Populus*, *Vitis *and *Oryza *(APVO) as defined by the number of PlantTribes [63] at stringency 3.0 that contain a single member from each respective genome (Figure 1 and Additional File 1). Examination of the TIGR Plant Transcript assemblies [73] indicates that these genes are also present throughout angiosperms, although exact copy number cannot be ascertained since EST assemblies are not an accurate estimate of copy number. For example, the average number of plant transcript assemblies per single copy PlantTribe for *Arabidopsis *and *Oryza *are 1.22 and 1.51, respectively (Additional file 2). Assemblies for taxa with hundreds of thousands of ESTs very likely identify some allelic and splice-site variants (vs. true duplicates) as distinct transcripts.

We characterized duplication events in gene phylogenies for eighteen shared single copy genes identified in the sequenced genomes and with particularly good overlap in sequence availability through the major angiosperm groups. We found that duplicate copies (paralogs and co-orthologs) were restricted to recent polyploidy lineages. Lineage-specific paralogous and co-orthologous pairs represented independent duplications in the Asteraceae, Solanaceae, Fabaceae, and *Gossypium *(Additional file 3). Comparison of the set of shared single copy genes identified in this study to a set of single copy and low-copy nuclear genes that have been commonly used for angiosperm phylogenetics [5,37-44,57,58] indicates that few previously-utilized phylogenetic markers (such as *LFY*) are members of APVO shared single copy PlantTribes - i.e. the set of previously used phylogenetic markers typically belong to PlantTribes in which copy number varies among the four genomes examined and/or these phylogenetic markers are members of larger gene families.

When we consider the four angiosperm genomes along with those of two additional land plants, *Selaginella *(a lycophyte, or early divergent vascular plant), *Physcomitrella *(a moss, or non-vascular plant), there are 395 PlantTribes that include a single gene from each of the six genomes. When copy number in *Selaginella *or *Physcomitrella *is not restricted to one, there are 699 shared single-copy APVO PlantTribes that are present in *Selaginella *and 558 shared single-copy APVO PlantTribes that are present in *Physcomitrella*. Even when considering the distant algal species *Chlamydomonas reinhardtii*, 362 shared single-copy APVO PlantTribes are present in *Chlamydomonas *with one or two copies. When the TIGR Plant Transcript Assemblies are considered, which include a wide range of green algae and terrestrial plants, 438 APVO PlantTribes have at least one hit in a non-seed land plant species and 190 APVO PlantTribes have at least one hit in a green algal (chlorophyte or charophyte) species (Table 1 and also additional file 4). Therefore, large subsets of the shared single-copy PlantTribes might have been maintained in diverse photosynthetic eukaryotic organisms for as many as 1,000 million years.

**Table 1:** Shared single copy nuclear genes are present throughout plant lineages

Taxonomic Group	Number of single copy APVO PlantTribes present
Eurosids	913
Asterids	519
Core Eudicots	76
Basal Eudicots	189
Monocots	948
Basal Angiosperms	48
Gymnosperms	502
Vascular Plants	438
Green Algae	190

Summary of additional file 4 with total number of shared single copy nuclear genes present and distribution of count numbers for various taxonomic groups. The eurosids include members from both Rosids I and II; core eudicots are comprised of families considered basal to either the Eurosids or the Asterids such as the Caryophyllales and the Vitales; basal eudicots are represented by the ranunculids *Eschscholzia californica *and *Papaver somniferum*; Monocots include both members of the Poaceae and non-grass monocots; basal angiosperms include members of the magnoliids as well as *Amborella trichopoda *and *Nuphar advena*; gymnosperms includes conifers, cycads, *Ginkgo *and Gnetales; Vascular plants include representatives from ferns, mosses and hornwort; and green algae include members of both chlorophyte and charophyte green algal lineages.

### Shared single copy genes have distinguishing characteristics compared to the rest of the genome

**Figure 2:**
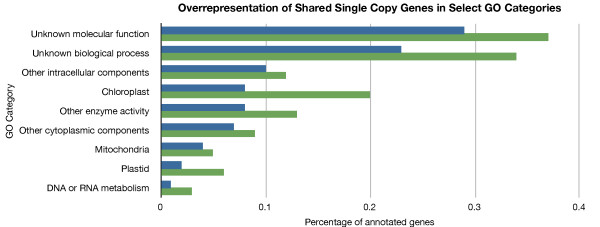
**Overrepresentation and underrepresentation of shared single copy genes in select GO categories**. Bar chart showing GO slim categories that are overrepresented in the APVO PlantTribes using the TAIR8 annotation of the *Arabidopsis thaliana *genome using an initial alpha value of 0.05 with a subsequent Bonferroni correction for multiple tests. Bottom green bar represents percentage of APVO shared single copy genes with the given annotation; top blue bar is percentage of genes with the given annotation for the remainder of the genome. Overrepresentation and underrepresentation was detected using a chi-square test comparing the slim GO annotation of the single copy tribes versus all else in the *Arabidopsis *genome.

To obtain clues of possible functions of the shared single copy genes, we analyzed their GO slim categories (Figure 2). The APVO shared single copy genes are overrepresented in the following GO slim categories: chloroplast, mitochondria, plastid, other intracellular components, other cytoplasmic components, other enzyme activity, DNA or RNA metabolism, unknown molecular function and unknown biological processes. Shared single copy genes are significantly underrepresented in several GO categories: other membranes, unknown cytoplasmic components, nucleus, transporter activity, nucleic acid binding, other molecular functions, transcription factor activity, kinase activity, other biological processes, protein metabolism, transport, transcription, response to abiotic or biotic stimulus and signal transduction. This corresponds with findings that retained duplicates tend to encode proteins with known functions such as subunits of macromolecular complexes (e.g. structural constituents of ribosomes), proteins with regulatory functions (e.g. transcription factors), and highly-connected signaling components especially those with opposing functions in networks (e.g. kinase and phosphatase) [8,74-76]. These results also mirror the result obtained by analysis of conserved ortholog sets (COSII) identified in the euasterid clade that indicate an association of single copy genes with genes targeted to the chloroplast and mitochondria, as well as genes associated with DNA and/or RNA metabolism [6].

Shared single copy genes from APVO PlantTribes have more exons than other genes (p < 0.00001, 2-tailed Student's t-test with unequal variances). This result holds when intronless, possibly retrotransposed genes are excluded from the analysis. Another notable distinction of the APVO PlantTribes is the smaller number of PFAM domains present in the shared single copy genes relative to the other genes in the sequenced genomes (p < 0.00001, 2-tailed Student's t-test with unequal variances). However, there is no significant difference in cDNA length (p = 0.998, 2-tailed Student's t-test with unequal variances). In summary, the shared single copy genes are structurally complex genes, with a greater number of exons present in the coding sequence. However, they do not appear to be functionally complex, as they have fewer recognized domains per gene than the rest of the genome.

### Shared single copy genes can be valuable phylogenetic markers for plant families

For successful use of these shared single copy genes in molecular systematics, it must be demonstrated that they can be easily amplified and sequenced in a range of taxa. In order to test this, we designed primers to amplify 12 genes in the Brassicaceae. When cDNA templates were used with degenerate primers, single bands of identical size were recovered across all of the taxa that yielded amplicons (Table 2). Direct sequencing of these loci yielded sequences with minor polymorphisms. These polymorphisms could be allelic or from multiple loci. In contrast, when genomic DNA was used as templates, 1 to 3 bands were recovered (Table 2). These genomic bands varied in size both across and, when more than one was detected, within the taxa. We amplified these 12 genes only for a subset of taxa to survey copy number variation in the family. The sequences of the RT-PCR amplification products were used for phylogenetic analysis. Sequence of a number of the genomic bands revealed either additional genomic copies that have pseudogenized, or completely unrelated sequence, but only one expressed copy that corresponded to the sequence from the RT-PCR derived sequence (results not shown).

**Table 2:** Amplification of shared single copy nuclear genes in Brassicaceae

Marker	At2 g21870	At2 g32520	At3 g47810	At4 g31720	At4 g33250	At5 g47570	At5 g63135	At5 g63135	At2 g13360	At4 g15790	At4 g37830	At5 g23290
**RT-PCR**												

***Brassica rapa***	1	1	1	1	1	1	1	1	1	0	1	1

***Brassica oleracea***	1	1	1	1	1	1	1	1	1	0	1	1

***Moricandria arvensis***	1	1	1	1	1	1	1	1	1	0	1	1

***Brassica repanda***	1	1	1	1	1	1	1	1	1	1	1	1

***Erucastrum canariense***	1	1	1	1	1	1	0	1	1	1	0	1

***Schouwia thebaica***	1	1	1	1	1	1	0	1	1	1	0	1

***Sisymbrium irio***	1	1	1	1	1	1	1	1	1	0	1	1

***Arabidopsis thaliana***	1	1	1	1	1	1	0	1	1	0	1	1

***Olimarabidopsis pumila***	1	1	1	1	1	1	0	1	1	0	1	1

***Chorispora tenella***	1	1	1	1	1	1	0	1	1	0	1	1

***Athionema saxatile***	1	1	1	1	1	1	0	1	1	1	0	1

***Medicago truncatula***	1	1	1	1	1	1	0	1	1	1	0	1

**Band Size in bp**	450	450	350	250	210	300	250	300	900	150	150	150

**PCR**												

***Brassica rapa***	3	1	1	3	3	3	2	1	1	1	1	2

***Brassica oleracea***	2	1	1	2	2	2	1	1	1	2	1	1

***Sisymbrium irio***	1	1	0	1	1	1	1	1	1	1	1	2

***Arabidopsis thaliana***	1	1	1	1	1	2	1	1	1	1	1	2

***Olimarabidopsis pumila***	1	1	0	1	2	1	2	1	1	1	1	2

**Band Sizes in bp**	700	1000	2000	1300	1200	2000	2000	3000	500	1000	1500	700
		800	600	600	1100	700	400	300	200	500		
		700					200					

PCR and RT-PCR amplification results from Brassicaceae showing number and size of bands. The number of bands amplified is indicated (0-5).

**Figure 3:**
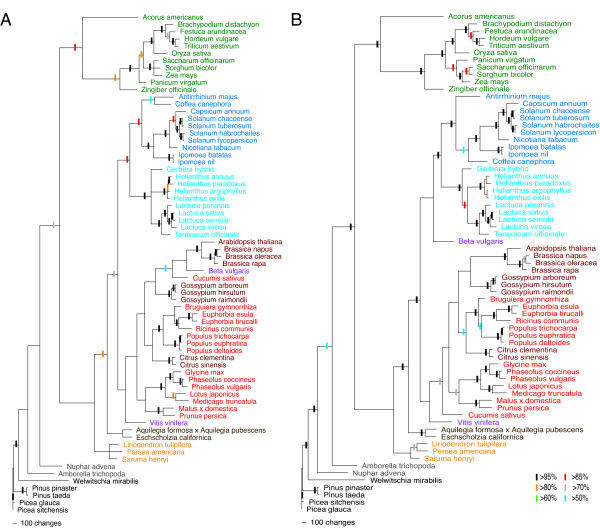
**Single copy nuclear genes improve phylogenetic resolution in the Brassicaceae**. A single most-parsimonious tree was found from combined analysis of complete data-matrix containing genes At2 g32520, At2 g13360, and At5 g23290 (L = 961, consistency index = 0.774, retention index = 0.529). Bootstrap values are shown above branches. Note that individual gene trees gave similar topologies. The phylogeny is consistent with published phylogenies using more taxa and other molecular markers. Bootstrap values from Beilstein et al., 2006 are shown below branches.

A combined and complete data-matrix containing genes At2 g32520, At2 g13360, and At5 g23290 homologs for each of the twelve taxa yielded a single most-parsimonious tree with the exact predicted and previously supported topology (Figure 3) [77-79] but with increased bootstrap support at deeper nodes than previously published phylogenies. The tribe Brassiceae had a 100% bootstrap statistical support and a 100% bootstrap support that the tribe is sister to the tribe Sisymbrieae. The Brassiceae + Sisymbrieae clade located within lineage II, had a 99% bootstrap support to the relative position to the Camelinae tribe located within lineage I, and the lineage I and lineage II clade had a 100% bootstrap support for a sister relationship to Chorisporeae (lineage III).

### Shared single copy genes are valuable phylogenetic markers across angiosperms

As mentioned briefly above, homologs of the shared single copy genes are well represented in the Plant Transcript Assemblies [73], which at the time of this analysis include over 13 million plant ESTs from 233 species. Given this rich resource of sequence information, we examined in more detail the presence and distribution of homologs of shared single copy genes in this dataset. We compare the EST data and Unigene numbers with the known gene copy number from the *Arabidopsis*, *Populus*, *Vitis *and *Oryza *genomic sequences, and found that the numbers of Unigenes vary substantially from gene copy numbers from the genome sequences - most likely resulting from sequencing error, assembly errors, number of ESTs, alternative splicing, allelism and other such factors. Using a set of criteria (see Methods) designed to maximize sequence coverage and quality for each dataset, we used phylogenetics to help distinguish between species-specific duplicates (which could result from either technical error, alleles or an actual recent duplication event) and shared duplication events that could reveal if there are patterns of maintenance of duplicate copies in particular lineages. Because this process requires extensive manual inspection of the alignments, a limited number (eighteen) of genes were selected for the phylogenetic analysis. These eighteen genes were selected because there were full-length EST sequences available across the major lineages of angiosperms.

We found that single gene phylogenies provide well-supported resolution at the level of plant families (Table 3). Specifically, the large plant families Fabaceae, Poaceae, Asteraceae, and Solanaceae, all of which are well represented in the Plant Transcript Assemblies, are strongly supported in the single gene phylogenies (Additional File 3). Shared duplications in a particular lineage are also present in five of the individual gene phylogenies; these genes were excluded from the subsequent concatenated alignment. Such shared duplications within a lineage are associated with paleopolyploidy events that occurred within particular lineages [1,3]. It should be noted that even though many members of Poaceae are well represented in the Plant Transcript Assemblies and multiple sequences from a single species were typically included in the single gene alignment, there were no cases of shared duplications within this plant family - multiple sequences from the same species were more closely related to each other than to sequences from other species (Additional File 3). Moreover, there are no duplications that involve more than a single plant family - evidence for duplications that occurred prior to diversification of the extant plant families is not present in these datasets. This supports the idea that duplicates of these genes do not persist over evolutionary time and the presence of more than one gene copy within a taxon is most likely the result of relatively recent duplication events. If gene loss did not occur soon after duplication, however, it is possible that the genes retained in different lineages are paralogous. This may result in well-supported conflict among gene trees. In our analyses, contradictions to the general consensus concerning angiosperm phylogeny, such as *Acorus *falling within magnoliids instead of a basal position within monocots, have poor bootstrap support (<70%).

**Table 3:** Shared single copy nuclear genes are a rich source of phylogenetic information.

ATH	Annotation	# SEQ	# NT	# VAR	PI	>50 MP	>50 ML
At2 g13360	AGT1	49	1203	703	51%	63%	65%
At3 g47810	MAIGO 1	91	573	359	49%	66%	73%
At2 g32520	dienelactone hydrolase family protein	73	721	536	64%	75%	81%
At3 g52300	ATPQ	129	519	395	62%	65%	66%
At5 g06360	Ribosomal protein S8e	51	780	449	47%	58%	58%
At5 g04600	RNA recognition motif (RRM)-containing protein	63	579	475	74%	68%	71%
At2 g21870	probable atp synthase 24 kda subunit, mitochondrial precursor	95	606	492	68%	78%	78%
At4 g33250	eukaryotic translation initiation factor 3 subunit 11 (eif3k)	60	662	453	59%	68%	66%
At4 g30010	fiber protein Fb15	129	251	217	77%	62%	63%
At1 g77710	Probable ubiquitin-fold modifier 1 precursor	143	254	202	55%	46%	45%
At4 g08230	glycine-rich protein	51	413	286	54%	60%	68%
At4 g31720	STG1, TAFII15	64	448	323	51%	63%	67%
At4 g37830	putative cytochrome c oxidase subunit VIa precursor	151	216	192	71%	43%	45%
At5 g47570	similar to hypothetical protein 25.t00006 [Brassica oleracea]	90	387	288	64%	58%	65%
At5 g23290	putative c-myc binding protein	69	404	289	64%	62%	69%
At1 g27530	expressed protein	60	525	332	51%	68%	71%
At3 g20390	Endoribonuclease L-PSP, putative	132	372	313	69%	70%	70%
At5 g63135	hypothetical protein	64	448	323	51%	65%	63%
	Concatenated alignment of 13 shared single copy genes	69	7701	5072	55%	81%	87%

This table provides the following information about the 18 shared single copy genes that were used for phylogenetic analysis based on EST and finished cDNA sequences: the *Arabidopsis thaliana *locus ID (ATH); the TAIR annotation in *Arabidopsis *(Annotation); the number of sequences in the final alignment (# SEQ); the number of nucleotides in the final alignment (# NT); the number of variable characters in the final alignment (# VAR); the percentage of characters that are parsimony-informative (% PI); the percent of nodes in the MP bootstrap consensus trees that are supported in greater than 50% of the bootstrap replicates; the percent of nodes in the ML bootstrap consensus trees that are supported in greater than 50% of the bootstrap replicates.

**Figure 4:**
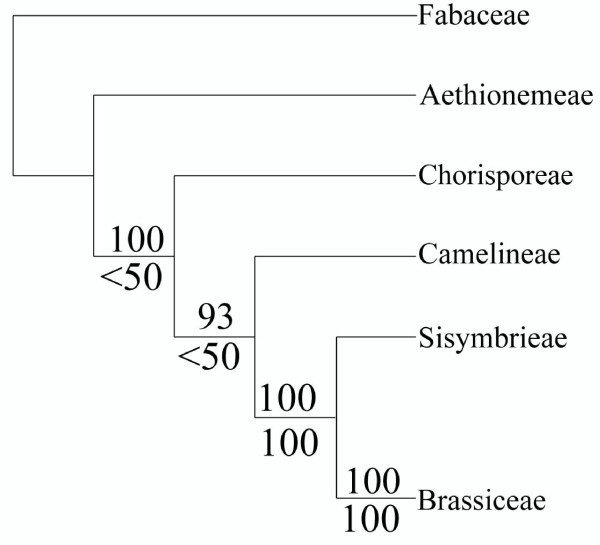
**Angiosperm phylogeny using ESTs for 13 shared single copy genes**. The tree depicted to the left is the MP tree determined from the concatenated data matrix for 13 single copy genes using 69 seed plant taxa. The tree depicted on the right is the ML tree determined from the concatenated data matrix for 13 single copy genes using 69 seed plant taxa. Bootstrap values are indicated by the colored bars placed on branches with greater than 50% bootstrap support. *Picea sitchensis *was used as the outgroup taxa for all analyses. Taxa are color-coded as follows: monocots (green); euasterid I (light blue); euasterid II (dark blue); eurosid I (pink); eurosid II (red); core eudicot (purple); basal eudicot (brown); magnoliid (orange); basal angiosperm (dark gray); gymnosperms (black).

In contrast to the individual gene phylogenies, phylogenetic trees based on a concatenated alignment of 13 of the shared single copy genes (Figure 4) show improved resolution and are similar to recently reported phylogenies [61,62,80,81] though there are significant differences with the placement of individual species between the MP and ML trees. Overall, the ML tree shows improved resolution and increased bootstrap support compared to the MP tree (Table 3). The eurosids, asterids, and monocots are all resolved as monophyletic (Figure 4). All plant families with multiple species present in the datasets are resolved as monophyletic with strong bootstrap support. In all cases in which multiple members of a genus are included in the analysis, genera are resolved as monophyletic with strong bootstrap support. Eurosids I and II are paraphyletic with the Sapindales (Eurosid II) sister to a clade containing the Malpighiales (Eurosid I) with poor bootstrap support (below 50% in MP and 53% in ML). Aside from the placement of the Malpighiales, the rest of the eurosid II lineage is monophyletic. Vitales are basal to the eurosids with poor bootstrap support (<50%). Euasterids I and II are both monophyletic with strong bootstrap support. The position of the caryophillid *Beta *is not stable, nesting within the eurosid II clade in the MP phylogeny and sister to the Asterids in the ML phylogeny, with below 50% bootstrap support for both placements. Ranunculales are sister to all core eudicots with strong support in the ML analysis and moderate support in the MP analysis. Within the monocots, *Acorus *is basal, with *Zingiber *sister to the Poaceae. Limited resolution at basal nodes, such as the affiliation of the core eudicots, magnoliids and basal angiosperms, is expected to be the result of the limited amount of sequence information available for these lineages. Magnoliids are sister to the eudicots, with poor bootstrap support, contrary to their placement sister to the monocots + eudicots in whole plastid genome datasets [61,62]. The ordering of *Amborella *and *Nuphar *switches between the MP and ML tree, with below 50% support for *Amborella *as the basalmost angiosperm in the MP phylogeny and with 52% bootstrap support for *Nuphar *as the basalmost angiosperm in the ML phylogeny. Gymnosperm relationships are un-informative given the rooting of the tree with *Picea sitchensis *and the limited taxon sampling since there was not a sufficient coverage of the target genes for members of the Zamiales, Ginkgoales and Gnetales.

## Discussion

### There is a large number of shared single copy nuclear genes in plant genomes

Because of pervasive gene duplication in the angiosperms, the observation of hundreds of shared single copy nuclear genes in the sequenced genomes we have examined raises interesting biological questions. It is unlikely that any gene identified in this study is single copy in every extant angiosperm, given the frequency of gene duplication and the time required to resolve gene duplication events. However based on the skewed distribution towards certain GO terms (e.g. plastid and mitochondrial functions) for this list of shared single copy nuclear genes, the data suggests that the list may contain a set of single-copy genes that are not shared at random (i.e. conserved single copy genes). This will be discussed in more detail in subsequent sections. Overall, this study provides hundreds of genes for which further characterization and research could greatly improve our understanding of the molecular evolution of genes in flowering plants, the functional and evolutionary impact of gene duplication, and provide new tools for plant biologists, especially in the realm of molecular systematics.

### Shared single copy genes have different characteristics than singletons

A previous study on singletons present in the *Oryza *and *Arabidopsis *genomes [7], but not necessarily conserved as singletons between the two genomes yielded results that do not correspond to the findings presented in this paper. The singletons presented in Chapman et al. [7] are significantly shorter than genes with a duplicate. However, our analysis of *Arabidopsis *genes indicates that there is no significant difference in cDNA length between shared single copy genes and all other genes. To some degree, the difference in approach for identifying single copy genes may provide a clue as to the difference in results. Chapman et al. [7] identified single copy genes (singletons) in individual genomes, whereas the single copy genes in this study were identified by requiring the gene to be single copy in four genomes as an added criterion. This suggests that there may be significantly different molecular characteristics for shared and non-shared singletons within an individual genome as a result of differential molecular evolution of these two subsets of single copy genes. It is possible that some of the singletons identified by Chapman et al. [7] are unique to their genome or lineage, or that the homologous sequence in another genome has paralogs. The only common characteristic between the shared singletons and the non-shared singletons is a decreased number of protein domains - however this could be the result of poor characterization and annotation of genes that are not members of large gene families. It is of note that the functional overrepresentation of shared single copy genes in slim GO categories is highly similar to the results found by Wu et al [6] for clusters of singletons in the Asterids that were verified as single copy in *Arabidopsis*, which they named conserved ortholog sets (COSII). Therefore, there could be significant similarities between genes that experience nonrandom gene loss after duplication and genes that experience random genes loss after duplication.

An increased average number of exons in shared single copy genes would be consistent with a hypothesis that intron loss is facilitated by retrotransposition and gene conversion in gene families [82], but this idea remains speculative, and should be subjected to rigorous testing. In the absence of paralogs, we hypothesize that introns are more stable in shared single copy genes than introns in genes that are members of gene families. This aspect of intron evolution in shared single copy genes could be very important for their utility in phylogenetic studies, since intronic sequences can be a useful tool for species identification and classification.

### Evolution of shared single copy nuclear genes against a background of polyploidy

The reduced number of single copy genes found when *Populus *was included in the analysis can be attributed to the relatively recent genome duplication in *Populus *together with the slower rate of evolution in the woody plant *Populus *relative to rice and *Arabidopsis *[83]. Substantial differences in the rates of molecular evolution in different plant lineages observed by Smith and Donoghue could lead to differences in how long it takes for a duplicated gene to return to single copy through either through random or unknown nonrandom (selective) processes. Some portion of this can be attributed to the degeneration of one of the duplicates according to neutral evolution and population genetics parameters [84]. However, the conservation of many of these genes as single copy throughout the angiosperms, as evidenced by their representation and distribution in the broad EST-based phylogenies from this study, suggests that nonrandom processes could be involved in maintaining their single copy status. For example, variance in copy number may relate to variance in the strength of negative selection against more than one copy after duplication. It is also plausible that a number of these shared single copy genes are only coincidently single copy in more than one genome, having returned to single copy after duplication through genetic drift if there is selection to maintain most genes as duplicates following genome duplication [72]. Unfortunately, the small number of sequenced genomes and incomplete functional characterization for a number of these genes does not allow us to make a firm conclusion on which evolutionary forces predominate in the evolution of these genes or whether any given gene is single copy as the result of random loss or selection. Ideally, functional characterization and population genetic studies of this class of genes as well as more sequenced genomes will in the future provide more evidence about the respective roles of selection and drift on the evolution of these genes after duplication.

The strong association between organellar (chloroplast and mitochondria) targeted gene products and the shared single copy genes identified in this study supports a hypothesis that coordination of protein complexes such as those involved in electron transport in both the plastid and mitochondrion requires an evolutionarily-stable copy number, since these nuclear-encoded proteins interact with organellar-encoded proteins. However it is unclear how dosage in the nuclear genome (one nucleus per cell) and dosage in the plastid genome (many plastids per cell, each containing many plastid genomes) is coordinated. This hypothesis extends the gene balance hypothesis by invoking the specific complexity of protein complexes that include members from both the nuclear genome and the organellar genomes [85-88].

Although some of the genes identified in this study may be present as single copy as the result of random processes, additional data obtained from our phylogenetic analysis of EST sequences of a subset of these genes from a wide range of angiosperms suggests that the single copy status of some of these genes is non-random. Cases that are indicative of ancient duplication are limited to clades for which ancient polyploidization events have been hypothesized that coincide with the duplication of single copy genes, such as the Asterids, Fabaceae, and *Gossypium *(see Additional File 3). By focusing on sequences obtained through transcriptome sequencing or RT-PCR, we have focused on expressed copies of these single copy genes. However, we hypothesize that in genomes with recent duplication events there will be multiple loci present for any gene, including the shared single copy genes. It may take millions of years for duplicated loci to resolve to a single locus - however, silencing of duplicated loci such that only a single locus is actively expressed is expected to occur relatively quickly after duplication [47,89-91].

The expressed copy of At2 g32520 was aligned to non-expressed genomic DNA copies for several taxa in the Brassicaceae that were cloned and sequenced. This comparison revealed not only which genomic DNA copy was expressed but also that the non-expressed copies displayed evidence of pseudogenization by having acquired numerous point mutations, insertions, and deletions likely resulting in gene loss through sequence degeneration. Many of the taxa are relatively recent polyploids, and it is difficult to conclude if the few polymorphisms observed in some of the sequences from the directly sequenced bands from RT-PCR are allelic or from duplicate copies. However, this analysis did display putative evidence for the loss of duplicate copies from past duplication events, inferred from the presence of multiple pseudogenized genomic loci.

These previous observations suggest that these 'single copy' genes have convergently returned to a single copy state following whole genome duplication events. The individual gene trees show this pattern, as well as illustrating differences in the resolution of whole genome duplication events. All of the inferred gene duplications present in the individual gene trees are coincident with whole genome duplications that have been demonstrated by either K_s _distributions of ESTs or syntenic chromosomal blocks [1,3,15,16,69]. In lineages where tandem or segmental duplication is inferred to be more prevalent than whole genome duplications, such as the monocots (specifically the Poaceae [16]), multiple sequences from a single species are more closely related to each other than sequences from a different species. This indicates that if duplicates are actually present in a genome (not detectable using ESTs), they are recent in origin and did not originate during a shared duplication event. Overall, these results suggests that typically genome-scale duplications are associated with maintenance of more than one locus for this class of genes - however more research is required to determine if this trend extends to all shared single copy genes, since the phylogenetic analysis was limited to a small number of shared single copy genes. However, it must be determined if genes within this set are in fact dosage-sensitive (i.e. conserved single copy genes that are under selection to return to single-copy) or dosage-insensitive (i.e. returned at random to single-copy) in order to understand whether the gene balance hypothesis is applicable.

### Shared single copy genes are a valuable new source of phylogenetic information from the nuclear genome

This study emphasizes the utility and ease of using single copy APVO PlantTribes for phylogenetic studies. The Brassicaceae has been recently divided into 25 tribes that are mostly grouped into four monophyletic lineages [77,79]. The tribe Aethionemeae containing *Aethionema saxatile *is the tribe sister to the remainder of the family [77-79]. Lineage I contains eight tribes, including the tribe Camelineae with *Arabidopsis thaliana *and *Olimarabidopsis pumila*. Lineage II contains four tribes, including the agronomically important tribe Brassiceae with *Brassica rapa*, *Brassica oleracea*, *Brassica repanda*, *Moricandria arvensis*, *Schouwia thebaica*, and *Erucastrum canariense*. The tribe Brassiceae is thought to have an ancient hexaploid event, while the sister tribe Sisymbrieae represented by *Sisymbrium irio *does not share this ancient polyploidy event [69]. Lineage III includes four primarily Asian tribes including the tribe Chorisporeae and there are also tribes that have not been assigned to any of these three lineages [77]. The combined and complete data matrix of three single copy nuclear genes (At2 g32520, At2 g13360, and At5 g23290) yielded a single most-parsimonious tree with strong bootstrap support that is consistent with published phylogenies using more taxa and other molecular markers in the Brassicaceae [77-79]. Individual gene trees gave similar topologies.

Although individual gene trees do not provide sufficient resolution for deep nodes across the seed plant phylogeny (Additional file 3), the combined data matrix of thirteen single copy nuclear genes across 69 seed plants yields a phylogenetic tree with good resolution, given the overall size of the data matrix and the amount of overlap in sequence between different taxa. In a general sense, the resulting phylogeny from the combination of these thirteen single copy genes agrees with recent results from large datasets based on whole plastid genome sequences [61,62]. It is expected that improved sampling at basal nodes would improve the resolution of the resulting phylogeny, since current EST resources for these taxa are considerably less than EST resources available for crop species in the Poaceae and eurosids, for which coverage is excellent. An important result from the EST-based phylogenies is that these nuclear genes are a rich source of phylogenetic information - they include a high percentage of variable and parsimony-informative characters, and extended branch lengths at deeper nodes within the trees (Table 3). Although the dataset presented in this study is limited to small number of genes, the methodology presented of using shared single copy genes for phylogenetics using sequences from public databases could be expanded to utilize all of the shared single copy nuclear genes with automation of the alignment and sequence selection methods.

Our focus on shared single copy genes reflects an experimentally tractable and relatively conservative approach to leveraging the phylogenetic information present in large sequence datasets. The Tribe-based approach we describe here could readily be extended to identify and incorporate genes from stable lineages within any gene family. For example, preliminary phylogenetic screening of stable tribes with 2, 3, 4, or more genes in a Tribe could be used to indentify putatively orthologous sets of genes that are reciprocally monophyletic and repeatedly estimate the organismal phylogeny.

We predict that in contrast to other studies which have used alternative clustering methods and have incorporated sequences for which paralogs are present, high-throughput phylogenetics using shared single copy genes will exhibit a stronger signal to noise ratio and minimize conflict within the analysis. However, it remains to be tested how results from high throughput phylogenetics utilizing only shared single copy genes will compare to results that include genes that are members of gene families. Such inclusion will greatly expand the quantity of data that can be included from genome and transcriptome sets. The continuing decline in the cost of high throughput "Next Generation" sequencing methods ensures that many more large scale transcriptome datasets will become available in the near future for these and other genome-enabled approaches to plant phylogenetics.

## Conclusions

The identification of shared single copy nuclear genes is important for many reasons, given their unique status in genomes full of paralogs. Even though their origin and molecular evolution are of continuing interest, shared single copy nuclear genes have a series of immediate, valuable applications as mapping markers, quality control for genomic libraries, and phylogenetic markers. As single copy loci, these genes can provide important genomic landmarks for mapping of other genes or features in the genome, and can be used as control sequences to identify the amount of coverage provided by a given genomic or transcriptomic library. However, their potentially greatest impact application is for molecular phylogenetics within the angiosperms, and perhaps deeper, since most are found in a diverse set of angiosperm EST studies (Additional file 2) and approximately half can be detected in the non-seed plant lineages (Additional file 1).

Since most current molecular systematic studies in angiosperms are based on plastid and non-coding nuclear sequences, it is important to know whether protein-coding nuclear genes provide complementary support to these studies, or conflict. The complexity of gene families in angiosperm genomes has deterred researchers from using nuclear genes in phylogenetic reconstruction in angiosperms on a regular basis because of the difficulty of isolating all members of a gene family using laborious cloning methods in each taxon to obtain accurate phylogenetic reconstructions [4]. Given the dynamic evolutionary history of plant nuclear genes as a result of recombination, gene conversion, duplication and endosymbiotic gene transfer, it is not yet clear whether phylogenies based on nuclear-encoded proteins will be congruent with phylogenies based on plastid-encoded proteins. However, the results from this study show no compelling evidence of a conflict between phylogenies derived using plastid data and the phylogenies presented. By comparing and combining data from single copy nuclear genes with plastid genes, mitochondrial genes, and ribosomal DNA markers, we will have a better understanding of the diversification of flowering plants.

Shared single copy nuclear genes are an important tool for understanding angiosperm phylogeny and are applicable across a wide range of taxonomic levels. Both coding and intron sequences from these genes are phylogenetically informative and genes can be easily amplified and sequenced using RT-PCR or PCR, or extracted from genomes or EST databases. It is now necessary to expand our knowledge of these genes by sampling them in diverse lineages in order to fully appreciate their phylogenetic utility.

## Methods

### Identification of shared single copy nuclear genes in sequenced plant genomes

We identified shared single copy nuclear genes using a high throughput comparative genomic approach, using available data from the *Arabidopsis*, *Populus*, *Vitis *and *Oryza *genomes. The *Arabidopsis*, *Populus*, *Vitis *and *Oryza *proteomes (as described in [63]) were compared against each other using "all by all" BLASTP [92]. Genes were clustered into 'tribes', an approximation of gene families, using TRIBE MCL [63,93,94] at medium (Inflation I = 3.0) stringency. This procedure uses the graph-based Markov clustering algorithm MCL, in which a transformation of the symmetric matrix containing the average pairwise -log10E-values is taken through a series of matrix multiplication and inflation steps. The result is an objective global classification of proteins from the combined sample. Additional details and validation studies of the classification procedure are provided in Enright et al, 2002, Enright et al. 2003, and Wall et al. 2007 [63,93,94]. Wall et al. provide two specific examples of the relationship between tribe stringency and the hierarchical structure of tribes [63]. The focus of this study - shared single copy genes - were identified as the set of unique clusters emerging from the TRIBE MCL classification that included exactly one gene from each genome included in the analysis. The collection of tribes with exactly one gene from *Arabidopsis*, *Populus*, *Vitis*, and *Oryza *are termed the APVO single copy (SC) tribes.

### Identification of homologs of APVO shared single copy nuclear genes in non-seed plants

To identify the presence of shared single copy genes in non-seed plants, we used the PlantTribes database ([63]; http://fgp.huck.psu.edu/tribe.html) to identify shared single copy genes in *Selaginella*, *Physcomitrella*, *Arabidopsis*, *Populus*, *Vitis *and *Oryza*. In addition, the presence of genes in the *Physcomitrella*, *Selaginella *and *Chlamydomonas *genomes that are shared single copy genes in *Arabidopsis*, *Populus*, *Vitis*, and *Oryza *was also identified. The PlantTribes database identifies clusters of genes from sequenced genomes through TRIBEMCL clustering of BLASTP searches against various combinations of plant genomes [63,93,94]. The presence of shared single copy genes in other non-seed plant lineages were detected by using top hits to TBLASTX of all shared single copy *Arabidopsis*, *Populus*, *Vitis *and *Oryza *protein sequences against the Plant Transcript Assemblies ([73]; http://plantta.tigr.org/).

### Characterization of shared single copy nuclear genes in *Arabidopsis*

To characterize broadly the shared single copy genes shared between *Arabidopsis*, *Populus*, *Vitis *and *Oryza*, we analyzed the genes based on their attributes in *Arabidopsis *for the following characteristics: 1) slim Gene Ontology (GO) categories for biological processes, molecular function, and cellular location; 2) cDNA length; 3) number of exons; and 4) number of domains as compared to all other genes in the *Arabidopsis *genome. For GO classifications we compared the APVO single copy genes against all other genes using χ^2 ^tests to detect overrepresentation and underrepresentation of single copy genes in the slim GO categories as compared to all other genes, using a p-value cutoff value of 0.05, adjusted using a Bonferroni correction for multiple tests for each separate category within the GO classification (cellular location, molecular function, biological process). Differences in coding sequence length, number of exons, and number of domains between shared single copy genes and all other genes were tested using a Student's t-test and a p-value cutoff of 0.01.

### EST-based angiosperm phylogeny using shared single copy nuclear genes

In order to explore the phylogenetic utility of conserved single copy nuclear genes, eighteen genes from the *Arabidopsis *and *Oryza *shared single copy PlantTribes with good taxonomic sampling of full-length sequences across the angiosperms were selected for further study (Table 3). When available, cDNA clones from select taxa (*Acorus americanus*, *Amborella trichopoda*, *Cucumis sativus*, *Eschscholzia californica*, *Liriodendron tulipifera*, *Nuphar advena*, *Persea americana*, *Ribes americanum*, *Saruma henryii*, *Vaccinium corymbosum*, *Welwitschia mirabilis*, *Yucca filamentosa*, *Zamia vazquezii*) were sequenced on both strands to obtain a high-quality finished sequence for phylogenetic analysis. Internal primers were designed using MacVector (Accelrys, San Diego, CA) when necessary. Additional sequences for phylogenetic analysis were obtained from the Plant Transcript Assemblies [73] by using TBLASTX [95] to identify putative homologs using both the *Arabidopsis *and *Oryza *genes as query sequences.

Amino acid sequences were aligned using MUSCLE [96] and nucleotide alignments were created by forcing the DNA sequence onto the protein alignment using CLUSTALW [97]. Alignments were manually inspected and adjusted. Sequences were eliminated from the alignments according to the following criteria: 1) the sequence was from a non-seed plant such as a fern, moss or an alga; 2) the sequence contained at least five ambiguous bases (N); 3) the sequence had low sequence similarity compared to the rest of the alignment; 4) 50 or more bp were missing from either the 5' or 3' end of the alignment, anchored by the start codon at the 5' end and the stop codon at the 3' end; 5) the sequence was identical to another sequence (preference given to transcript assemblies over EST singletons); 6) the sequence was from a hybrid for which close relatives were also included in the alignment. Gaps shared between species were included in the final alignments, whereas gaps that occur only in a single sequence were removed. A concatenated alignment of 13 shared single copy nuclear genes was constructed for 69 taxa using the following criteria: 1) genes were included if the single gene phylogenies presented no evidence of shared duplication events; 2) taxa were included if at least 6 of the gene sequences were available for that taxon; 3) a single sequence for each species was selected with preference for plant transcript assemblies with the greatest amount of coverage and the shortest branch length. Shared duplication events and identical sequences were identified through the examination of a neighbor-joining phylogenetic tree and accompanying distance matrix that was generated using Jukes-Cantor distance in PAUP 4.0b10 [98] applied to the nucleotide alignment for each individual after non-seed plant sequences, truncated sequences, low similarity sequences, and sequences with more than 5 ambiguous bases were eliminated from the alignment. Nucleotide alignments after all criteria were applied were used to produce phylogenies using maximum parsimony (MP) and maximum likelihood (ML). Nucleotide alignments can be found in Additional file 5. Appropriate models of sequence evolution for ML analyses were determined using MODELTEST using Aikake's Information Criteria [99]. Maximum parsimony searches and bootstrapping were completed using a heuristic search in PAUP 4.0b10 [98] with 500 ratchet iterations as implemented by PRAP [100] with ten random sequence addition replications and tree-bisection-reconnection (TBR) branch swapping. Maximum likelihood searches and bootstrapping were completed in GARLI 0.951 using default settings for the genetic algorithm [101]. 1000 MP and 100 ML bootstrapping replicates using the same heuristic search parameters and model of sequence evolution were performed for each of the eighteen single copy PlantTribes examined, as well as the concatenated alignment of thirteen genes.

### Using shared single copy nuclear genes for phylogeny in the Brassicaceae

Ten taxa were selected in Brassicaceae to span the major lineages and to sample more intensively in the tribe Brassiceae that is thought to have an additional ancient polyploidy event. Five tribes were selected because of their known relative relationships to one another because the relationships between most of the other tribes remain relatively unresolved. The family Cleomaceae has been firmly established as the family sister to Brassicaceae [70,102] and *Medicago truncatula * (Fabaceae) was selected as an additional outgroup which is positioned within the rosid clade.

Total RNA was extracted from approximately 250 mg fresh young leaf tissue using the Invitrogen Homogenizer and the Micro- to Midi- Total RNA Purification System and then converted into cDNA using the Invitrogen SuperScript III First-Strand Synthesis System for RT-PCR kit (Invitrogen, Carlsbad, California) following all manufacturer's instructions. Polymerase chain reactions (PCR) amplifications were performed using degenerate primers designed by eye based on the EST alignments, Eppendorf Triplemaster PCR system, and the Eppendorf Mastercycler epgradient S thermocycler (Eppendorf, Hamburg, Germany) and following Eppendorf's high fidelity PCR protocols using a 60-62°C primer annealing temperature. Amplicons were analyzed by gel electrophoresis, purified using the Invitrogen Purelink PCR Purification kit, and than direct sequenced at the Core facility at the University of Missouri-Columbia.

The sequences (1594 characters with 320 Parsimony informative) were automatically assembled using the software SeqMan (DNA Star, Madison, Wisconsin), aligned using Megalign (DNA Star), and further aligned by eye using Se-Al v2.0a11 [103]. Alignments used for this analysis are available (Additional file 6). Maximum parsimony analyses of the alignments were conducted using PAUP 4.0b10 [98]. The most parsimonious trees were generated using the heuristic search algorithm, equal weighted characters, gaps treated as missing data, with tree bisection-reconnection (TBR) branch swapping, 1000 random additions of the sampled taxa, and 10 trees saved per replicate and a strict consensus tree was computed. Bootstrap analyses were performed with a 1000 replicates, full heuristic search, TBR branch swapping and simple sequence addition.

## Authors' contributions

JMD performed characterization of the shared single copy genes, manual curation of EST-based alignments, phylogenetic analysis of EST-based alignment and wrote the manuscript. PKW developed the PlantTribes database and developed software for gathering data for characterization and automated alignment for EST-based analyses. PPE designed primers, performed RT-PCR, sequencing, alignment and phylogenetic analysis for shared single copy genes in the Brassicaceae. LLL contributed to finished cDNA sequencing of single copy genes in select taxa. JCP conceived of the study using single copy genes for Brassicaceae phylogeny. JLM and CWD conceived of the study. All authors contributed ideas to the manuscript and have read and approved the final manuscript.

## Supplementary Material

Additional file 1 **Identification of all shared single copy nuclear genes among the *Arabidopsis*, *Populus*, *Vitis *and *Oryza *genomes, as well as the *Physcomitrella *and *Selaginella *genomes**. Counts for each gene in each genome are provided. Locus ID in *Arabidopsis*, as well as TAIR8 annotation for symbol, description and slim GO annotation in terms of molecular function, cellular location and biological process are also provided. All tribes can be located in the PlantTribes database at fgp.bio.psu.edu/tribe.php by searching with the appropriate *Arabidopsis *locus ID. Click here for file

Additional file 2 **BLASTx hits to the *Arabidopsis*, *Populus***. *Vitis *and *Oryza *conserved single copy nuclear genes for all plants in TIGR Plant Transcript Assemblies that have more than 5 hits to the APVO single copy PlantTribes, showing taxonomic grouping as well as gene counts. Plants in the Plant Transcript Assemblies are referred by their 5 letter abbreviation which is based on the first 3 letters of the genus name, followed by the first 2 letters of the species name. A full listing of the contents of the Plant Transcript Assemblies can be seen at http://plantta.jcvi.org/cgi-bin/plantta_release.pl. Click here for file

Additional file 3 **ML and MP trees for the 18 single copy genes based on alignments of EST and finished cDNA sequences are provided as well as the ML and MP bootstrap consensus trees**. MP bootstrap consensus trees and all ML trees are available as PDF files and the collection of MP trees for each alignment is available as a .tre file. Click here for file

Additional file 4 Coding sequence length, number of exons and number of domains for all *Arabidopsis *proteins according to the TAIR8 annotation, with genes identified as to whether they are a member of the APVO PlantTribes. Click here for file

Additional file 5 **Alignments for the 18 single copy genes using seed plant ESTs and finished cDNAs are provided in NEXUS format, in addition to the concatenated alignment of 13 single copy genes**. Click here for file

Additional file 6 Alignments used for Brassicaceae phylogeny. Click here for file
