# Supplementary material for: Identification of shared single copy nuclear genes in Arabidopsis, Populus, Vitis and Oryza and their phylogenetic utility across various taxonomic levels
Source: BMC Evol Biol. 2010 Feb 24;10:61. doi: 10.1186/1471-2148-10-61 (PMC2848037; doi:10.1186/1471-2148-10-61)
Supplement: Additional file 3 — ML and MP trees for the 18 single copy genes based on alignments of EST and finished cDNA sequences are provided as well as the ML and MP bootstrap consensus trees. MP bootstrap consensus trees and all ML trees are available as PDF files and the collection of MP trees for each alignment is available as a .tre file. [file 1471-2148-10-61-S3.ZIP › EST_Trees/EST_Trees.pdf]

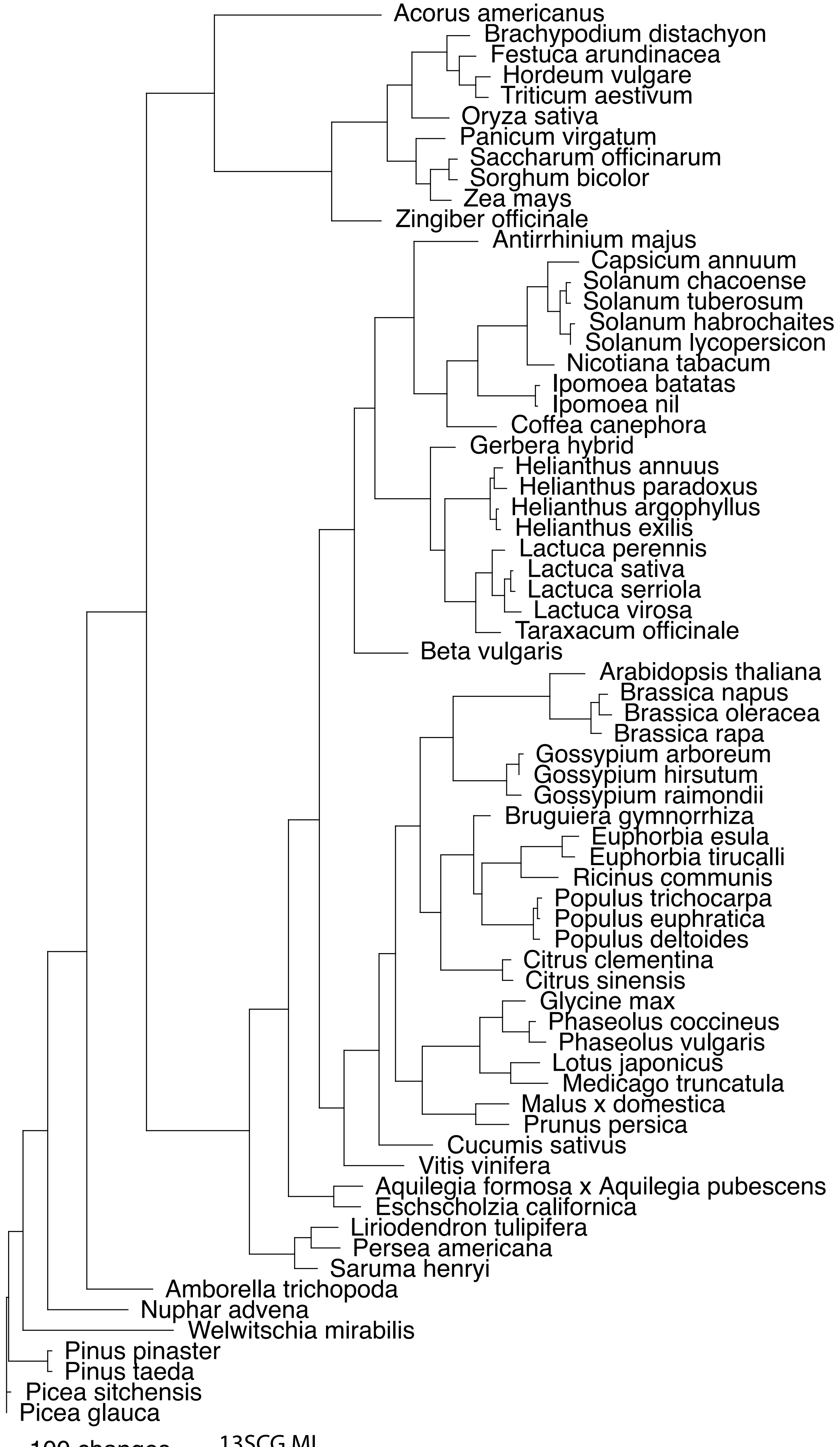
Acorus americanus
Brachypodium distachyon
Festuca arundinacea
Hordeum vulgare
Triticum aestivum
Oryza sativa
Panicum virgatum
Saccharum officinarum
Sorghum bicolor
Zea mays
Zingiber officinale
Antirrhinium majus
Capsicum annuum
Solanum chacoense
Solanum tuberosum
Solanum habrochaites
Solanum lycopersicon
Nicotiana tabacum
Ipomoea batatas
Ipomoea nil
Coffea canephora
Gerbera hybrid
Helianthus annuus
Helianthus paradoxus
Helianthus argophyllus
Helianthus exilis
Lactuca perennis
Lactuca sativa
Lactuca serriola
Lactuca virosa
Taraxacum officinale
Beta vulgaris
Arabidopsis thaliana
Brassica napus
Brassica oleracea
Brassica rapa
Gossypium arboreum
Gossypium hirsutum
Gossypium raimondii
Bruguiera gymnorrhiza
Euphorbia esula
Euphorbia tirucalli
Ricinus communis
Populus trichocarpa
Populus euphratica
Populus deltoides
Citrus clementina
Citrus sinensis
Glycine max
Phaseolus coccineus
Phaseolus vulgaris
Lotus japonicus
Medicago truncatula
Malus x domestica
Prunus persica
Cucumis sativus
Vitis vinifera
Aquilegia formosa x Aquilegia pubescens
Eschscholzia californica
Liriodendron tulipifera
Persea americana
Saruma henryi
Amborella trichopoda
Nuphar advena
Welwitschia mirabilis
Pinus pinaster
Pinus taeda
Picea sitchensis
Picea glauca
100 changes
13SCG ML

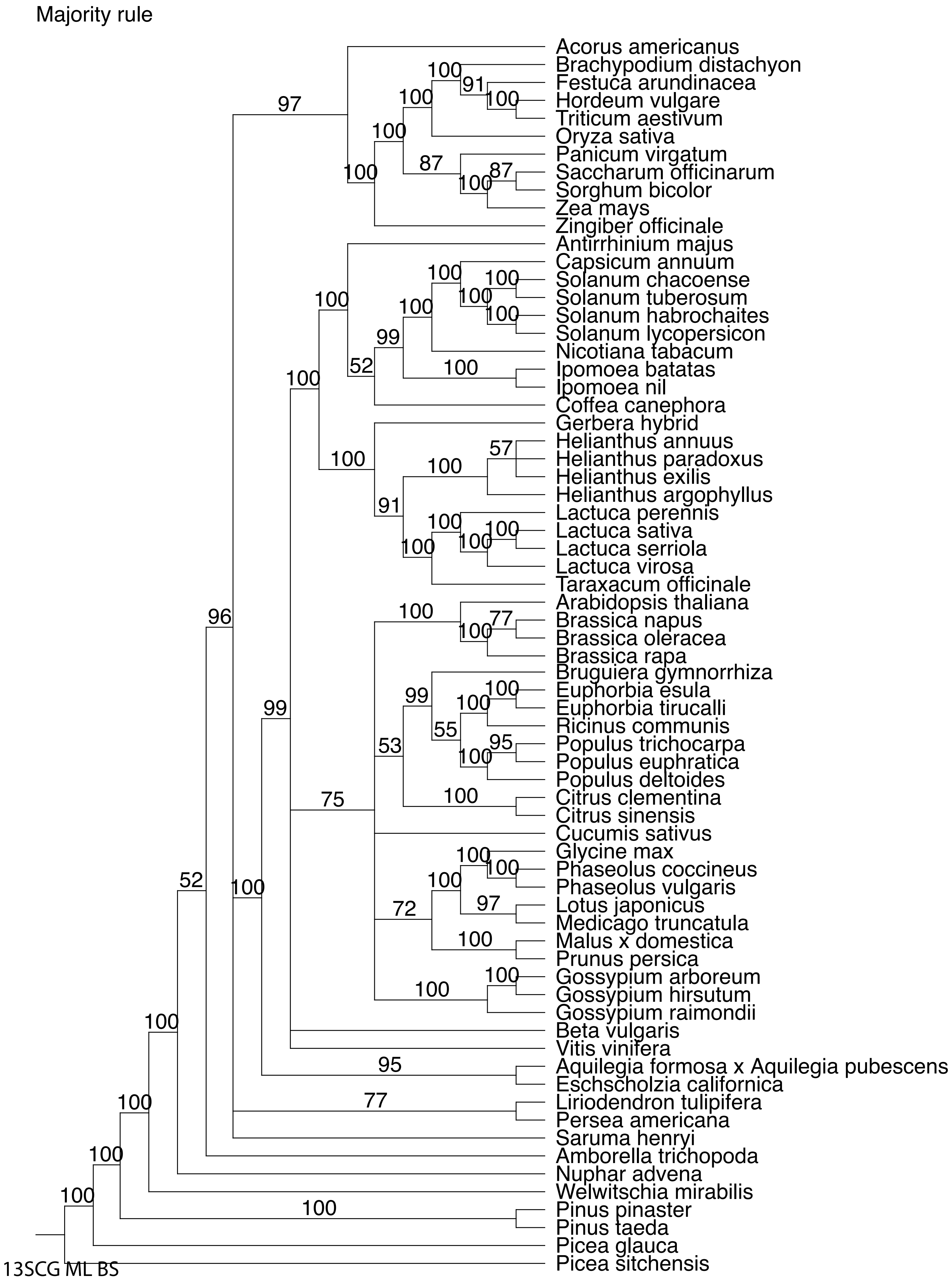
Majority rule
Acorus americanus
Brachypodium distachyon
Festuca arundinacea
Hordeum vulgare
Triticum aestivum
Oryza sativa
Panicum virgatum
Saccharum officinarum
Sorghum bicolor
Zea mays
Zingiber officinale
Antirrhinium majus
Capsicum annuum
Solanum chacoense
Solanum tuberosum
Solanum habrochaites
Solanum lycopersicon
Nicotiana tabacum
Ipomoea batatas
Ipomoea nil
Coffea canephora
Gerbera hybrid
Helianthus annuus
Helianthus paradoxus
Helianthus exilis
Helianthus argophyllus
Lactuca perennis
Lactuca sativa
Lactuca serriola
Lactuca virosa
Taraxacum officinale
Arabidopsis thaliana
Brassica napus
Brassica oleracea
Brassica rapa
Bruguiera gymnorrhiza
Euphorbia esula
Euphorbia tirucalli
Ricinus communis
Populus trichocarpa
Populus euphratica
Populus deltoides
Citrus clementina
Citrus sinensis
Cucumis sativus
Glycine max
Phaseolus coccineus
Phaseolus vulgaris
Lotus japonicus
Medicago truncatula
Malus x domestica
Prunus persica
Gossypium arboreum
Gossypium hirsutum
Gossypium raimondii
Beta vulgaris
Vitis vinifera
Aquilegia formosa x Aquilegia pubescens
Eschscholzia californica
Liriodendron tulipifera
Persea americana
Saruma henryi
Amborella trichopoda
Nuphar advena
Welwitschia mirabilis
Pinus pinaster
Pinus taeda
Picea glauca
Picea sitchensis
13SCG ML BS

Majority rule

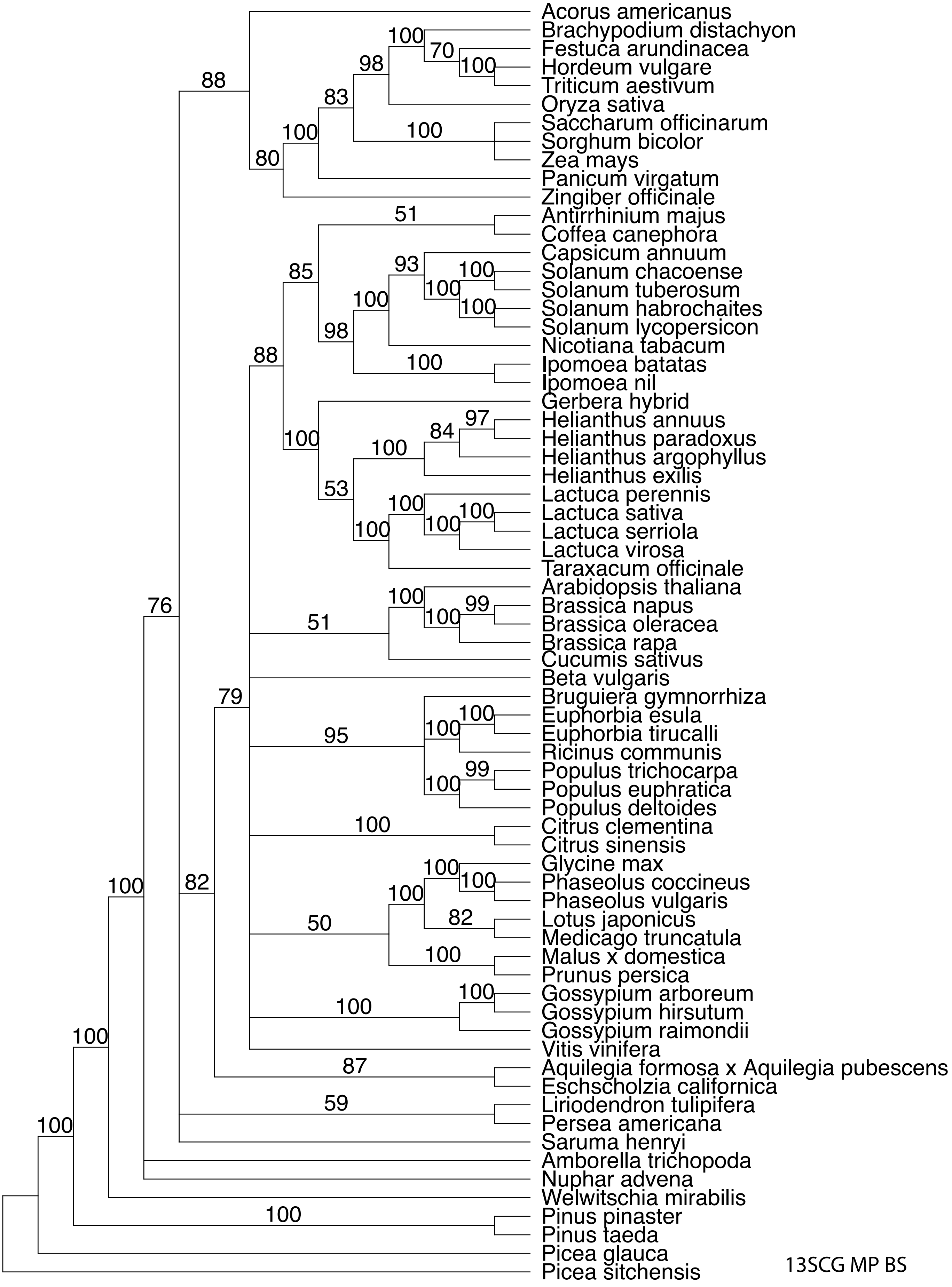

13SCG MP BS

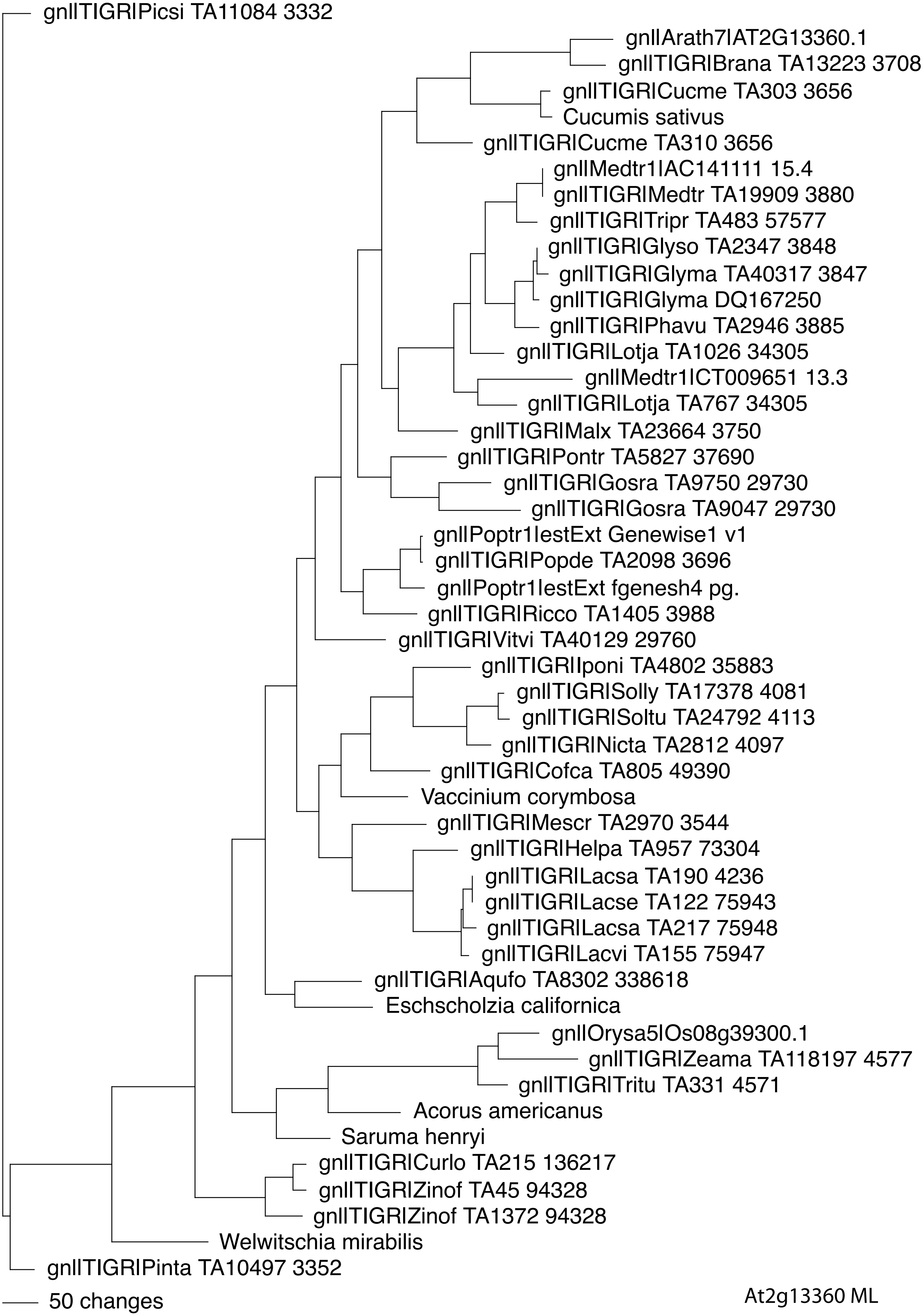

At2g13360 ML

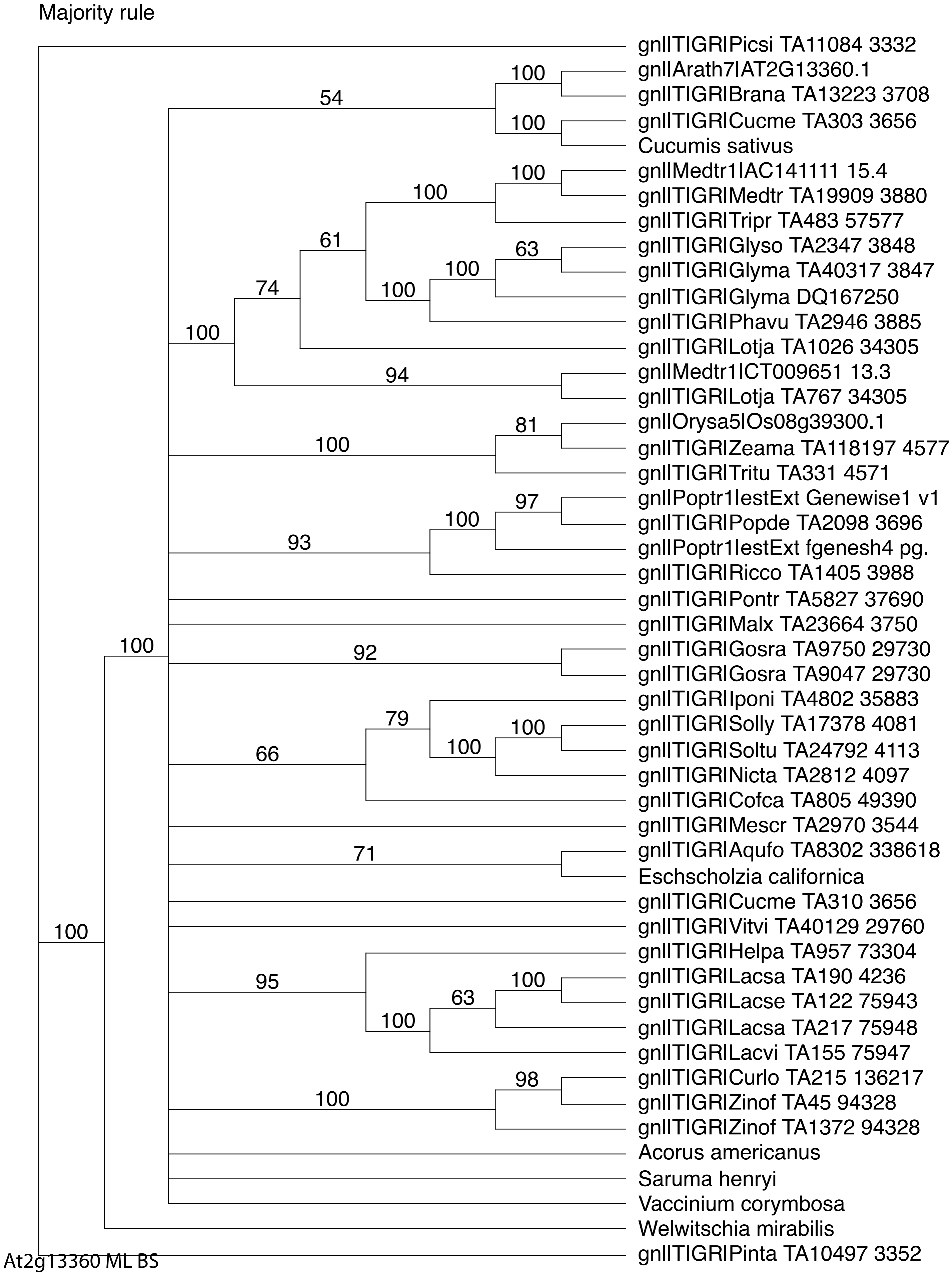
Majority rule
gnl|TIGR|Picsi TA11084 3332
gnl|Arath7|AT2G13360.1
gnl|TIGR|Brana TA13223 3708
gnl|TIGR|Cucme TA303 3656
Cucumis sativus
gnl|Medtr1|AC141111 15.4
gnl|TIGR|Medtr TA19909 3880
gnl|TIGR|Tripr TA483 57577
gnl|TIGR|Glyso TA2347 3848
gnl|TIGR|Glyma TA40317 3847
gnl|TIGR|Glyma DQ167250
gnl|TIGR|Phavu TA2946 3885
gnl|TIGR|Lotja TA1026 34305
gnl|Medtr1|CT009651 13.3
gnl|TIGR|Lotja TA767 34305
gnl|Orysa5|Os08g39300.1
gnl|TIGR|Zeama TA118197 4577
gnl|TIGR|Tritu TA331 4571
gnl|Poptr1|estExt Genewise1 v1
gnl|TIGR|Popde TA2098 3696
gnl|Poptr1|estExt fgenesh4 pg.
gnl|TIGR|Ricco TA1405 3988
gnl|TIGR|Pontr TA5827 37690
gnl|TIGR|Malx TA23664 3750
gnl|TIGR|Gosra TA9750 29730
gnl|TIGR|Gosra TA9047 29730
gnl|TIGR|Iponi TA4802 35883
gnl|TIGR|Solly TA17378 4081
gnl|TIGR|Soltu TA24792 4113
gnl|TIGR|Nicta TA2812 4097
gnl|TIGR|Cofca TA805 49390
gnl|TIGR|Mescr TA2970 3544
gnl|TIGR|Aqufo TA8302 338618
Eschscholzia californica
gnl|TIGR|Cucme TA310 3656
gnl|TIGR|Vitvi TA40129 29760
gnl|TIGR|Helpa TA957 73304
gnl|TIGR|Lacsa TA190 4236
gnl|TIGR|Lacse TA122 75943
gnl|TIGR|Lacsa TA217 75948
gnl|TIGR|Lacvi TA155 75947
gnl|TIGR|Curlo TA215 136217
gnl|TIGR|Zinof TA45 94328
gnl|TIGR|Zinof TA1372 94328
Acorus americanus
Saruma henryi
Vaccinium corymbosa
Welwitschia mirabilis
gnl|TIGR|Pinta TA10497 3352
At2g13360 ML BS

Majority rule

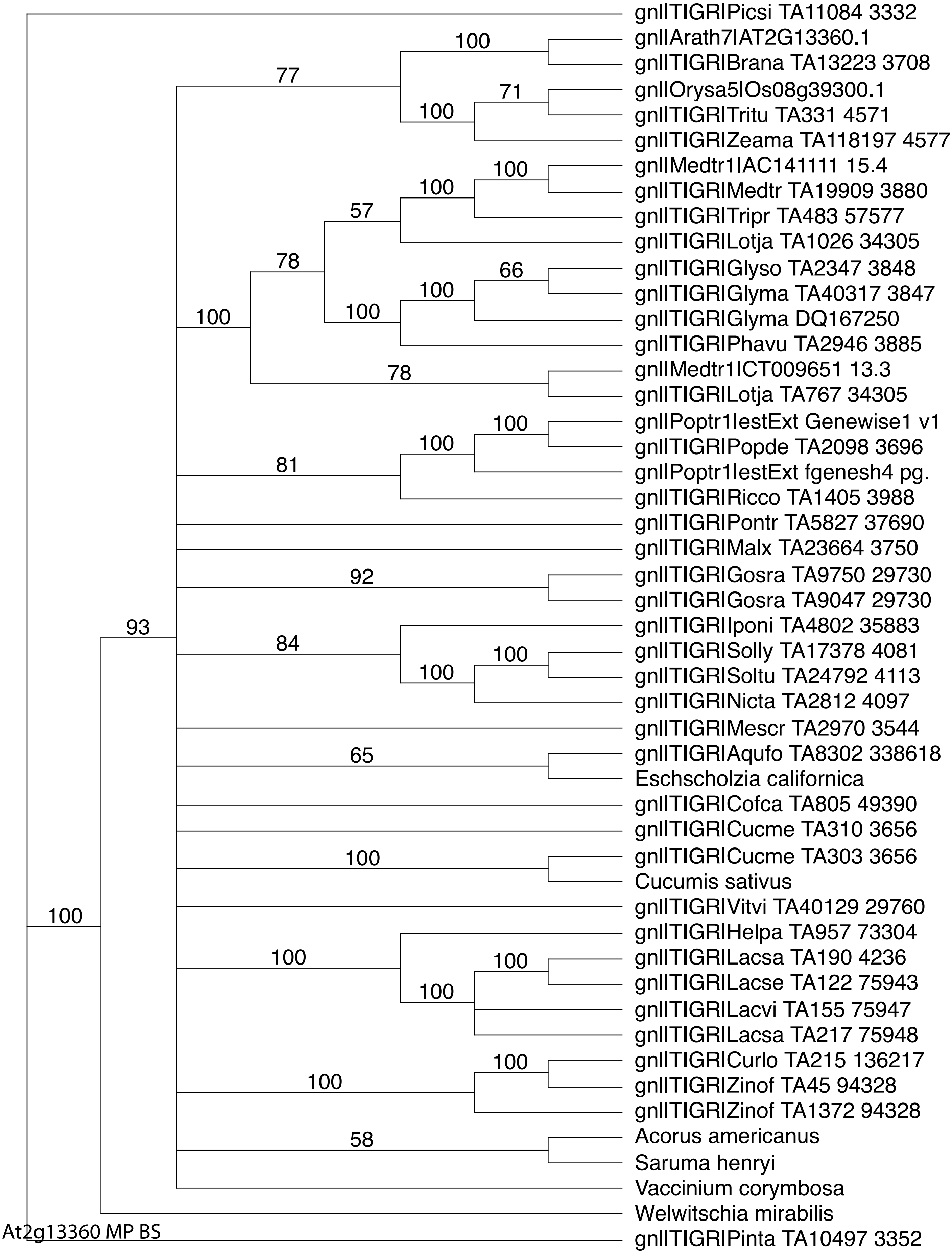

At2g13360 MP BS

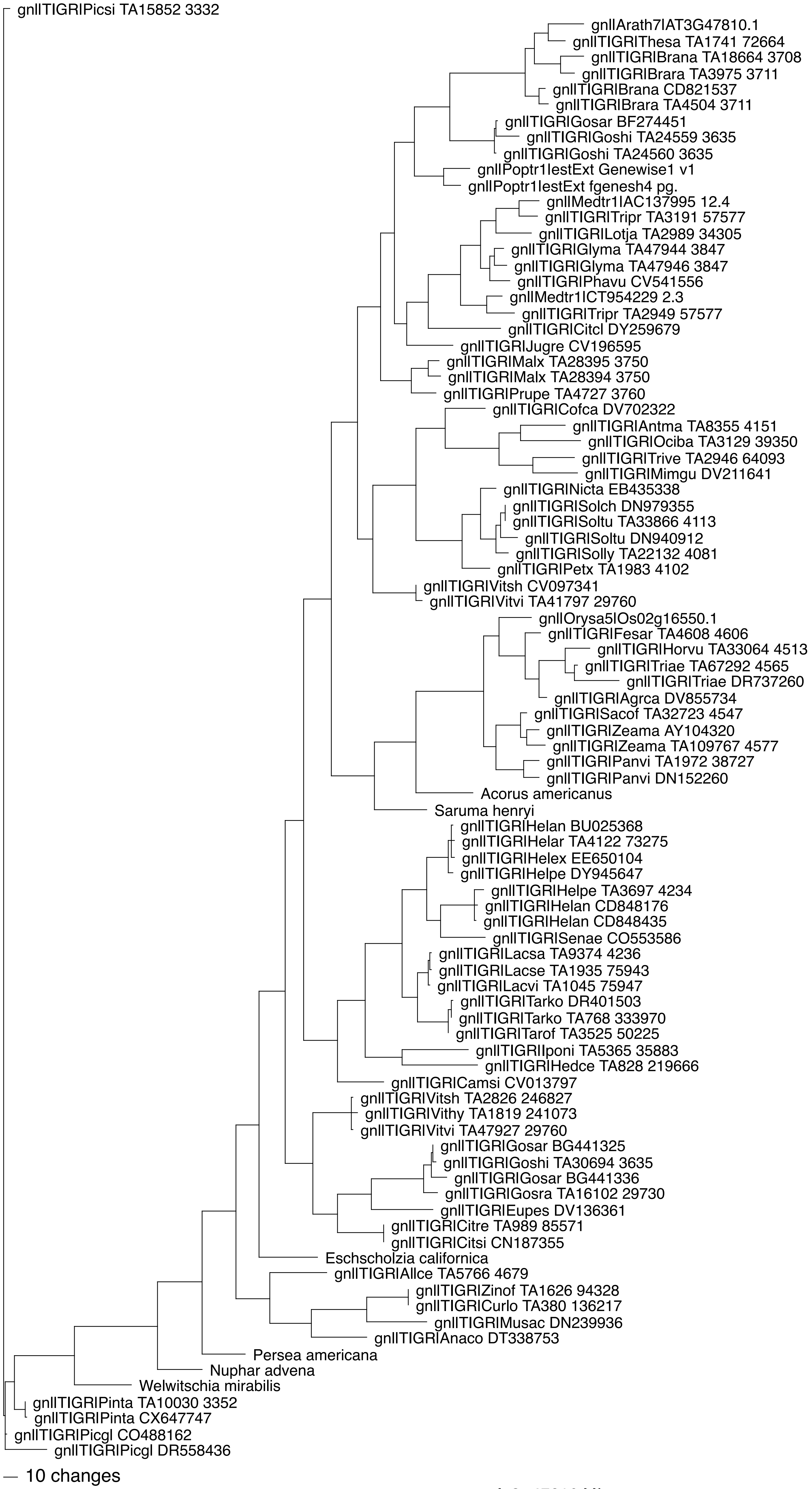

At3g47810 ML

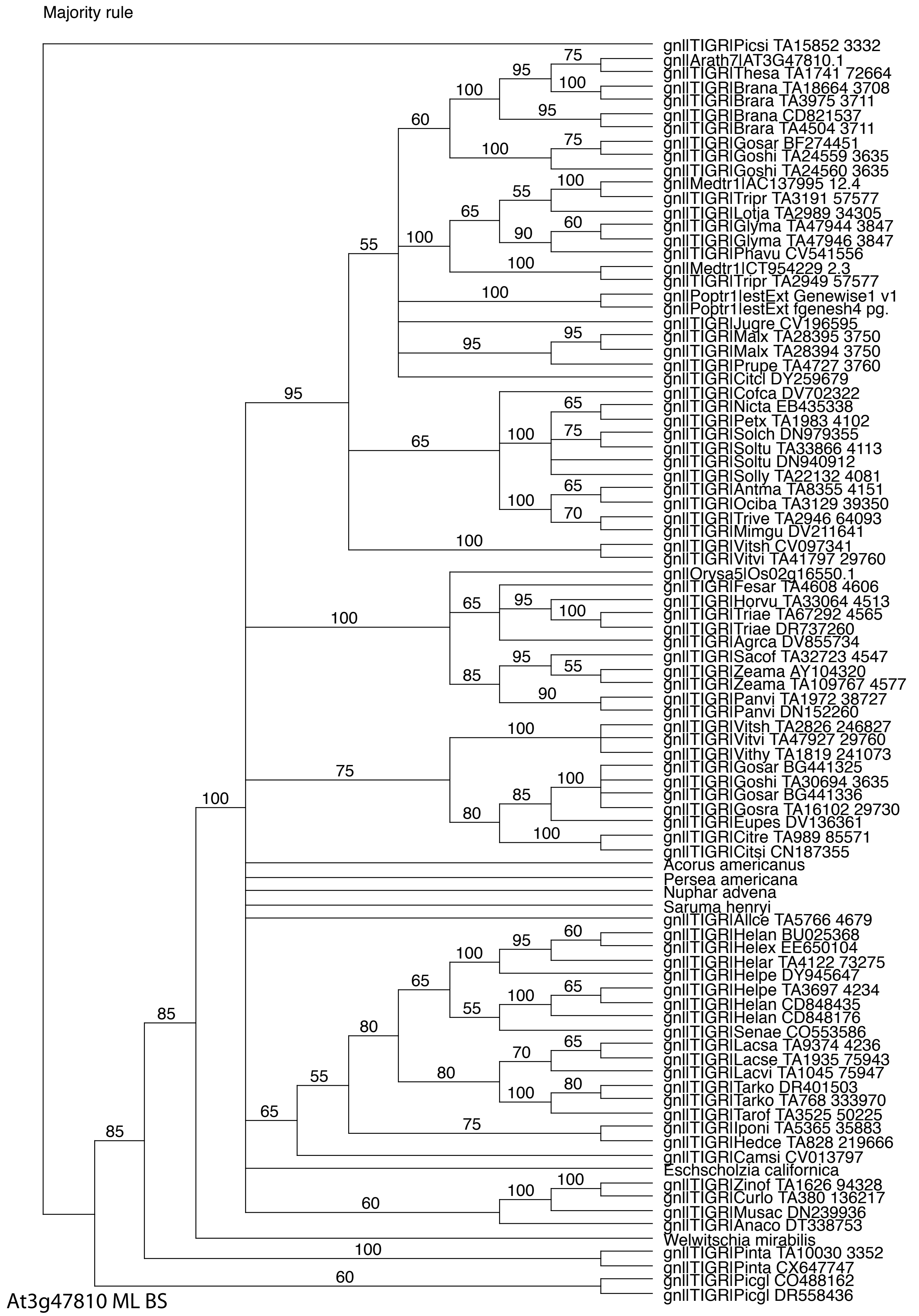
Majority rule
gnl|TIGR|Picsi TA15852 3332
gnl|Arath7|AT3G47810.1
gnl|TIGR|Thesa TA1741 72664
gnl|TIGR|Brana TA18664 3708
gnl|TIGR|Brara TA3975 3711
gnl|TIGR|Brana CD821537
gnl|TIGR|Brara TA4504 3711
gnl|TIGR|Gosar BF274451
gnl|TIGR|Goshi TA24559 3635
gnl|TIGR|Goshi TA24560 3635
gnl|Medtr1|AC137995 12.4
gnl|TIGR|Tripr TA3191 57577
gnl|TIGR|Lotja TA2989 34305
gnl|TIGR|Glyma TA47944 3847
gnl|TIGR|Glyma TA47946 3847
gnl|TIGR|Phavu CV541556
gnl|Medtr1|CT954229 2.3
gnl|TIGR|Tripr TA2949 57577
gnl|Poptr1|estExt Genewise1 v1
gnl|Poptr1|estExt fgenesh4 pg.
gnl|TIGR|Jugre CV196595
gnl|TIGR|Malx TA28395 3750
gnl|TIGR|Malx TA28394 3750
gnl|TIGR|Prupe TA4727 3760
gnl|TIGR|Citcl DY259679
gnl|TIGR|Cofca DV702322
gnl|TIGR|Nicta EB435338
gnl|TIGR|Petx TA1983 4102
gnl|TIGR|Solch DN979355
gnl|TIGR|Soltu TA33866 4113
gnl|TIGR|Soltu DN940912
gnl|TIGR|Solly TA22132 4081
gnl|TIGR|Antma TA8355 4151
gnl|TIGR|Ociba TA3129 39350
gnl|TIGR|Trive TA2946 64093
gnl|TIGR|Mimgu DV211641
gnl|TIGR|Vitsh CV097341
gnl|TIGR|Vitvi TA41797 29760
gnl|Orysa5|Os02g16550.1
gnl|TIGR|Fesar TA4608 4606
gnl|TIGR|Horvu TA33064 4513
gnl|TIGR|Triae TA67292 4565
gnl|TIGR|Triae DR737260
gnl|TIGR|Agrca DV855734
gnl|TIGR|Sacof TA32723 4547
gnl|TIGR|Zeama AY104320
gnl|TIGR|Zeama TA109767 4577
gnl|TIGR|Panvi TA1972 38727
gnl|TIGR|Panvi DN152260
gnl|TIGR|Vitsh TA2826 246827
gnl|TIGR|Vitvi TA47927 29760
gnl|TIGR|Vithy TA1819 241073
gnl|TIGR|Gosar BG441325
gnl|TIGR|Goshi TA30694 3635
gnl|TIGR|Gosar BG441336
gnl|TIGR|Gosra TA16102 29730
gnl|TIGR|Eupes DV136361
gnl|TIGR|Citre TA989 85571
gnl|TIGR|Citsi CN187355
Acorus americanus
Persea americana
Nuphar advena
Saruma henryi
gnl|TIGR|Allce TA5766 4679
gnl|TIGR|Helan BU025368
gnl|TIGR|Helex EE650104
gnl|TIGR|Helar TA4122 73275
gnl|TIGR|Helpe DY945647
gnl|TIGR|Helpe TA3697 4234
gnl|TIGR|Helan CD848435
gnl|TIGR|Helan CD848176
gnl|TIGR|Senae CO553586
gnl|TIGR|Lacsa TA9374 4236
gnl|TIGR|Lacse TA1935 75943
gnl|TIGR|Lacvi TA1045 75947
gnl|TIGR|Tarko DR401503
gnl|TIGR|Tarko TA768 333970
gnl|TIGR|Tarof TA3525 50225
gnl|TIGR|Iponi TA5365 35883
gnl|TIGR|Hedce TA828 219666
gnl|TIGR|Camsi CV013797
Eschscholzia californica
gnl|TIGR|Zinof TA1626 94328
gnl|TIGR|Curlo TA380 136217
gnl|TIGR|Musac DN239936
gnl|TIGR|Anaco DT338753
Welwitschia mirabilis
gnl|TIGR|Pinta TA10030 3352
gnl|TIGR|Pinta CX647747
gnl|TIGR|Picgl CO488162
gnl|TIGR|Picgl DR558436
At3g47810 ML BS

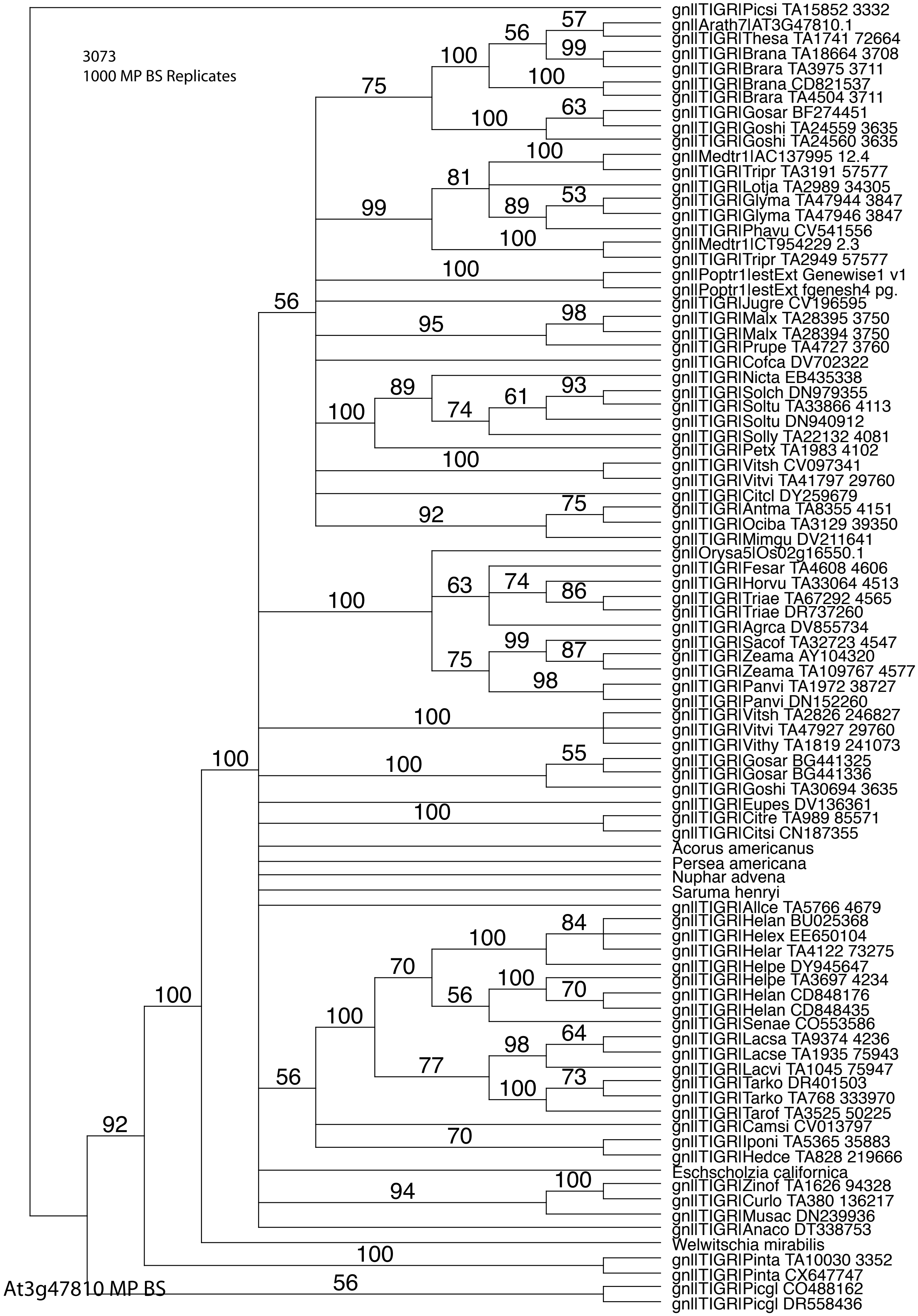
3073
1000 MP BS Replicates
At3g47810 MP BS
gnl|TIGR|Picsi TA15852 3332
gnl|Arath7|AT3G47810.1
gnl|TIGR|Thesa TA1741 72664
gnl|TIGR|Brana TA18664 3708
gnl|TIGR|Brara TA3975 3711
gnl|TIGR|Brana CD821537
gnl|TIGR|Brara TA4504 3711
gnl|TIGR|Gosar BF274451
gnl|TIGR|Goshi TA24559 3635
gnl|TIGR|Goshi TA24560 3635
gnl|Medtr1|AC137995 12.4
gnl|TIGR|Tripr TA3191 57577
gnl|TIGR|Lotja TA2989 34305
gnl|TIGR|Glyma TA47944 3847
gnl|TIGR|Glyma TA47946 3847
gnl|TIGR|Phavu CV541556
gnl|Medtr1|CT954229 2.3
gnl|TIGR|Tripr TA2949 57577
gnl|Poptr1|estExt Genewise1 v1
gnl|Poptr1|estExt fgenesh4 pg.
gnl|TIGR|Jugre CV196595
gnl|TIGR|Malx TA28395 3750
gnl|TIGR|Malx TA28394 3750
gnl|TIGR|Prupe TA4727 3760
gnl|TIGR|Cofca DV702322
gnl|TIGR|Nicta EB435338
gnl|TIGR|Solch DN979355
gnl|TIGR|Soltu TA33866 4113
gnl|TIGR|Soltu DN940912
gnl|TIGR|Solly TA22132 4081
gnl|TIGR|Petx TA1983 4102
gnl|TIGR|Vitsh CV097341
gnl|TIGR|Vitvi TA41797 29760
gnl|TIGR|Citcl DY259679
gnl|TIGR|Antma TA8355 4151
gnl|TIGR|Ociba TA3129 39350
gnl|TIGR|Mimgu DV211641
gnl|Orysa5|Os02g16550.1
gnl|TIGR|Fesar TA4608 4606
gnl|TIGR|Horvu TA33064 4513
gnl|TIGR|Triae TA67292 4565
gnl|TIGR|Triae DR737260
gnl|TIGR|Agrca DV855734
gnl|TIGR|Sacof TA32723 4547
gnl|TIGR|Zeama AY104320
gnl|TIGR|Zeama TA109767 4577
gnl|TIGR|Panvi TA1972 38727
gnl|TIGR|Panvi DN152260
gnl|TIGR|Vitsh TA2826 246827
gnl|TIGR|Vitvi TA47927 29760
gnl|TIGR|Vithy TA1819 241073
gnl|TIGR|Gosar BG441325
gnl|TIGR|Gosar BG441336
gnl|TIGR|Goshi TA30694 3635
gnl|TIGR|Eupes DV136361
gnl|TIGR|Citre TA989 85571
gnl|TIGR|Citsi CN187355
Acorus americanus
Persea americana
Nuphar advena
Saruma henryi
gnl|TIGR|Allce TA5766 4679
gnl|TIGR|Helan BU025368
gnl|TIGR|Helex EE650104
gnl|TIGR|Helar TA4122 73275
gnl|TIGR|Helpe DY945647
gnl|TIGR|Helpe TA3697 4234
gnl|TIGR|Helan CD848176
gnl|TIGR|Helan CD848435
gnl|TIGR|Senae CO553586
gnl|TIGR|Lacsa TA9374 4236
gnl|TIGR|Lacse TA1935 75943
gnl|TIGR|Lacvi TA1045 75947
gnl|TIGR|Tarko DR401503
gnl|TIGR|Tarko TA768 333970
gnl|TIGR|Tarof TA3525 50225
gnl|TIGR|Camsi CV013797
gnl|TIGR|Iponi TA5365 35883
gnl|TIGR|Hedce TA828 219666
Eschscholzia californica
gnl|TIGR|Zinof TA1626 94328
gnl|TIGR|Curlo TA380 136217
gnl|TIGR|Musac DN239936
gnl|TIGR|Anaco DT338753
Welwitschia mirabilis
gnl|TIGR|Pinta TA10030 3352
gnl|TIGR|Pinta CX647747
gnl|TIGR|Picgl CO488162
gnl|TIGR|Picgl DR558436

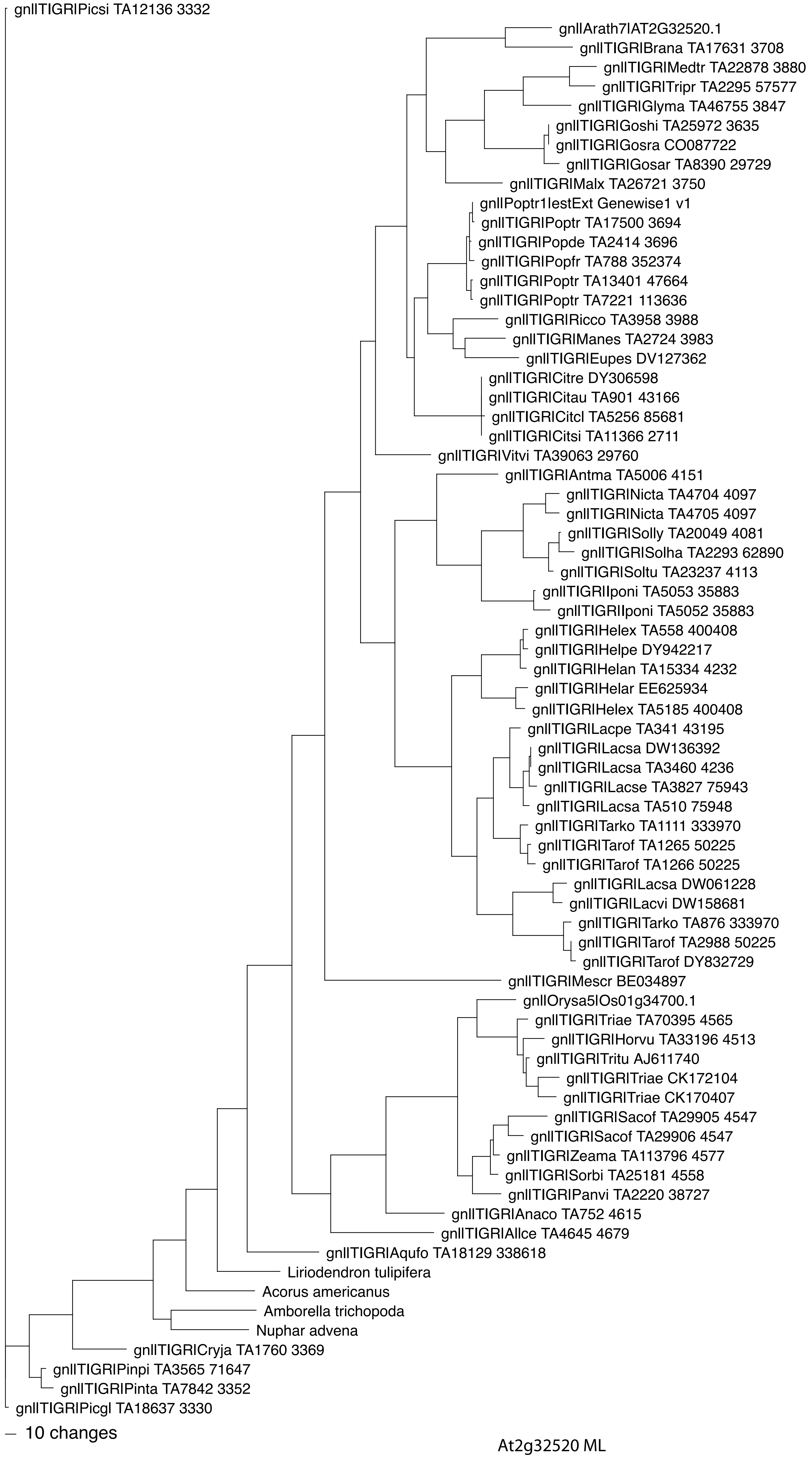

At2g32520 ML

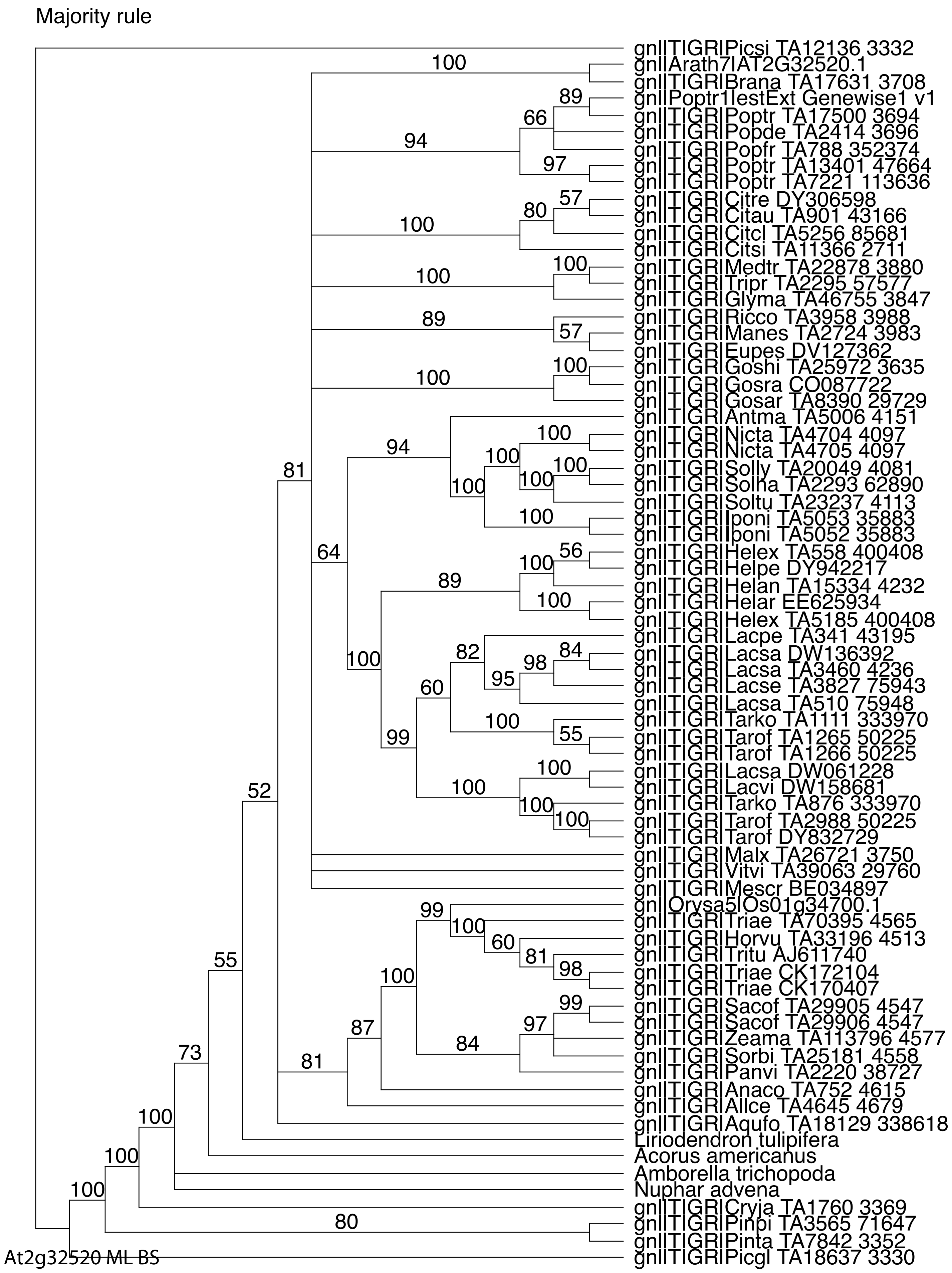
Majority rule
gnl|TIGR|Picsi TA12136 3332
gnl|Arath7|AT2G32520.1
gnl|TIGR|Brana TA17631 3708
gnl|Poptr1|estExt Genewise1 v1
gnl|TIGR|Poptr TA17500 3694
gnl|TIGR|Popde TA2414 3696
gnl|TIGR|Popfr TA788 352374
gnl|TIGR|Poptr TA13401 47664
gnl|TIGR|Poptr TA7221 113636
gnl|TIGR|Citre DY306598
gnl|TIGR|Citau TA901 43166
gnl|TIGR|Citcl TA5256 85681
gnl|TIGR|Citsi TA11366 2711
gnl|TIGR|Medtr TA22878 3880
gnl|TIGR|Tripr TA2295 57577
gnl|TIGR|Glyma TA46755 3847
gnl|TIGR|Ricco TA3958 3988
gnl|TIGR|Manes TA2724 3983
gnl|TIGR|Eupes DV127362
gnl|TIGR|Goshi TA25972 3635
gnl|TIGR|Gosra CO087722
gnl|TIGR|Gosar TA8390 29729
gnl|TIGR|Antma TA5006 4151
gnl|TIGR|Nicta TA4704 4097
gnl|TIGR|Nicta TA4705 4097
gnl|TIGR|Solly TA20049 4081
gnl|TIGR|Solha TA2293 62890
gnl|TIGR|Soltu TA23237 4113
gnl|TIGR|Iponi TA5053 35883
gnl|TIGR|Iponi TA5052 35883
gnl|TIGR|Helex TA558 400408
gnl|TIGR|Helpe DY942217
gnl|TIGR|Helan TA15334 4232
gnl|TIGR|Helar EE625934
gnl|TIGR|Helex TA5185 400408
gnl|TIGR|Lacpe TA341 43195
gnl|TIGR|Lacsa DW136392
gnl|TIGR|Lacsa TA3460 4236
gnl|TIGR|Lacse TA3827 75943
gnl|TIGR|Lacsa TA510 75948
gnl|TIGR|Tarko TA1111 333970
gnl|TIGR|Tarof TA1265 50225
gnl|TIGR|Tarof TA1266 50225
gnl|TIGR|Lacsa DW061228
gnl|TIGR|Lacvi DW158681
gnl|TIGR|Tarko TA876 333970
gnl|TIGR|Tarof TA2988 50225
gnl|TIGR|Tarof DY832729
gnl|TIGR|Malx TA26721 3750
gnl|TIGR|Vitvi TA39063 29760
gnl|TIGR|Mescr BE034897
gnl|Orysa5|Os01g34700.1
gnl|TIGR|Triae TA70395 4565
gnl|TIGR|Horvu TA33196 4513
gnl|TIGR|Tritu AJ611740
gnl|TIGR|Triae CK172104
gnl|TIGR|Triae CK170407
gnl|TIGR|Sacof TA29905 4547
gnl|TIGR|Sacof TA29906 4547
gnl|TIGR|Zeama TA113796 4577
gnl|TIGR|Sorbi TA25181 4558
gnl|TIGR|Panvi TA2220 38727
gnl|TIGR|Anaco TA752 4615
gnl|TIGR|Allce TA4645 4679
gnl|TIGR|Aqufo TA18129 338618
Liriodendron tulipifera
Acorus americanus
Amborella trichopoda
Nuphar advena
gnl|TIGR|Cryja TA1760 3369
gnl|TIGR|Pinpi TA3565 71647
gnl|TIGR|Pinta TA7842 3352
gnl|TIGR|Picgl TA18637 3330
At2g32520 ML BS

Majority rule

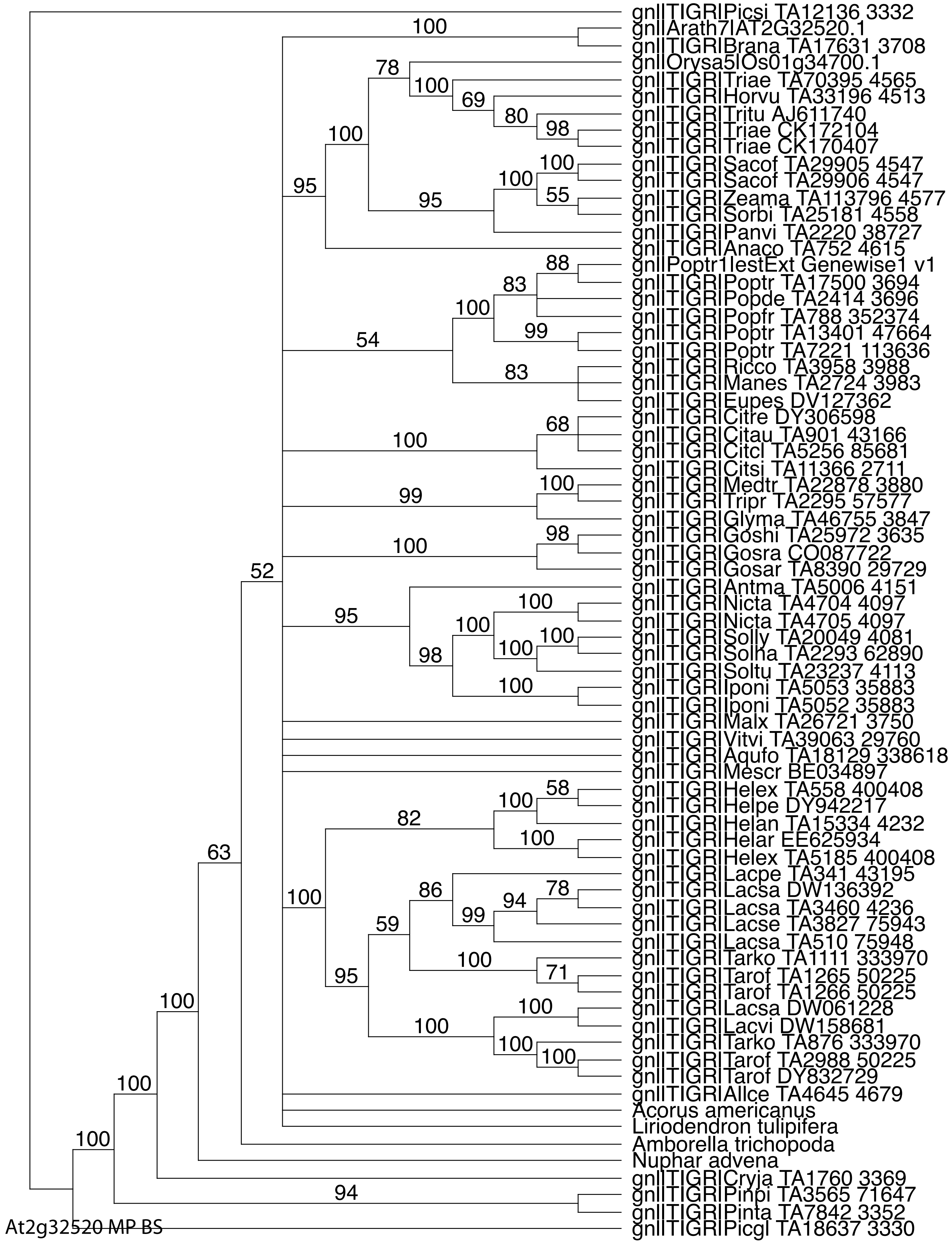

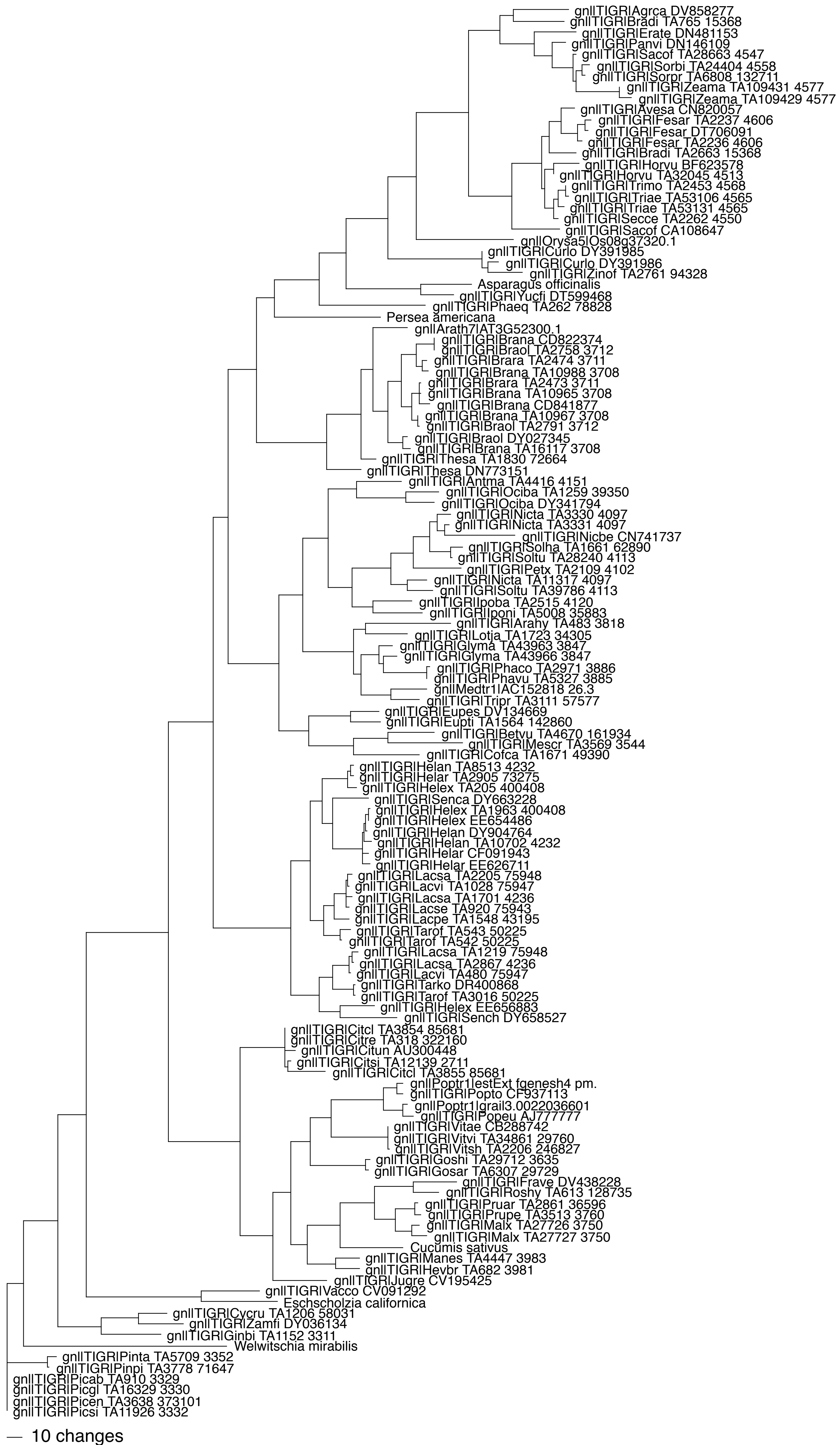

At3g52300 ML

Majority rule

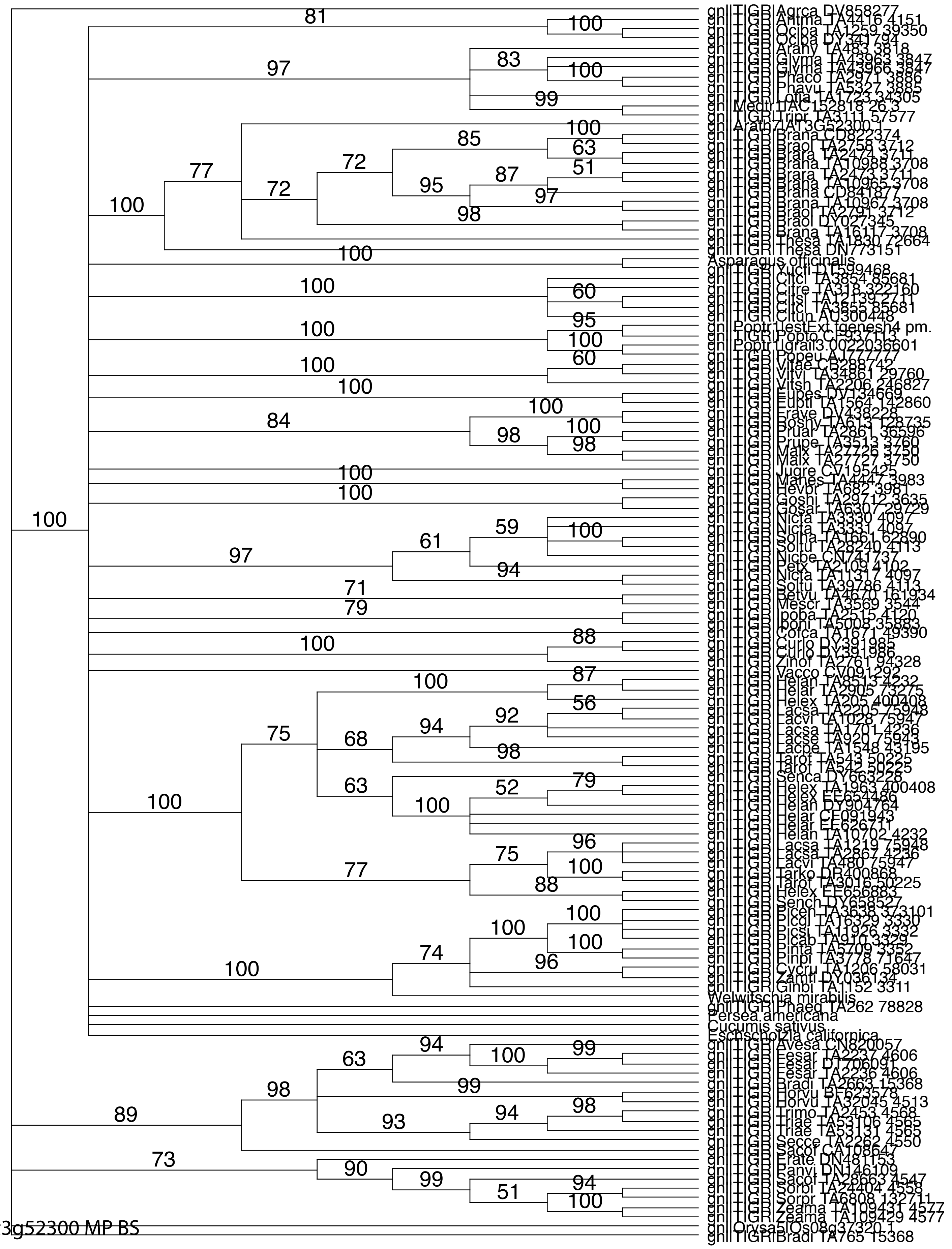

At3g52300 MP BS

Majority rule

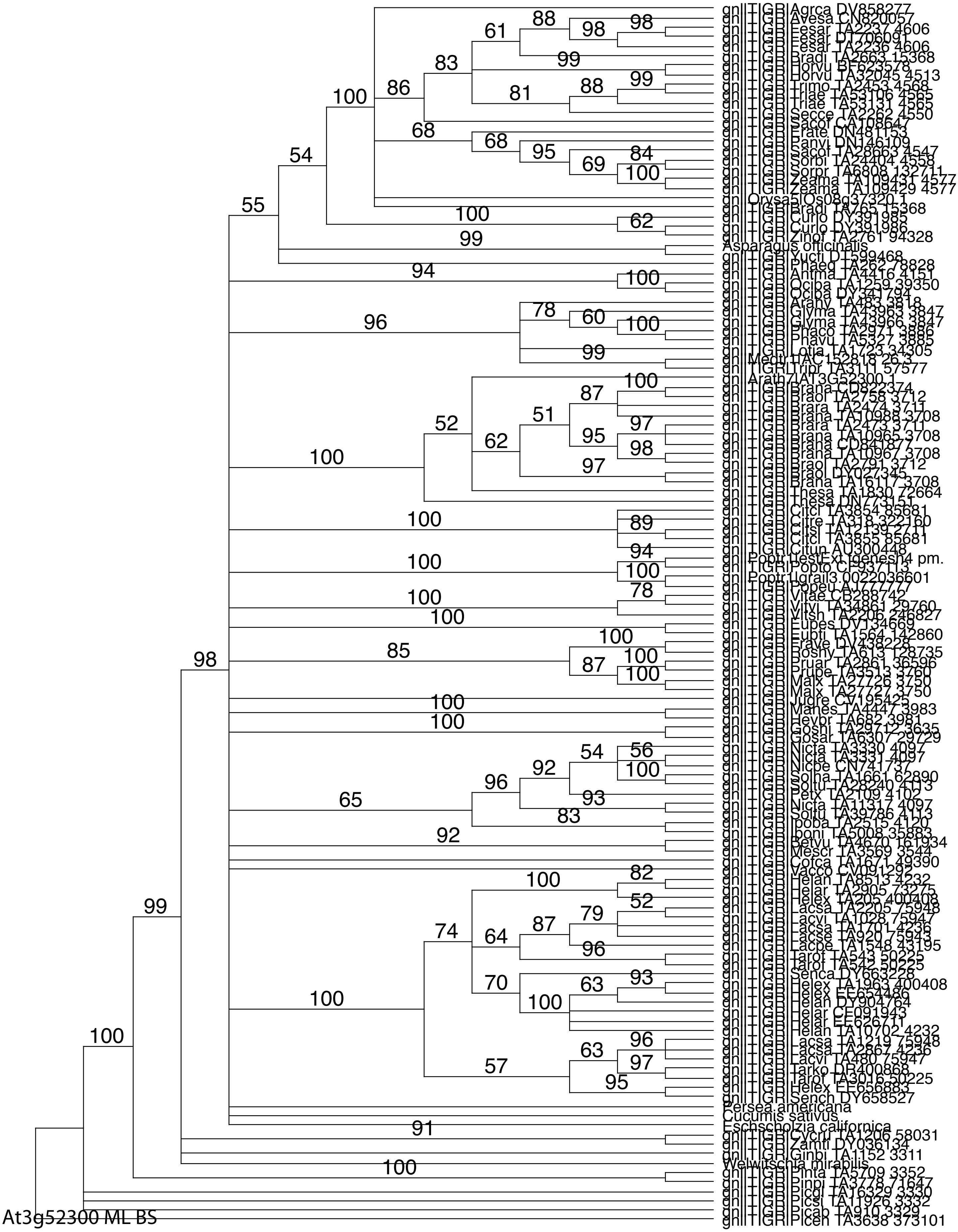

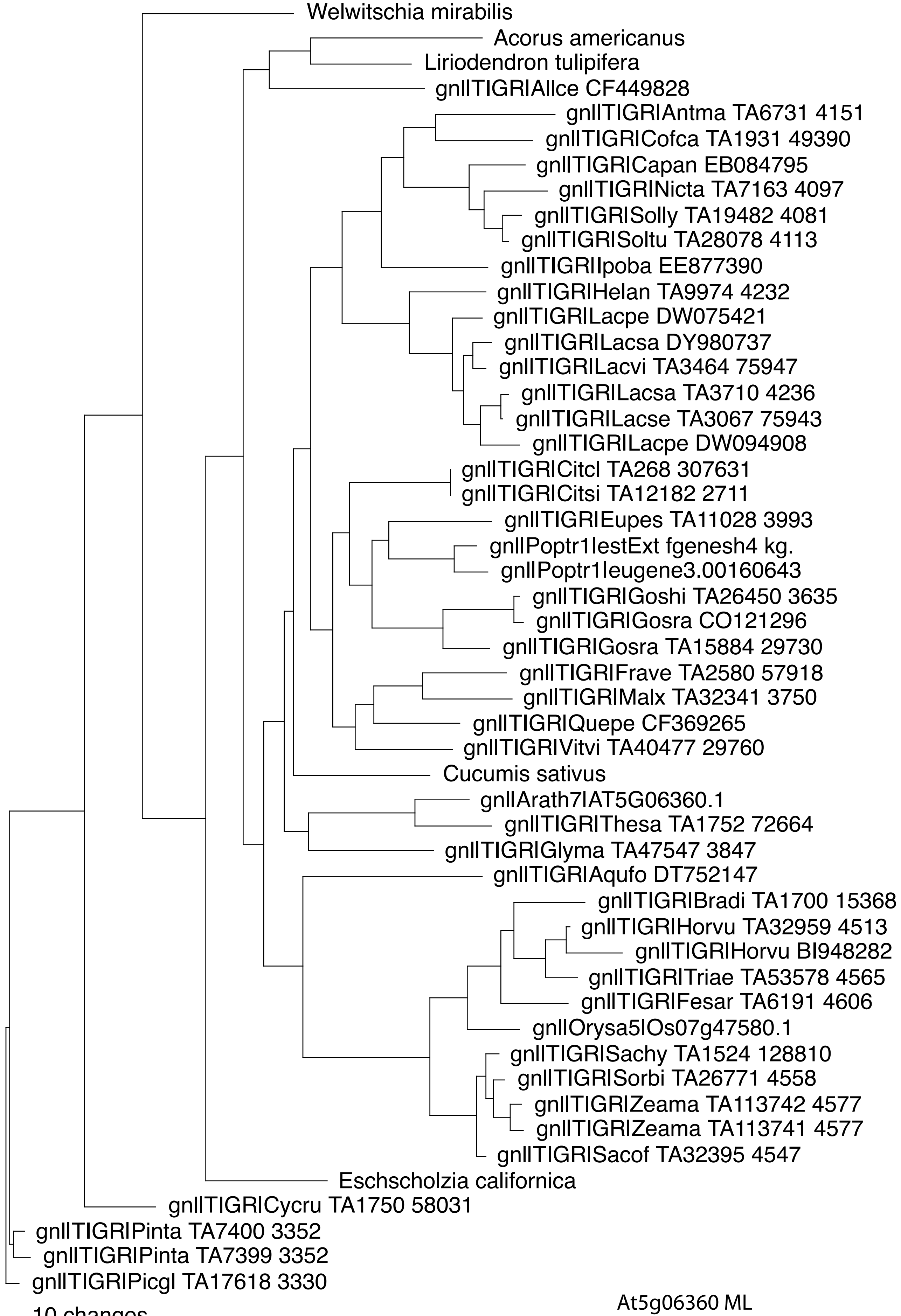
Welwitschia mirabilis
Acorus americanus
Liriodendron tulipifera
gnl|TIGR|Allce CF449828
gnl|TIGR|Antma TA6731 4151
gnl|TIGR|Cofca TA1931 49390
gnl|TIGR|Capan EB084795
gnl|TIGR|Nicta TA7163 4097
gnl|TIGR|Solly TA19482 4081
gnl|TIGR|Soltu TA28078 4113
gnl|TIGR|Ipoba EE877390
gnl|TIGR|Helan TA9974 4232
gnl|TIGR|Lacpe DW075421
gnl|TIGR|Lacsa DY980737
gnl|TIGR|Lacvi TA3464 75947
gnl|TIGR|Lacsa TA3710 4236
gnl|TIGR|Lacse TA3067 75943
gnl|TIGR|Lacpe DW094908
gnl|TIGR|Citcl TA268 307631
gnl|TIGR|Citsi TA12182 2711
gnl|TIGR|Eupes TA11028 3993
gnl|Poptr1|estExt fgenesh4 kg.
gnl|Poptr1|eugene3.00160643
gnl|TIGR|Goshi TA26450 3635
gnl|TIGR|Gosra CO121296
gnl|TIGR|Gosra TA15884 29730
gnl|TIGR|Frave TA2580 57918
gnl|TIGR|Malx TA32341 3750
gnl|TIGR|Quepe CF369265
gnl|TIGR|Vitvi TA40477 29760
Cucumis sativus
gnl|Arath7|AT5G06360.1
gnl|TIGR|Thesa TA1752 72664
gnl|TIGR|Glyma TA47547 3847
gnl|TIGR|Aqufo DT752147
gnl|TIGR|Bradi TA1700 15368
gnl|TIGR|Horvu TA32959 4513
gnl|TIGR|Horvu BI948282
gnl|TIGR|Triae TA53578 4565
gnl|TIGR|Fesar TA6191 4606
gnl|Orysa5|Os07g47580.1
gnl|TIGR|Sachy TA1524 128810
gnl|TIGR|Sorbi TA26771 4558
gnl|TIGR|Zeama TA113742 4577
gnl|TIGR|Zeama TA113741 4577
gnl|TIGR|Sacof TA32395 4547
Eschscholzia californica
gnl|TIGR|Cycru TA1750 58031
gnl|TIGR|Pinta TA7400 3352
gnl|TIGR|Pinta TA7399 3352
gnl|TIGR|Picgl TA17618 3330
10 changes
At5g06360 ML

Majority rule

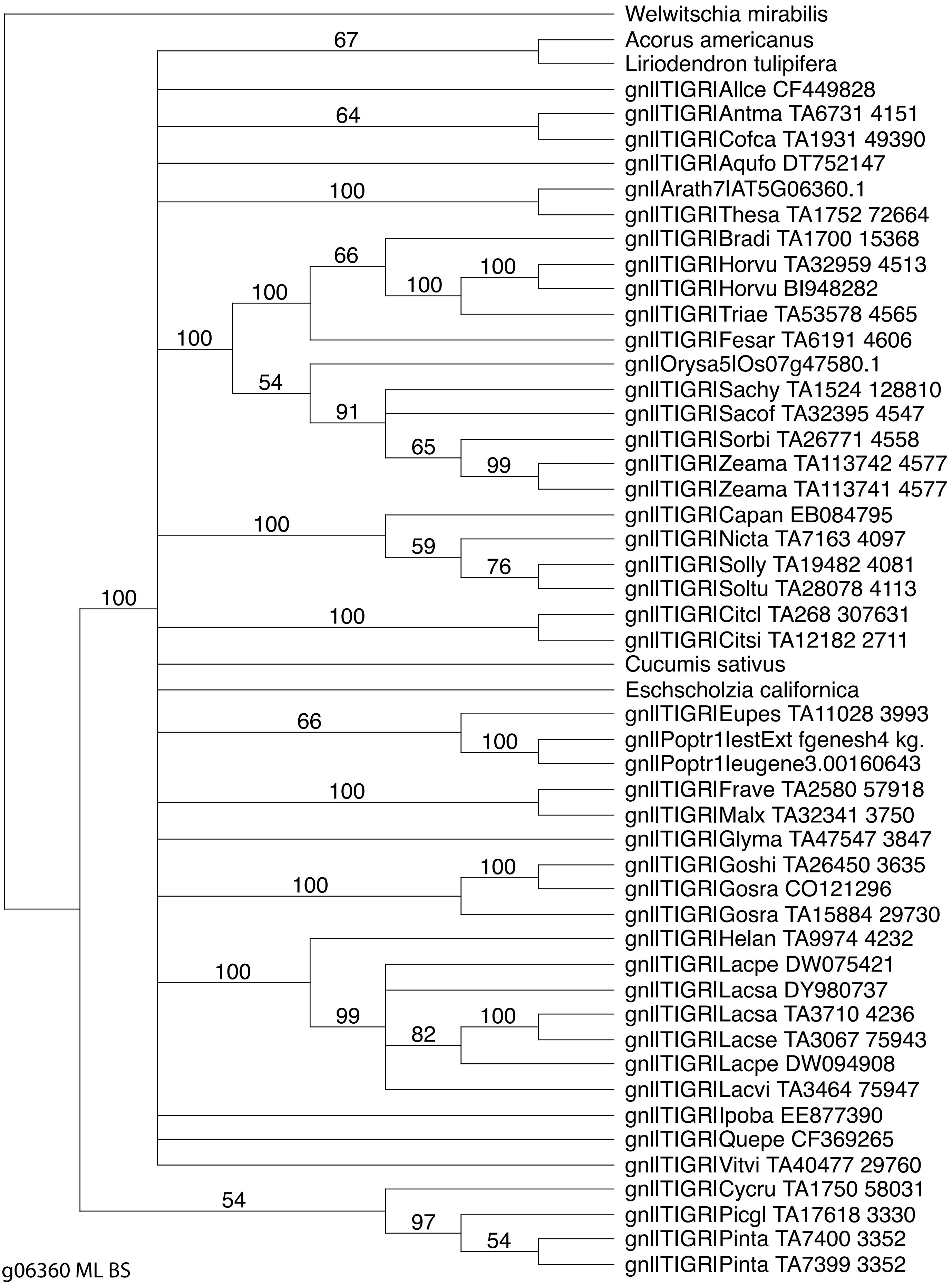

At5g06360 ML BS

Majority rule

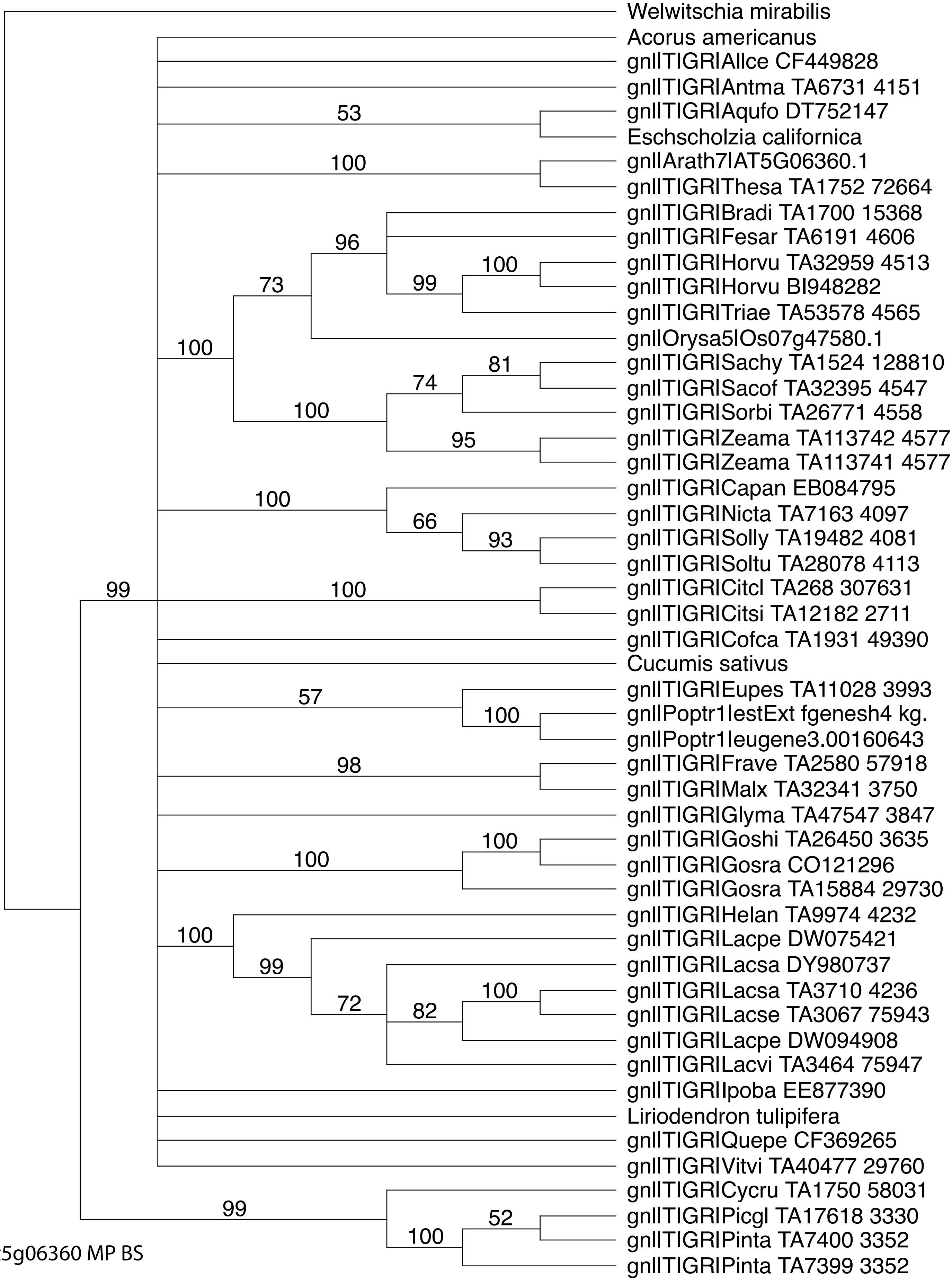

At5g06360 MP BS

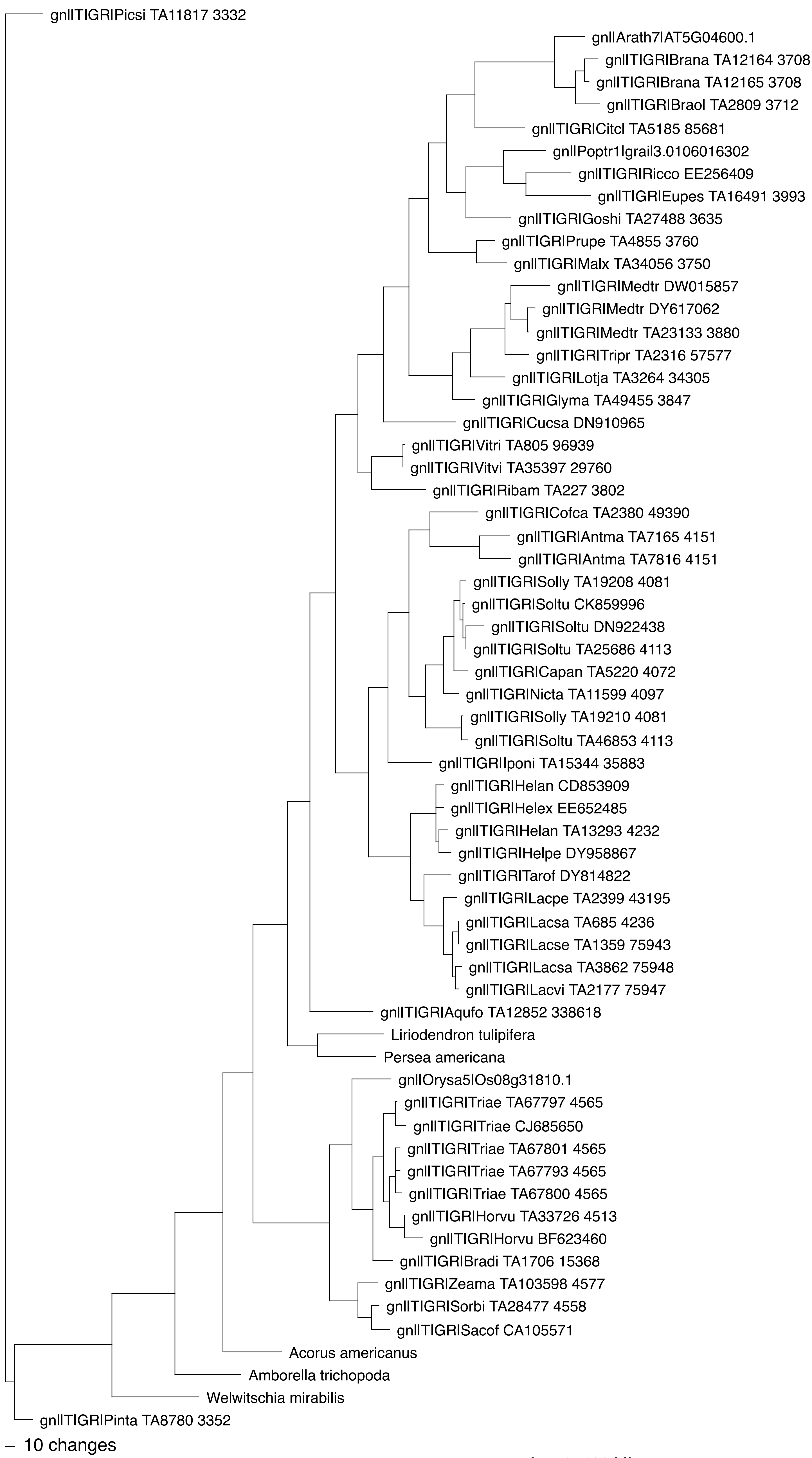

At5g04600 ML

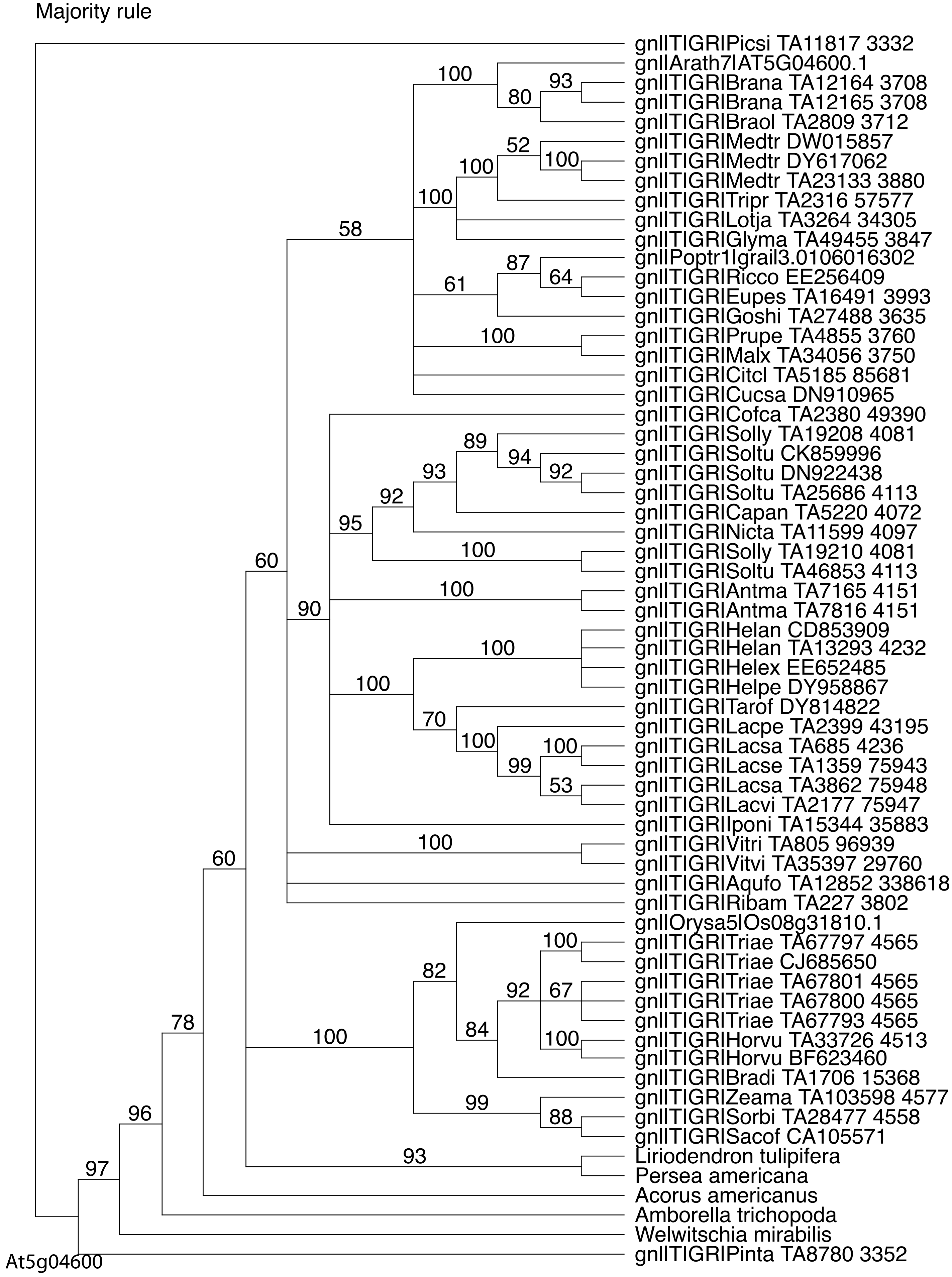
Majority rule
gnl|TIGR|Picsi TA11817 3332
gnl|Arath7|AT5G04600.1
gnl|TIGR|Brana TA12164 3708
gnl|TIGR|Brana TA12165 3708
gnl|TIGR|Braol TA2809 3712
gnl|TIGR|Medtr DW015857
gnl|TIGR|Medtr DY617062
gnl|TIGR|Medtr TA23133 3880
gnl|TIGR|Tripr TA2316 57577
gnl|TIGR|Lotja TA3264 34305
gnl|TIGR|Glyma TA49455 3847
gnl|Poptr1|grail3.0106016302
gnl|TIGR|Ricco EE256409
gnl|TIGR|Eupes TA16491 3993
gnl|TIGR|Goshi TA27488 3635
gnl|TIGR|Prupe TA4855 3760
gnl|TIGR|Malx TA34056 3750
gnl|TIGR|Citcl TA5185 85681
gnl|TIGR|Cucsa DN910965
gnl|TIGR|Cofca TA2380 49390
gnl|TIGR|Solly TA19208 4081
gnl|TIGR|Soltu CK859996
gnl|TIGR|Soltu DN922438
gnl|TIGR|Soltu TA25686 4113
gnl|TIGR|Capan TA5220 4072
gnl|TIGR|Nicta TA11599 4097
gnl|TIGR|Solly TA19210 4081
gnl|TIGR|Soltu TA46853 4113
gnl|TIGR|Antma TA7165 4151
gnl|TIGR|Antma TA7816 4151
gnl|TIGR|Helan CD853909
gnl|TIGR|Helan TA13293 4232
gnl|TIGR|Helex EE652485
gnl|TIGR|Helpe DY958867
gnl|TIGR|Tarof DY814822
gnl|TIGR|Lacpe TA2399 43195
gnl|TIGR|Lacsa TA685 4236
gnl|TIGR|Lacse TA1359 75943
gnl|TIGR|Lacsa TA3862 75948
gnl|TIGR|Lacvi TA2177 75947
gnl|TIGR|Iponi TA15344 35883
gnl|TIGR|Vitri TA805 96939
gnl|TIGR|Vitvi TA35397 29760
gnl|TIGR|Aqufo TA12852 338618
gnl|TIGR|Ribam TA227 3802
gnl|Orysa5|Os08g31810.1
gnl|TIGR|Triae TA67797 4565
gnl|TIGR|Triae CJ685650
gnl|TIGR|Triae TA67801 4565
gnl|TIGR|Triae TA67800 4565
gnl|TIGR|Triae TA67793 4565
gnl|TIGR|Horvu TA33726 4513
gnl|TIGR|Horvu BF623460
gnl|TIGR|Bradi TA1706 15368
gnl|TIGR|Zeama TA103598 4577
gnl|TIGR|Sorbi TA28477 4558
gnl|TIGR|Sacof CA105571
Liriodendron tulipifera
Persea americana
Acorus americanus
Amborella trichopoda
Welwitschia mirabilis
gnl|TIGR|Pinta TA8780 3352
At5g04600

Majority rule

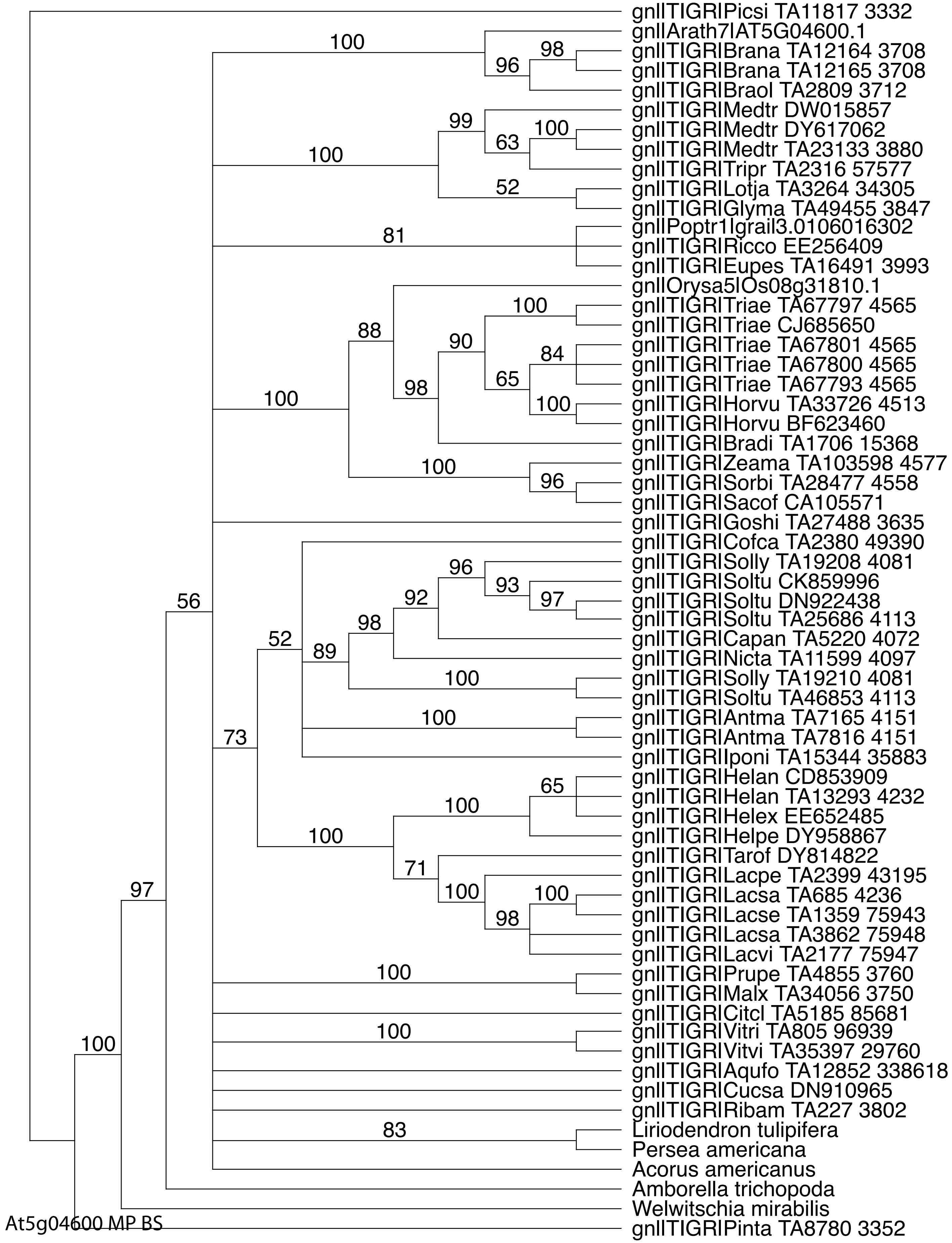

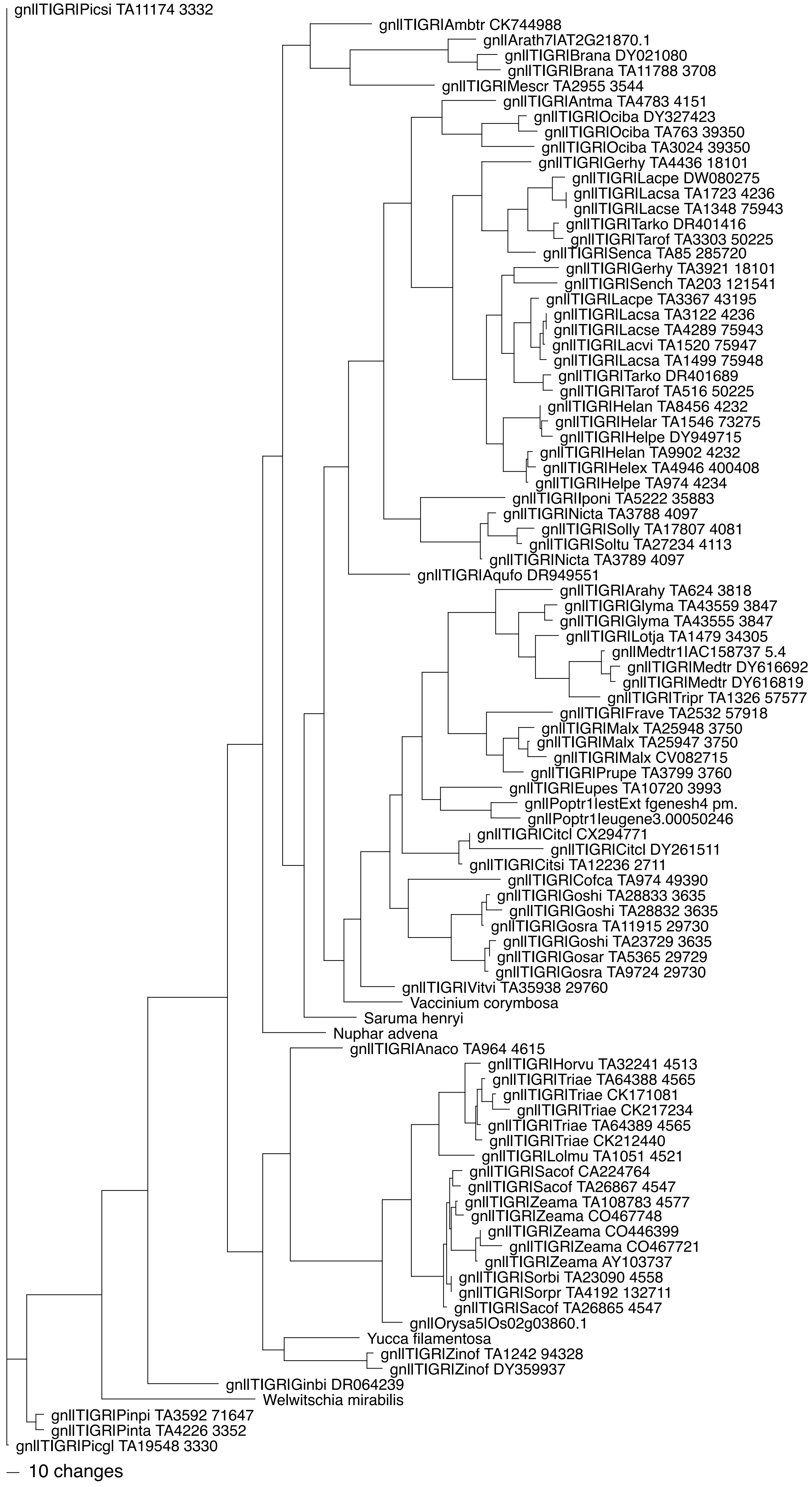

At2g21870 ML

Majority rule

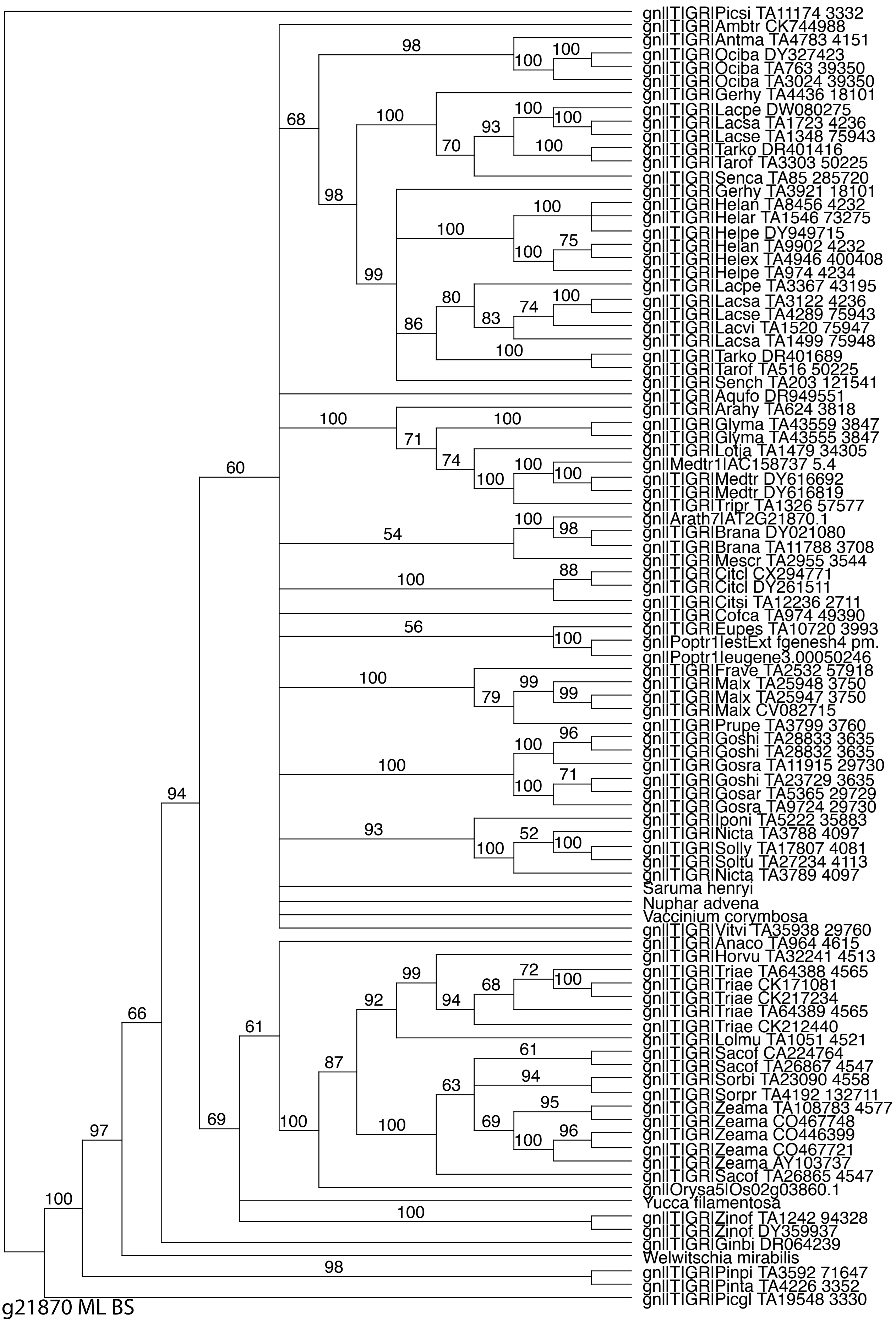

At2g21870 ML BS

Majority rule

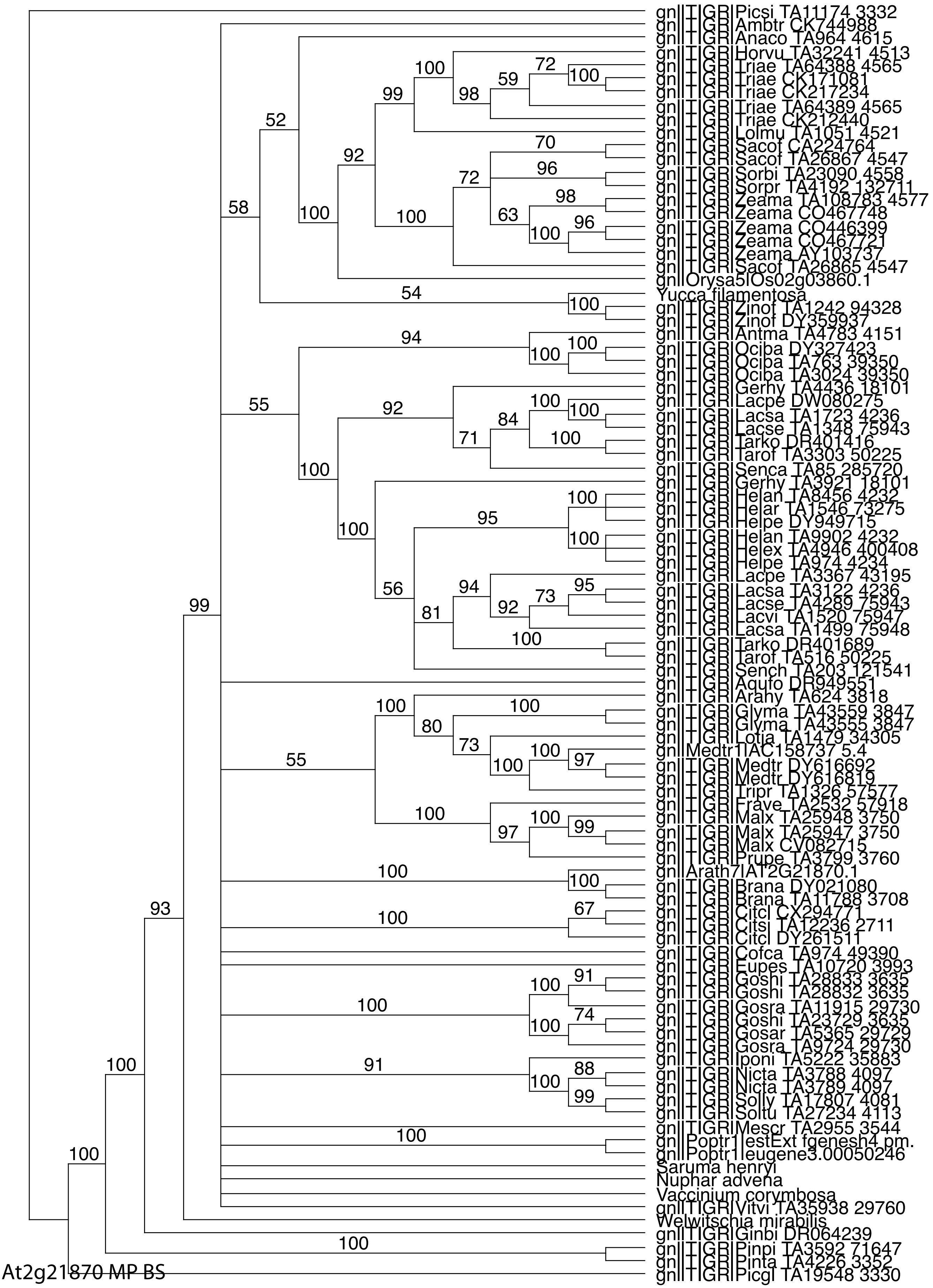

At2g21870 MP BS

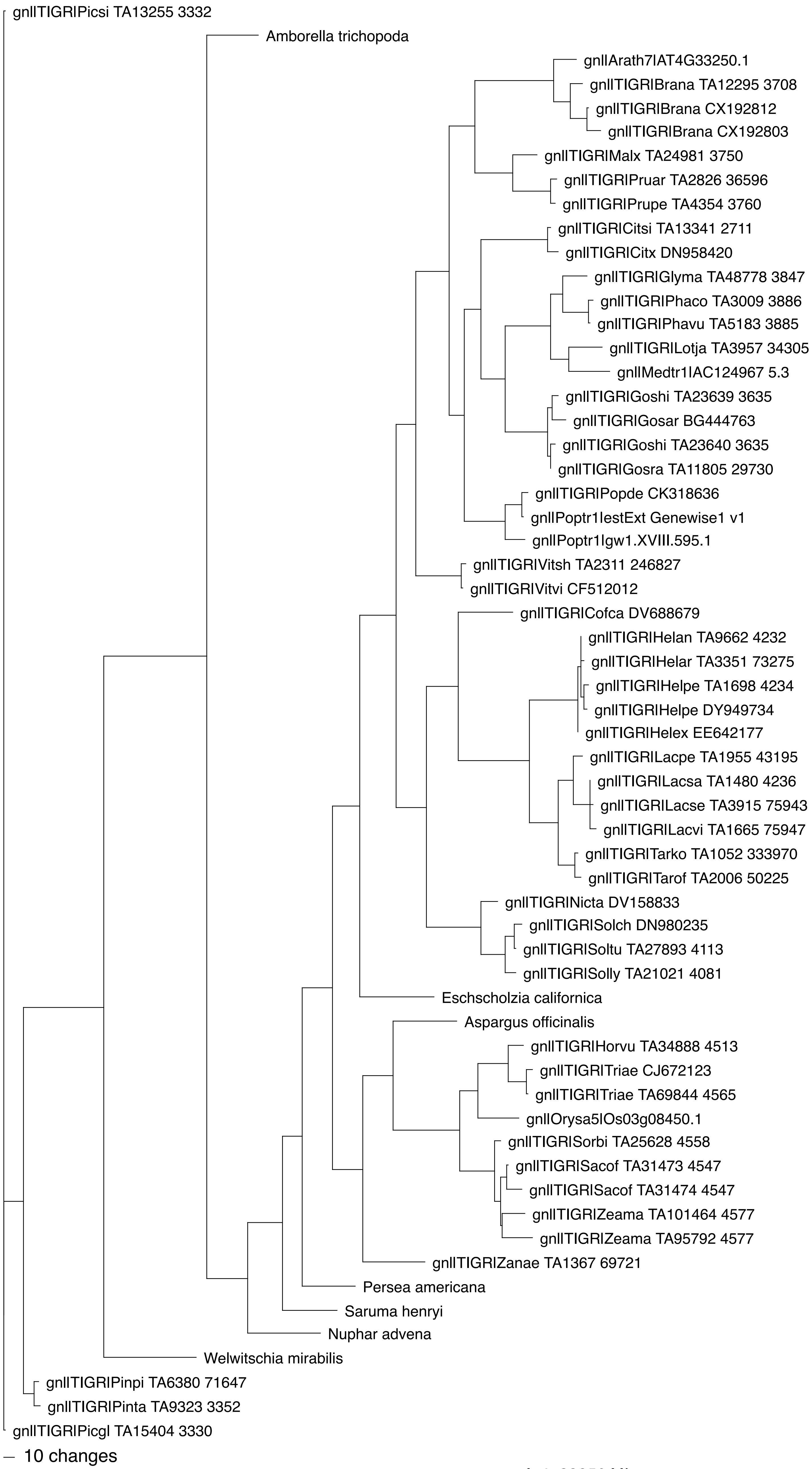

At4g33250 ML

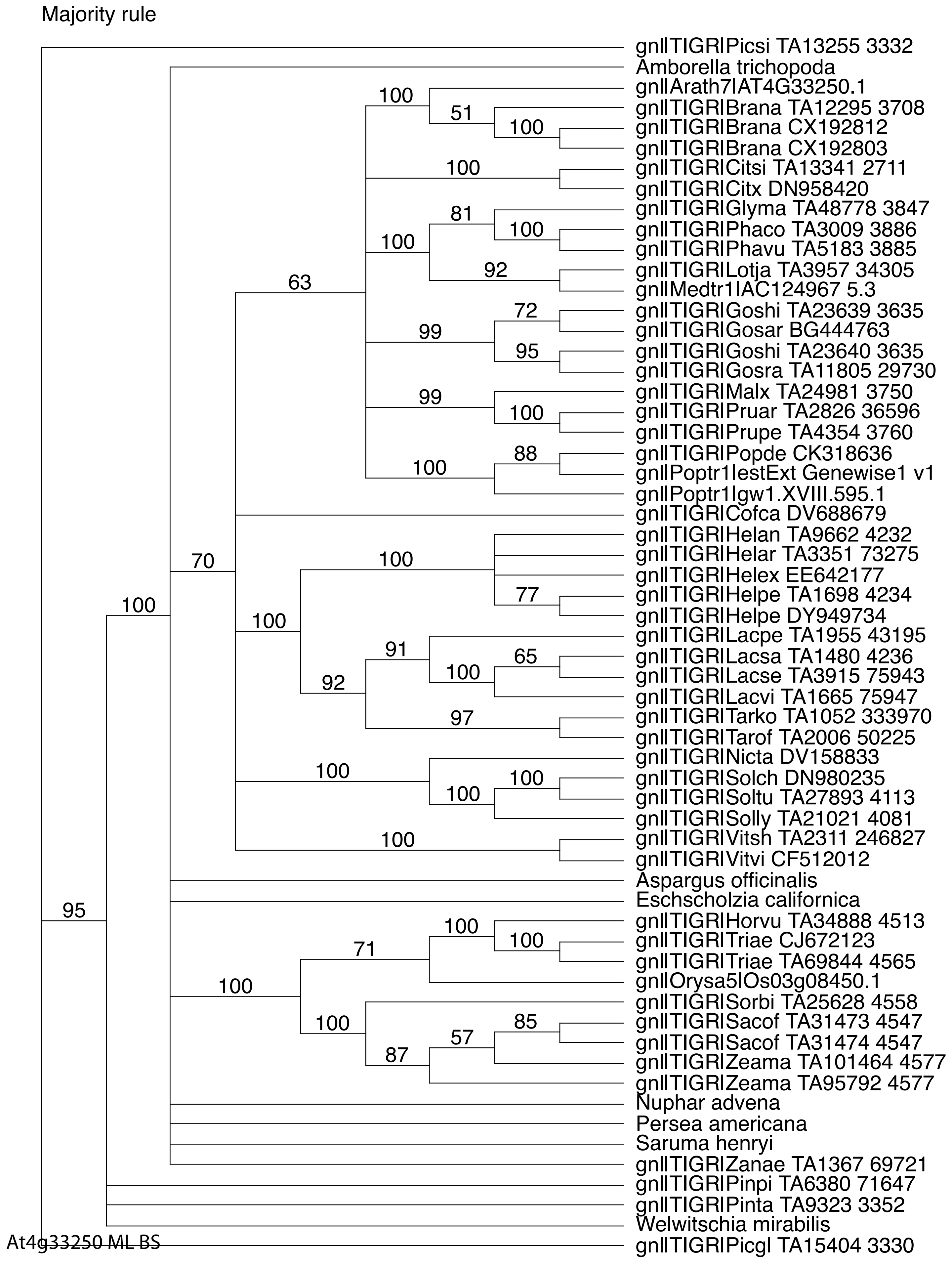
Majority rule
gnl|TIGR|Picsi TA13255 3332
Amborella trichopoda
gnl|Arath7|AT4G33250.1
gnl|TIGR|Brana TA12295 3708
gnl|TIGR|Brana CX192812
gnl|TIGR|Brana CX192803
gnl|TIGR|Citsi TA13341 2711
gnl|TIGR|Citx DN958420
gnl|TIGR|Glyma TA48778 3847
gnl|TIGR|Phaco TA3009 3886
gnl|TIGR|Phavu TA5183 3885
gnl|TIGR|Lotja TA3957 34305
gnl|Medtr1|AC124967 5.3
gnl|TIGR|Goshi TA23639 3635
gnl|TIGR|Gosar BG444763
gnl|TIGR|Goshi TA23640 3635
gnl|TIGR|Gosra TA11805 29730
gnl|TIGR|Malx TA24981 3750
gnl|TIGR|Pruar TA2826 36596
gnl|TIGR|Prupe TA4354 3760
gnl|TIGR|Popde CK318636
gnl|Poptr1|estExt Genewise1 v1
gnl|Poptr1|gw1.XVIII.595.1
gnl|TIGR|Cofca DV688679
gnl|TIGR|Helan TA9662 4232
gnl|TIGR|Helar TA3351 73275
gnl|TIGR|Helex EE642177
gnl|TIGR|Helpe TA1698 4234
gnl|TIGR|Helpe DY949734
gnl|TIGR|Lacpe TA1955 43195
gnl|TIGR|Lacsa TA1480 4236
gnl|TIGR|Lacse TA3915 75943
gnl|TIGR|Lacvi TA1665 75947
gnl|TIGR|Tarko TA1052 333970
gnl|TIGR|Tarof TA2006 50225
gnl|TIGR|Nicta DV158833
gnl|TIGR|Solch DN980235
gnl|TIGR|Soltu TA27893 4113
gnl|TIGR|Solly TA21021 4081
gnl|TIGR|Vitsh TA2311 246827
gnl|TIGR|Vitvi CF512012
Aspargus officinalis
Eschscholzia californica
gnl|TIGR|Horvu TA34888 4513
gnl|TIGR|Triae CJ672123
gnl|TIGR|Triae TA69844 4565
gnl|Orysa5|Os03g08450.1
gnl|TIGR|Sorbi TA25628 4558
gnl|TIGR|Sacof TA31473 4547
gnl|TIGR|Sacof TA31474 4547
gnl|TIGR|Zeama TA101464 4577
gnl|TIGR|Zeama TA95792 4577
Nuphar advena
Persea americana
Saruma henryi
gnl|TIGR|Zanae TA1367 69721
gnl|TIGR|Pinpi TA6380 71647
gnl|TIGR|Pinta TA9323 3352
Welwitschia mirabilis
gnl|TIGR|Picgl TA15404 3330
At4g33250 ML BS

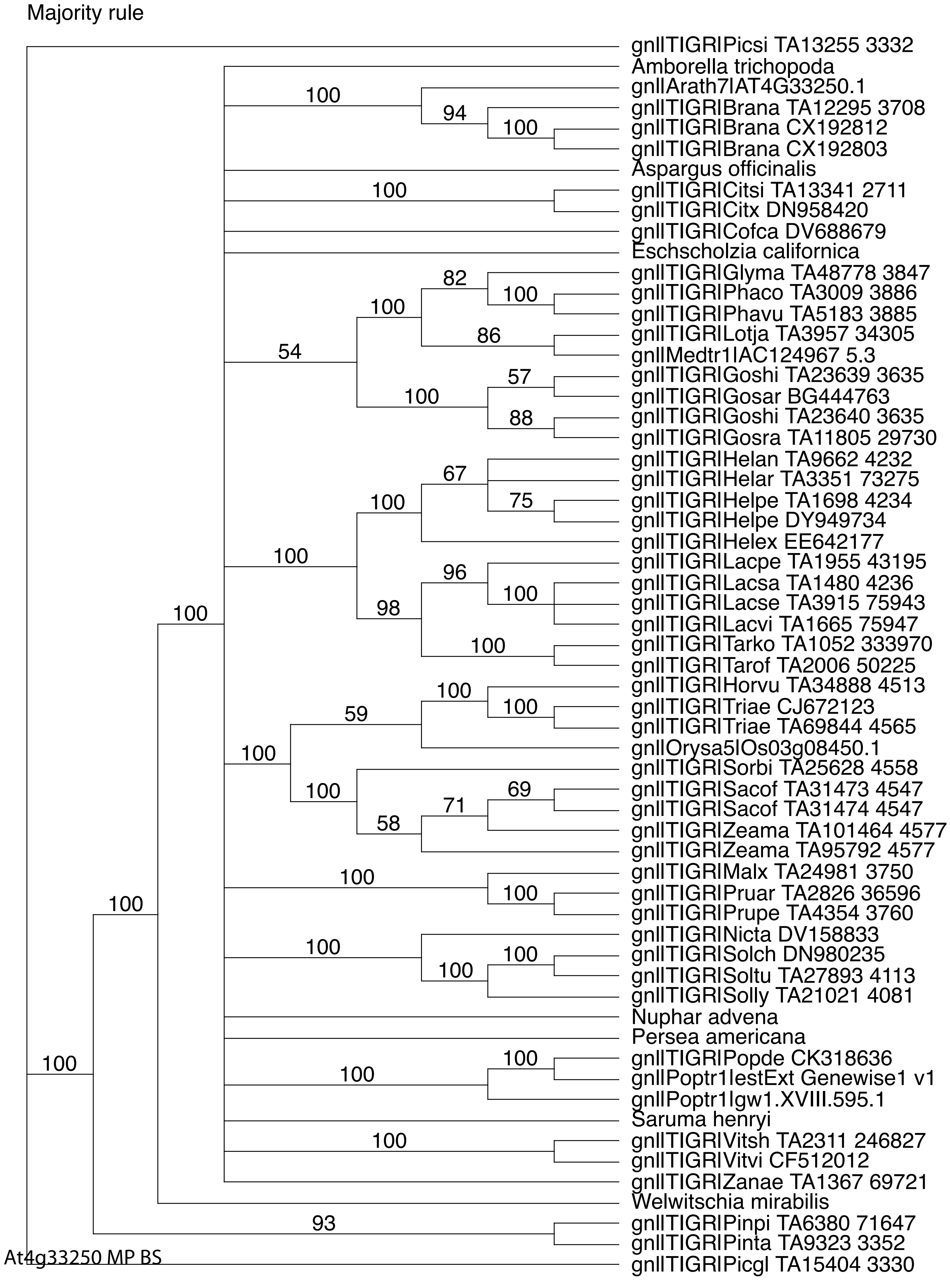
Majority rule
gnlITIGRIPicsi TA13255 3332
Amborella trichopoda
gnlArath7IAT4G33250.1
gnlITIGRIBrana TA12295 3708
gnlITIGRIBrana CX192812
gnlITIGRIBrana CX192803
Aspargus officinalis
gnlITIGRICitsi TA13341 2711
gnlITIGRICitx DN958420
gnlITIGRICofca DV688679
Eschscholzia californica
gnlITIGRIGlyma TA48778 3847
gnlITIGRIPhaco TA3009 3886
gnlITIGRIPhavu TA5183 3885
gnlITIGRILotja TA3957 34305
gnlMedtr1IAC124967 5.3
gnlITIGRIGoshi TA23639 3635
gnlITIGRIGosar BG444763
gnlITIGRIGoshi TA23640 3635
gnlITIGRIGosra TA11805 29730
gnlITIGRIHelan TA9662 4232
gnlITIGRIHelar TA3351 73275
gnlITIGRIHelpe TA1698 4234
gnlITIGRIHelpe DY949734
gnlITIGRIHelex EE642177
gnlITIGRILacpe TA1955 43195
gnlITIGRILacsa TA1480 4236
gnlITIGRILacse TA3915 75943
gnlITIGRILacvi TA1665 75947
gnlITIGRITarko TA1052 333970
gnlITIGRITarof TA2006 50225
gnlITIGRIHorvu TA34888 4513
gnlITIGRITriae CJ672123
gnlITIGRITriae TA69844 4565
gnlOrysa5IOs03g08450.1
gnlITIGRISorbi TA25628 4558
gnlITIGRISacof TA31473 4547
gnlITIGRISacof TA31474 4547
gnlITIGRIZeama TA101464 4577
gnlITIGRIZeama TA95792 4577
gnlITIGRIMalx TA24981 3750
gnlITIGRIPruar TA2826 36596
gnlITIGRIPrupe TA4354 3760
gnlITIGRINicta DV158833
gnlITIGRISolch DN980235
gnlITIGRISoltu TA27893 4113
gnlITIGRISolly TA21021 4081
Nuphar advena
Persea americana
gnlITIGRIPopde CK318636
gnlPoptr1IestExt Genewise1 v1
gnlPoptr1Igw1.XVIII.595.1
Saruma henryi
gnlITIGRIVitsh TA2311 246827
gnlITIGRIVitvi CF512012
gnlITIGRIZanae TA1367 69721
Welwitschia mirabilis
gnlITIGRIPinpi TA6380 71647
gnlITIGRIPinta TA9323 3352
gnlITIGRIPicgl TA15404 3330
At4g33250 MP BS

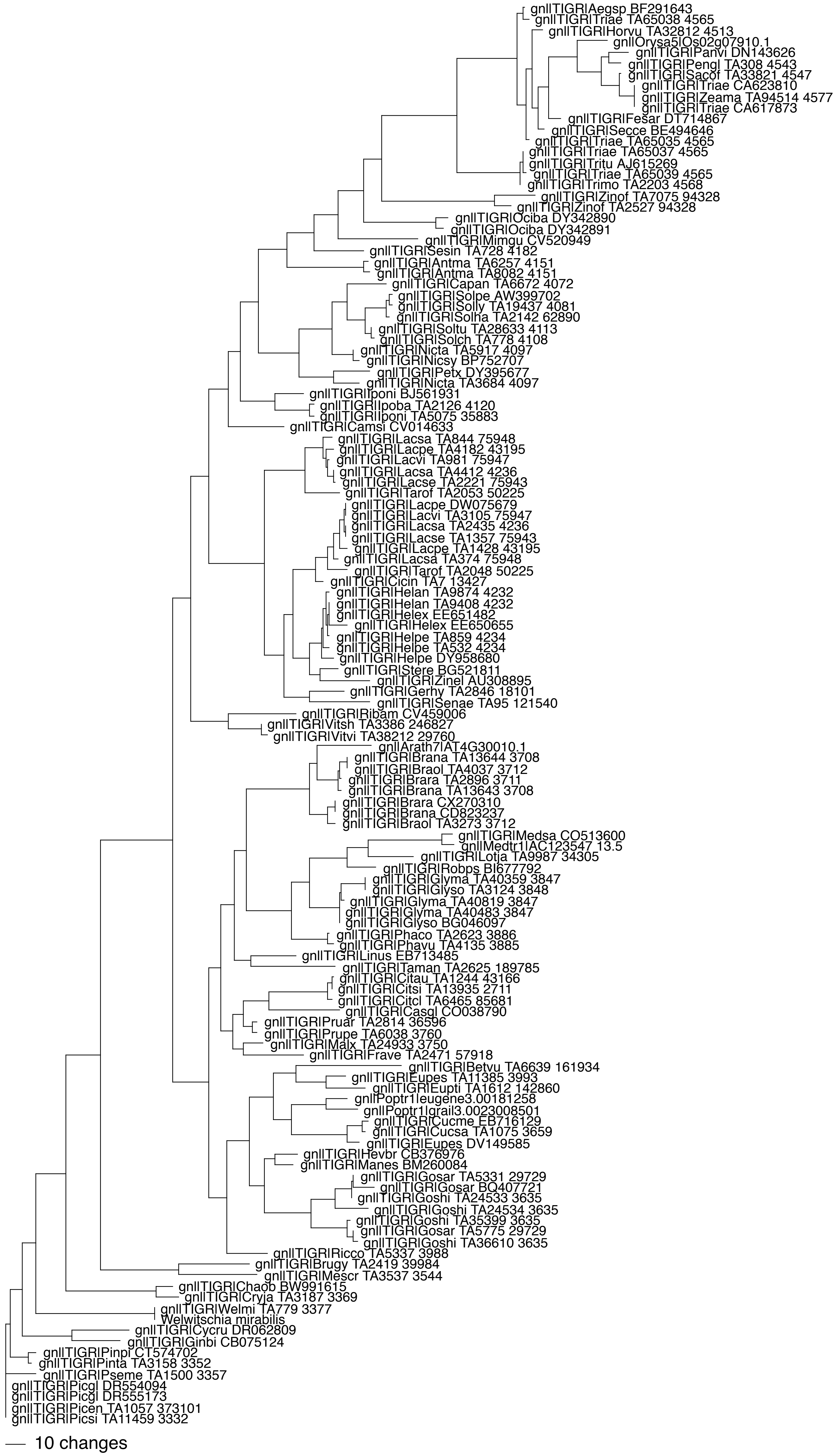

At4g30010 ML

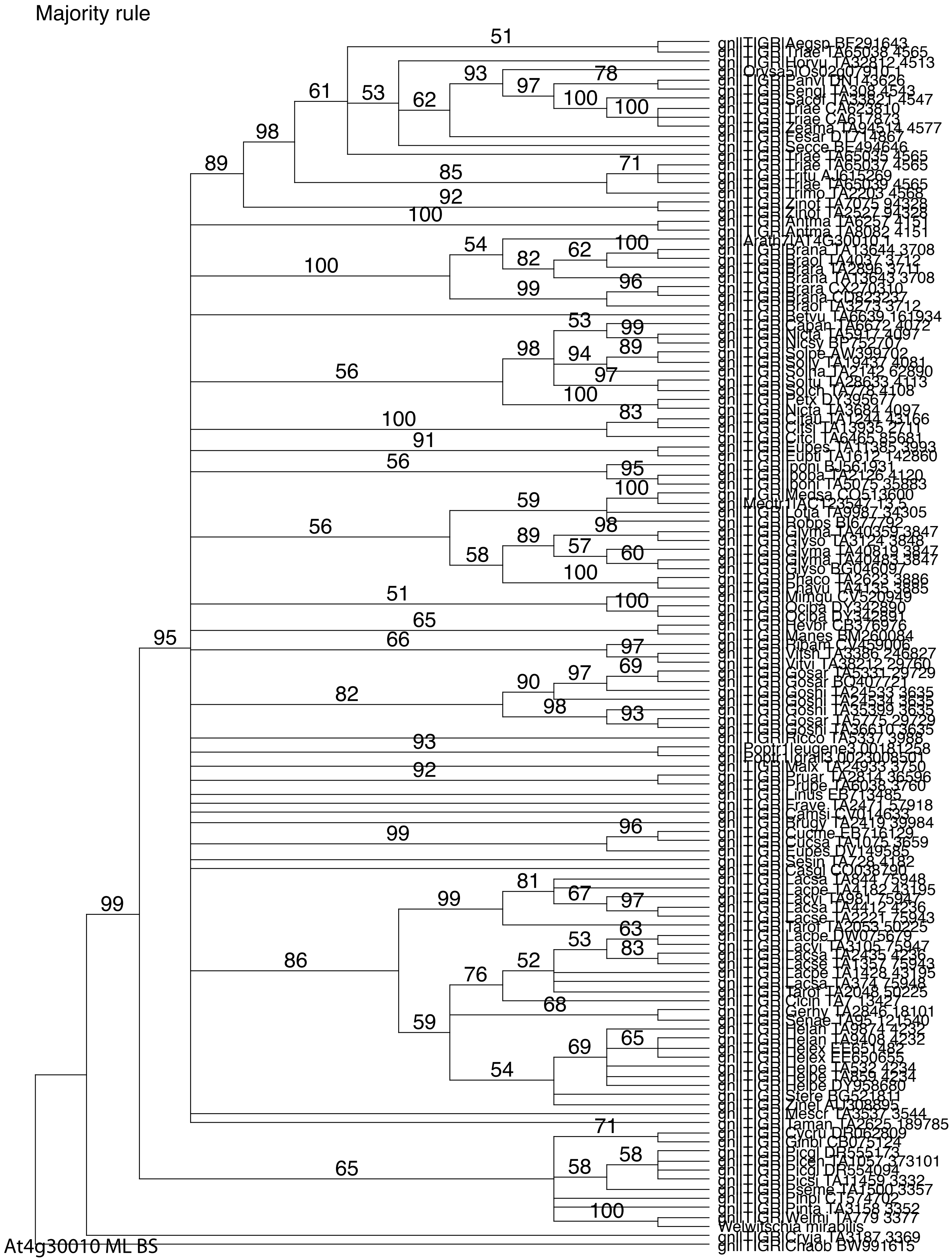
Majority rule
Welwitschia mirabilis
At4g30010 ML BS

Majority rule

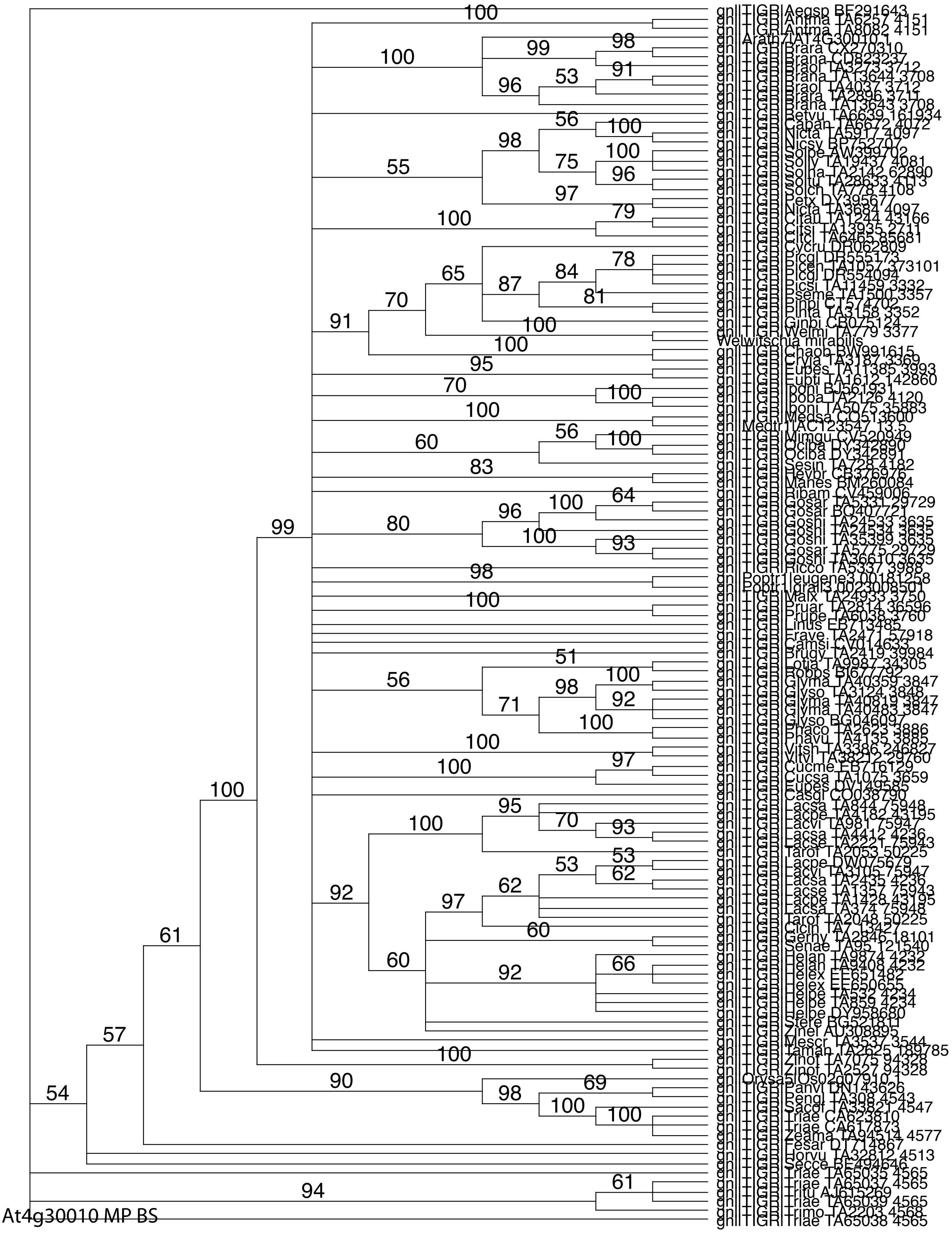

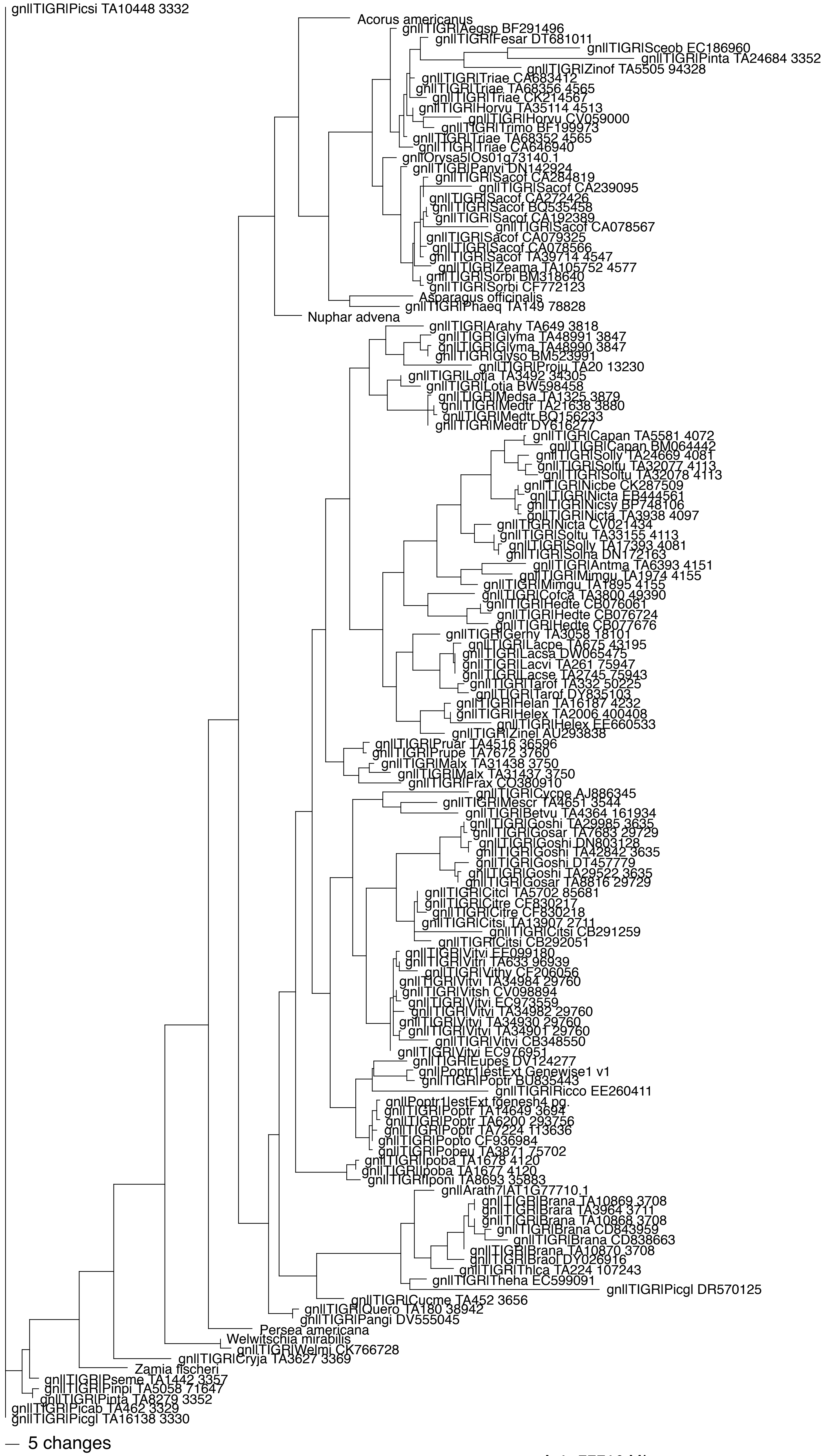

At1g77710 ML

Majority rule

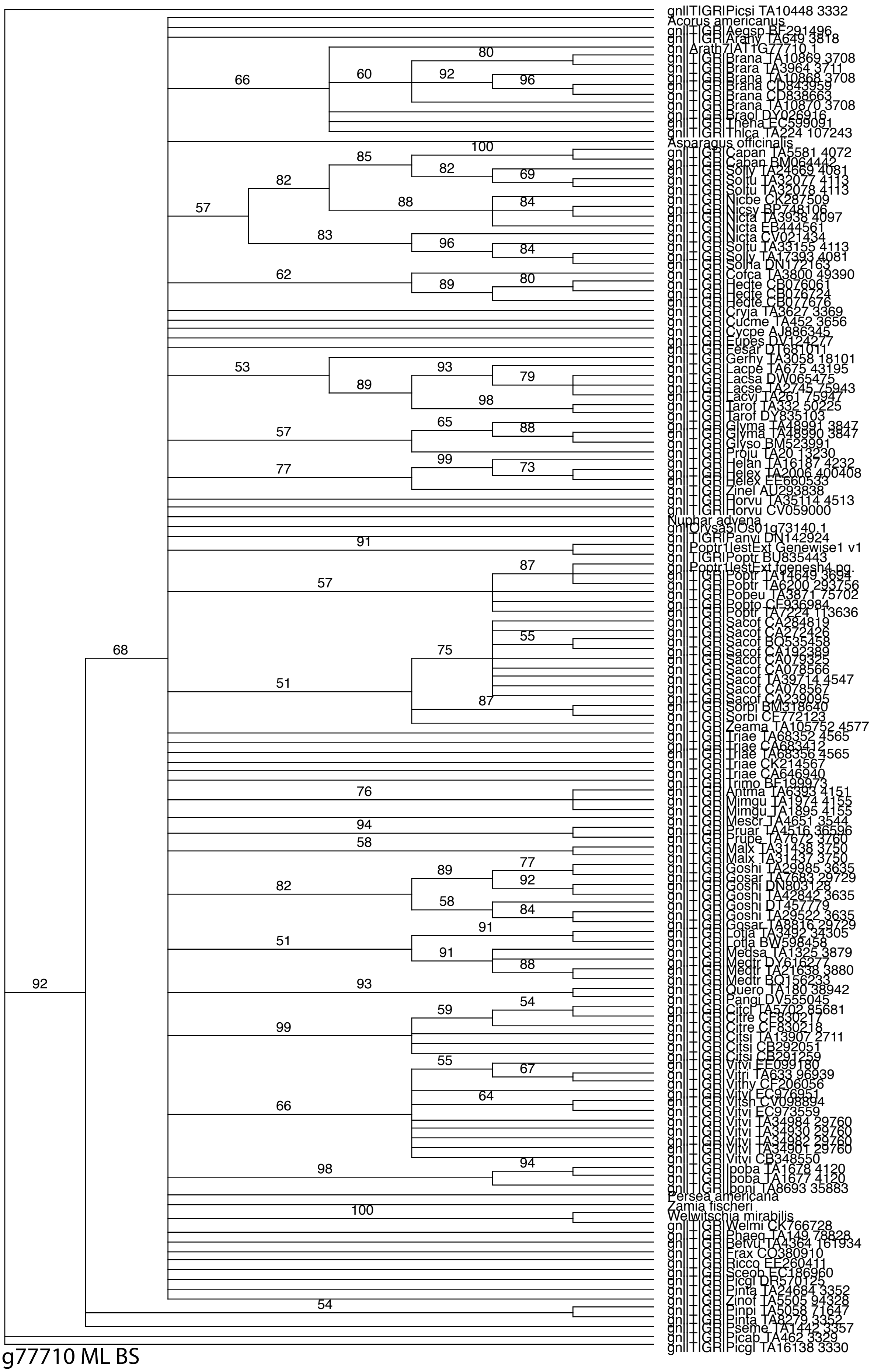

At1g77710 ML BS

Majority rule

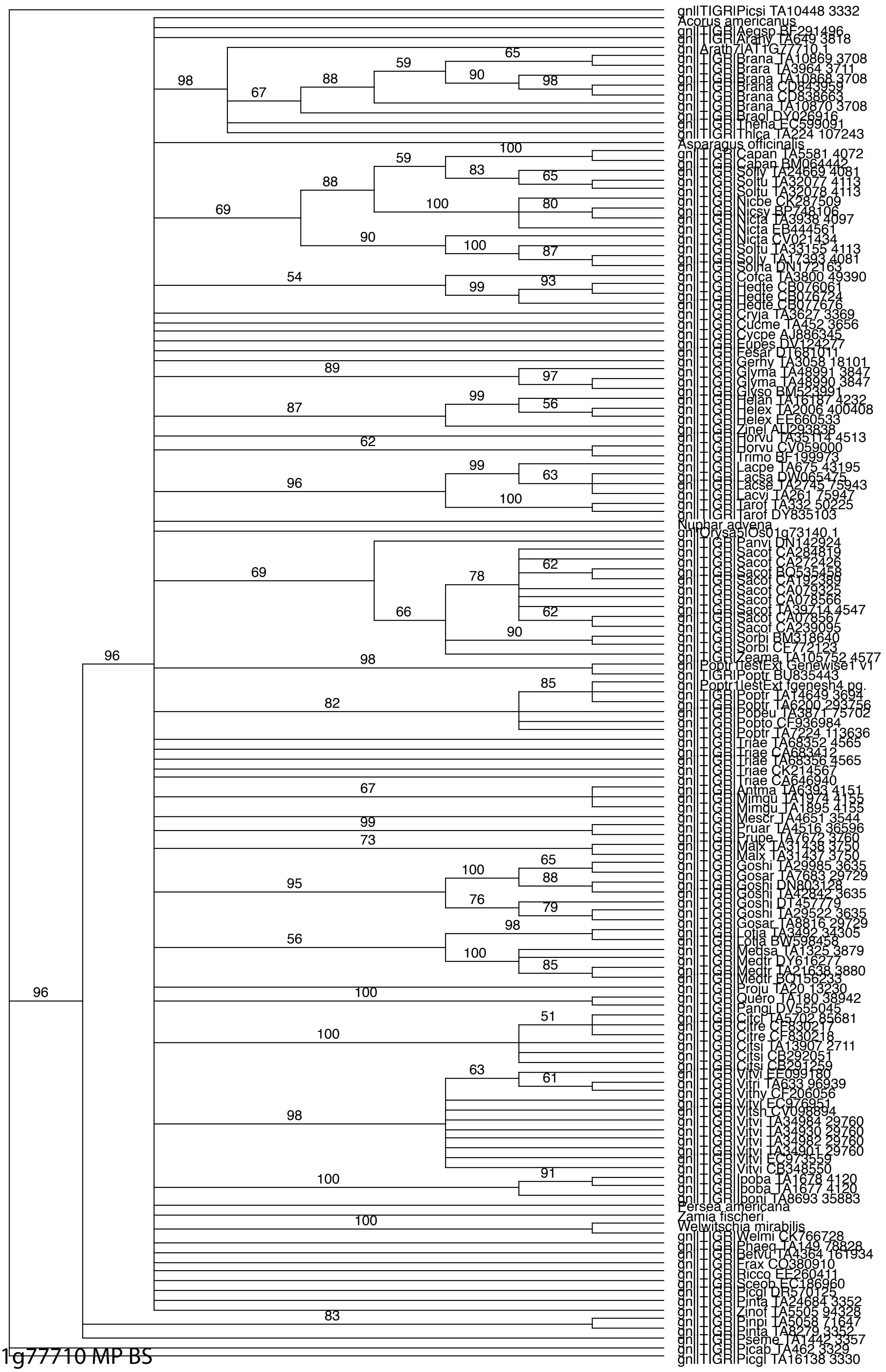

At1g77710 MP BS

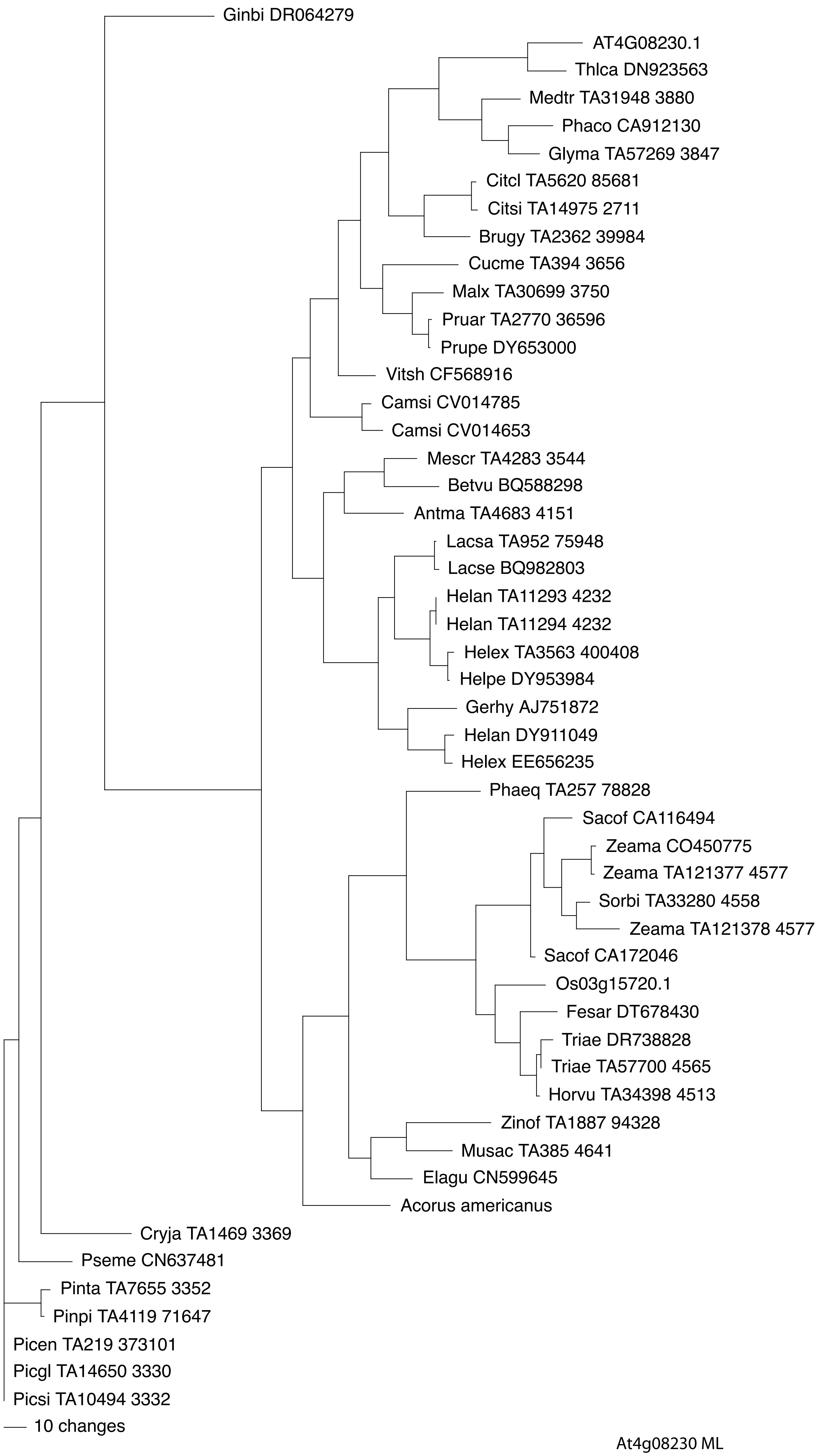

At4g08230 ML

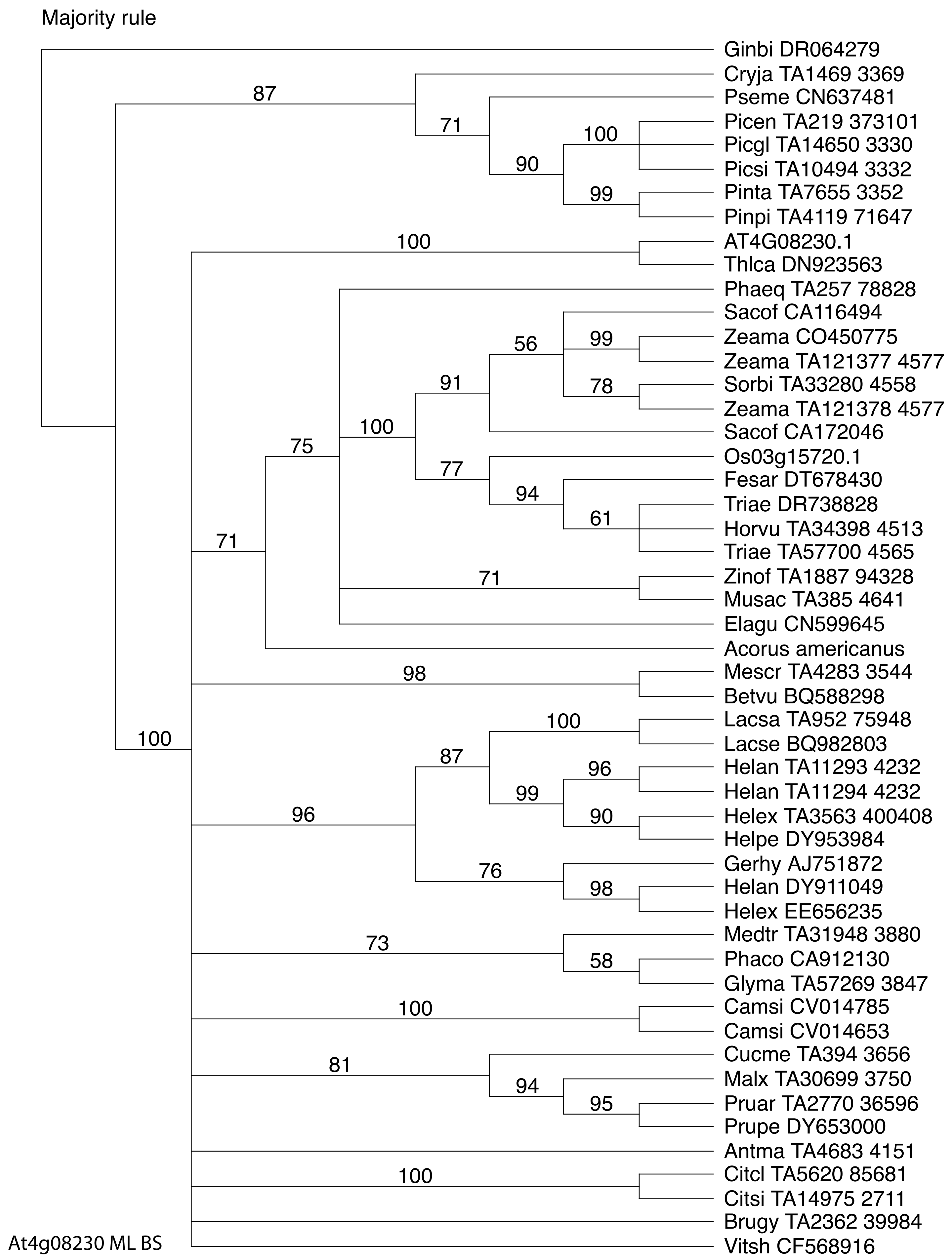
Majority rule
Ginbi DR064279
Cryja TA1469 3369
Pseme CN637481
Picen TA219 373101
Picgl TA14650 3330
Picsi TA10494 3332
Pinta TA7655 3352
Pinpi TA4119 71647
AT4G08230.1
Thlca DN923563
Phaeq TA257 78828
Sacof CA116494
Zeama CO450775
Zeama TA121377 4577
Sorbi TA33280 4558
Zeama TA121378 4577
Sacof CA172046
Os03g15720.1
Fesar DT678430
Triae DR738828
Horvu TA34398 4513
Triae TA57700 4565
Zinof TA1887 94328
Musac TA385 4641
Elagu CN599645
Acorus americanus
Mescr TA4283 3544
Betvu BQ588298
Lacsa TA952 75948
Lacse BQ982803
Helan TA11293 4232
Helan TA11294 4232
Helex TA3563 400408
Helpe DY953984
Gerhy AJ751872
Helan DY911049
Helex EE656235
Medtr TA31948 3880
Phaco CA912130
Glyma TA57269 3847
Camsi CV014785
Camsi CV014653
Cucme TA394 3656
Malx TA30699 3750
Pruar TA2770 36596
Prupe DY653000
Antma TA4683 4151
Citcl TA5620 85681
Citsi TA14975 2711
Brugy TA2362 39984
Vitsh CF568916
At4g08230 ML BS

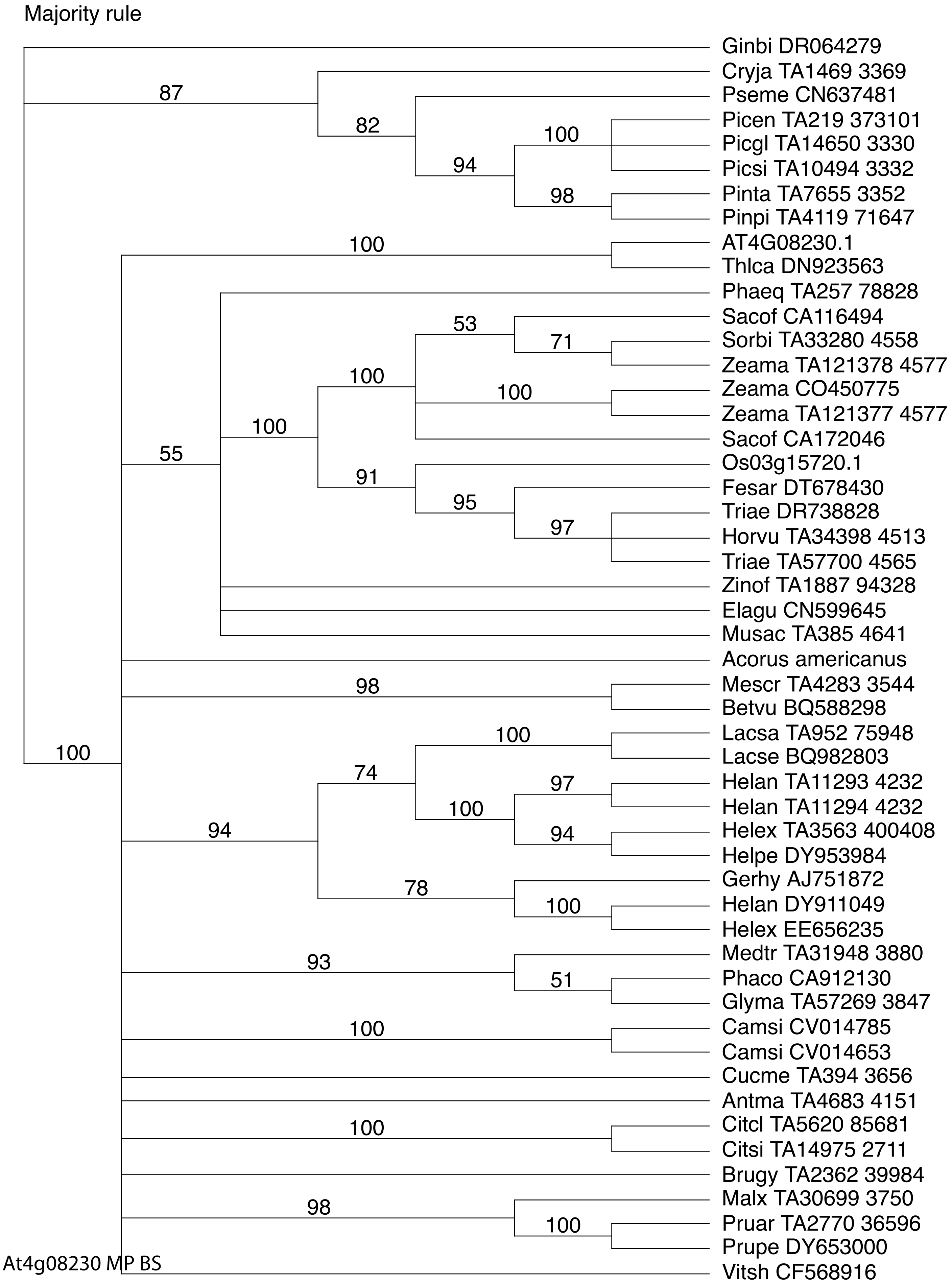
Majority rule
Ginbi DR064279
Cryja TA1469 3369
Pseme CN637481
Picen TA219 373101
Picgl TA14650 3330
Picsi TA10494 3332
Pinta TA7655 3352
Pinpi TA4119 71647
AT4G08230.1
Thlca DN923563
Phaeq TA257 78828
Sacof CA116494
Sorbi TA33280 4558
Zeama TA121378 4577
Zeama CO450775
Zeama TA121377 4577
Sacof CA172046
Os03g15720.1
Fesar DT678430
Triae DR738828
Horvu TA34398 4513
Triae TA57700 4565
Zinof TA1887 94328
Elagu CN599645
Musac TA385 4641
Acorus americanus
Mescr TA4283 3544
Betvu BQ588298
Lacsa TA952 75948
Lacse BQ982803
Helan TA11293 4232
Helan TA11294 4232
Helex TA3563 400408
Helpe DY953984
Gerhy AJ751872
Helan DY911049
Helex EE656235
Medtr TA31948 3880
Phaco CA912130
Glyma TA57269 3847
Camsi CV014785
Camsi CV014653
Cucme TA394 3656
Antma TA4683 4151
Citcl TA5620 85681
Citsi TA14975 2711
Brugy TA2362 39984
Malx TA30699 3750
Pruar TA2770 36596
Prupe DY653000
Vitsh CF568916
87
82
94
100
98
100
55
100
100
53
71
100
91
95
97
98
100
94
74
100
100
97
94
78
100
93
51
100
100
98
100
At4g08230 MP BS

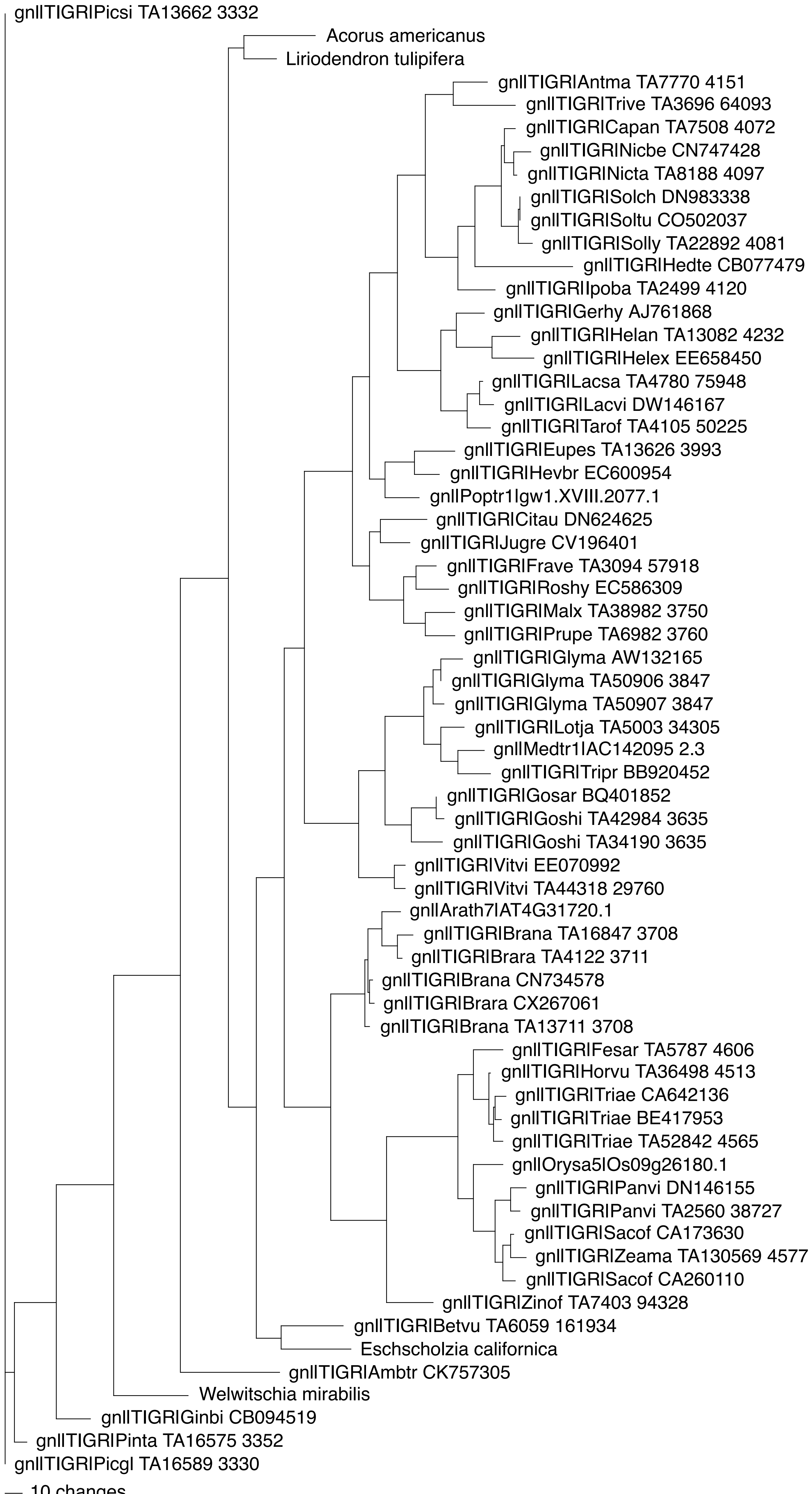

At4g31720 ML

Majority rule

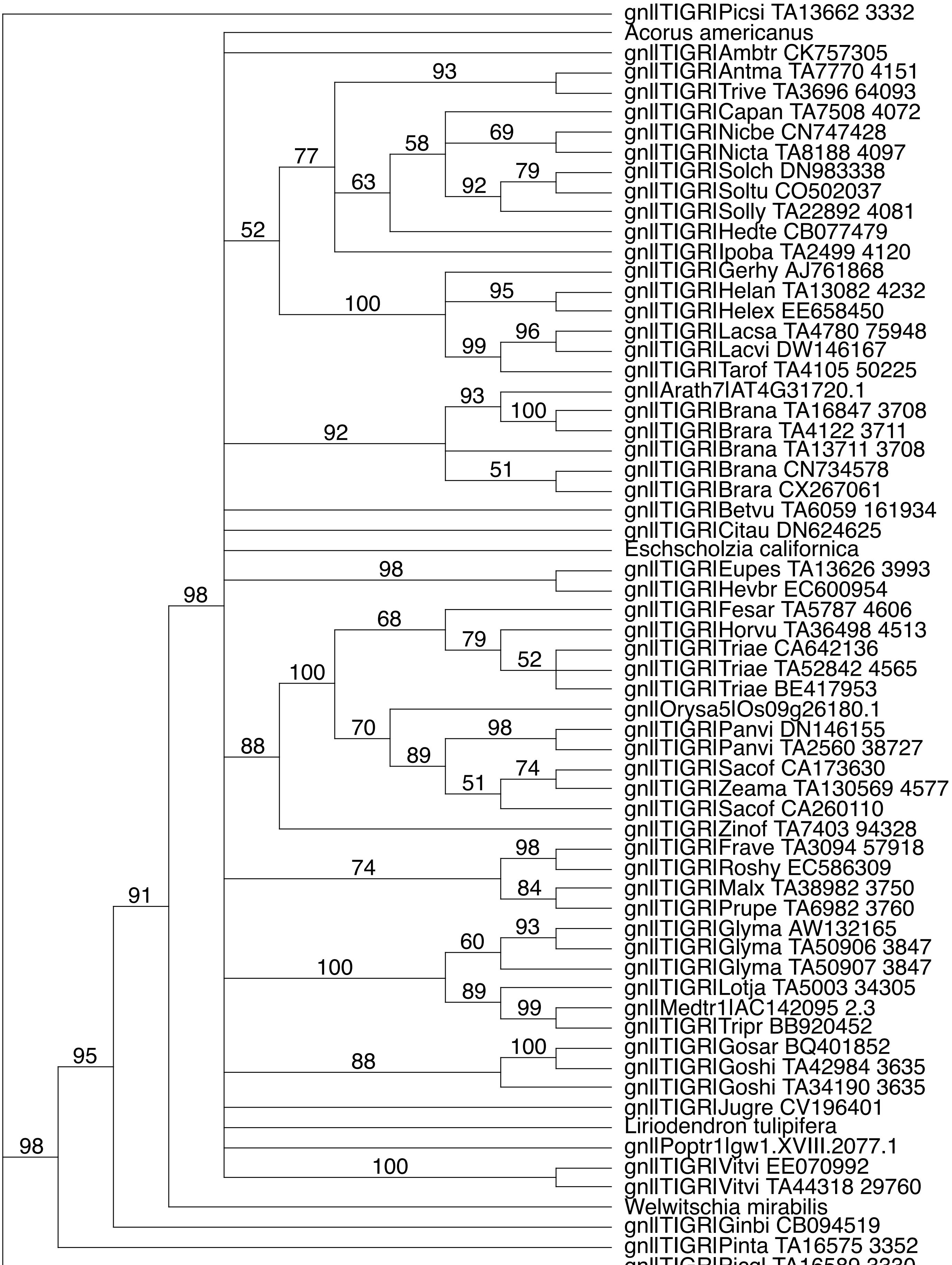

At4g31720 ML BS

Majority rule

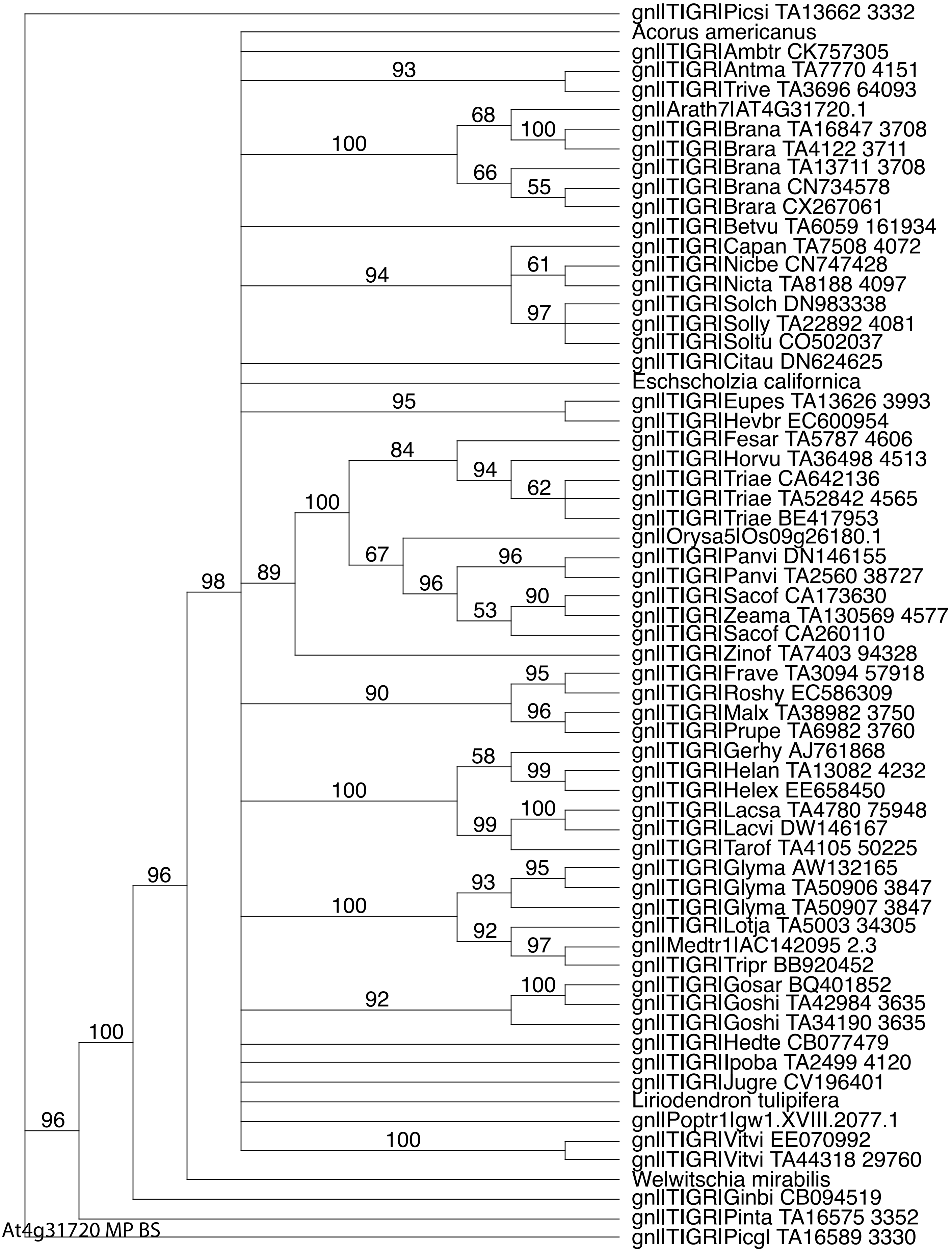

At4g31720 MP BS

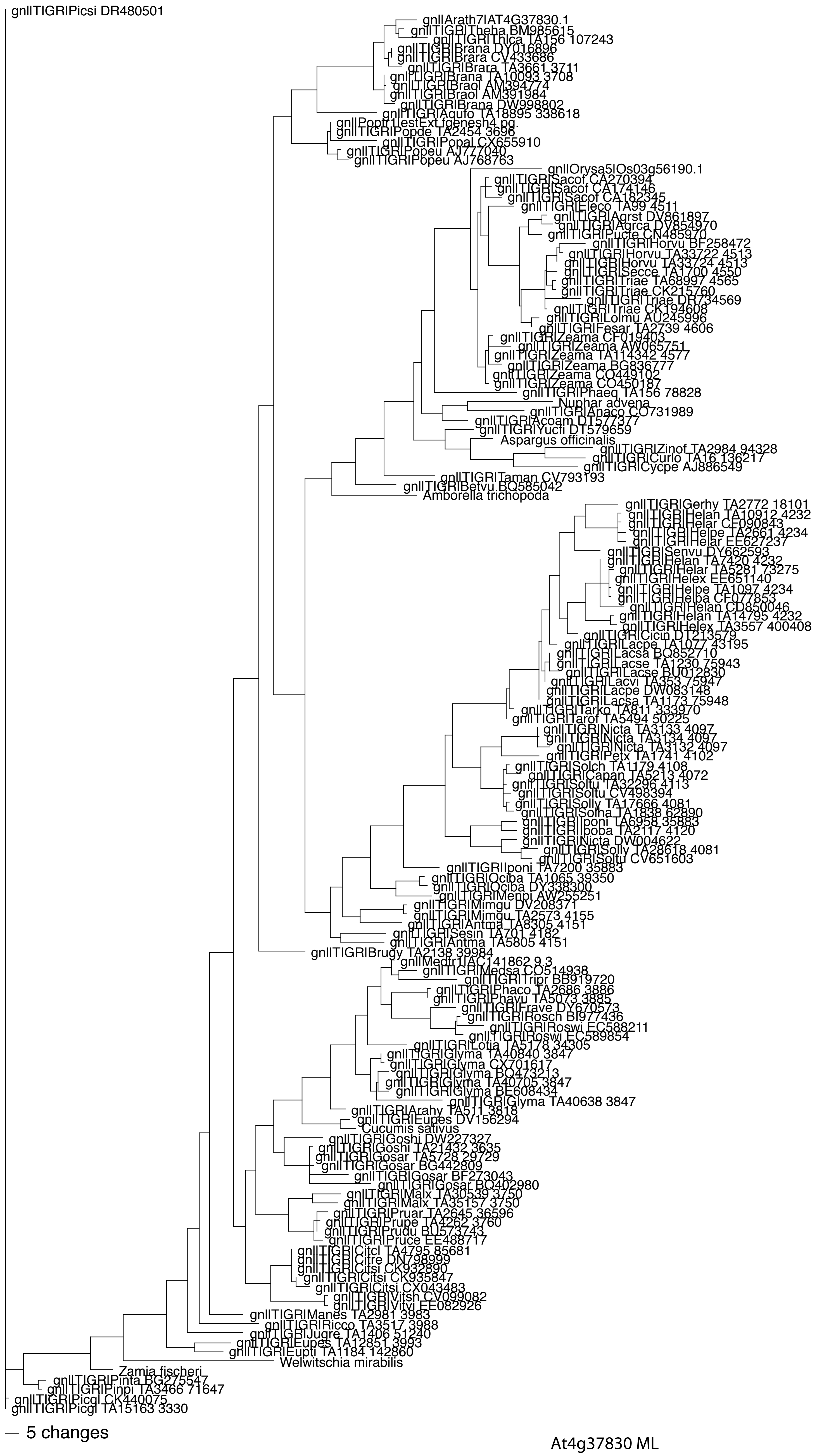

At4g37830 ML

Majority rule

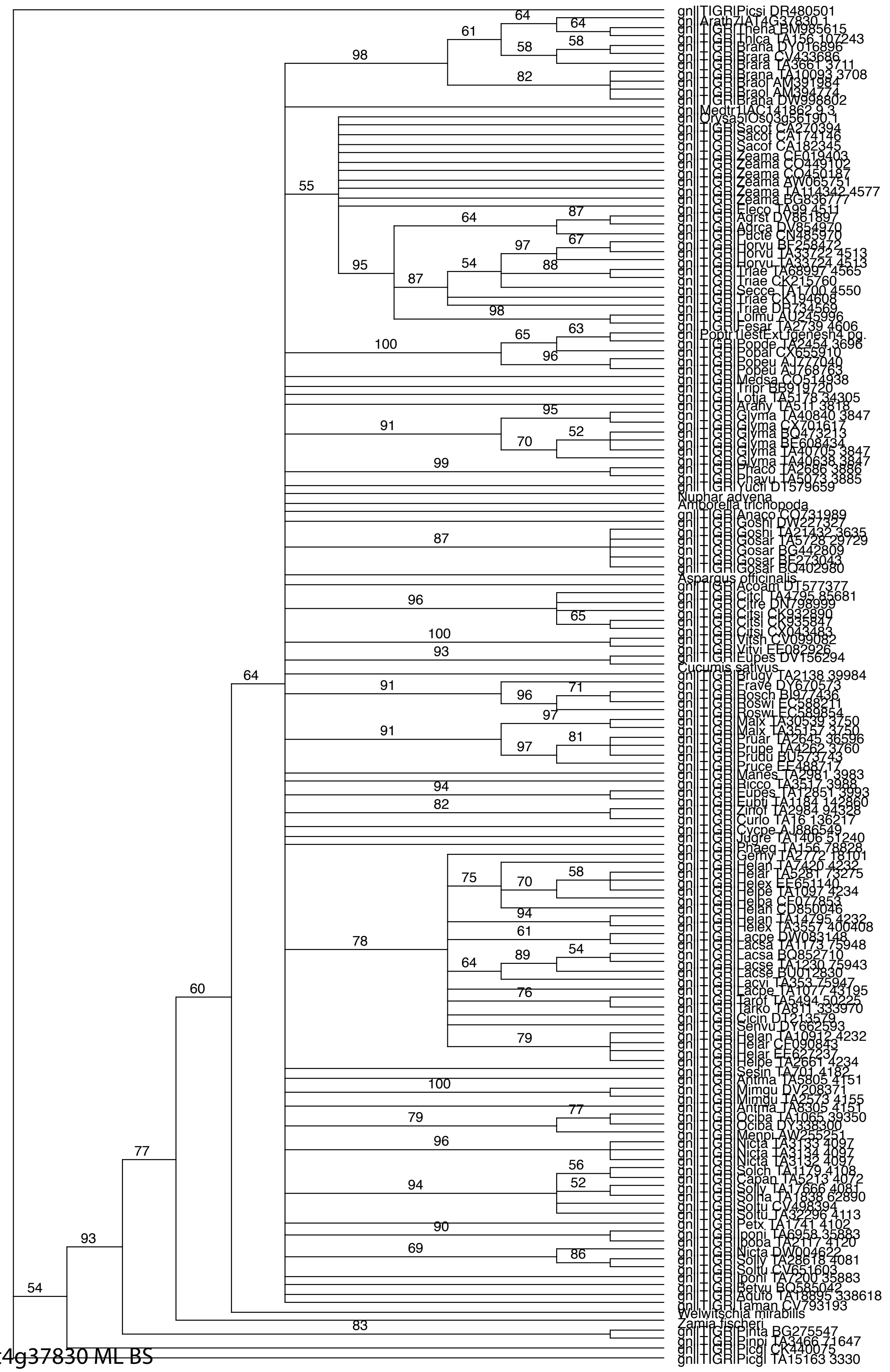

At4g37830 ML BS

Majority rule

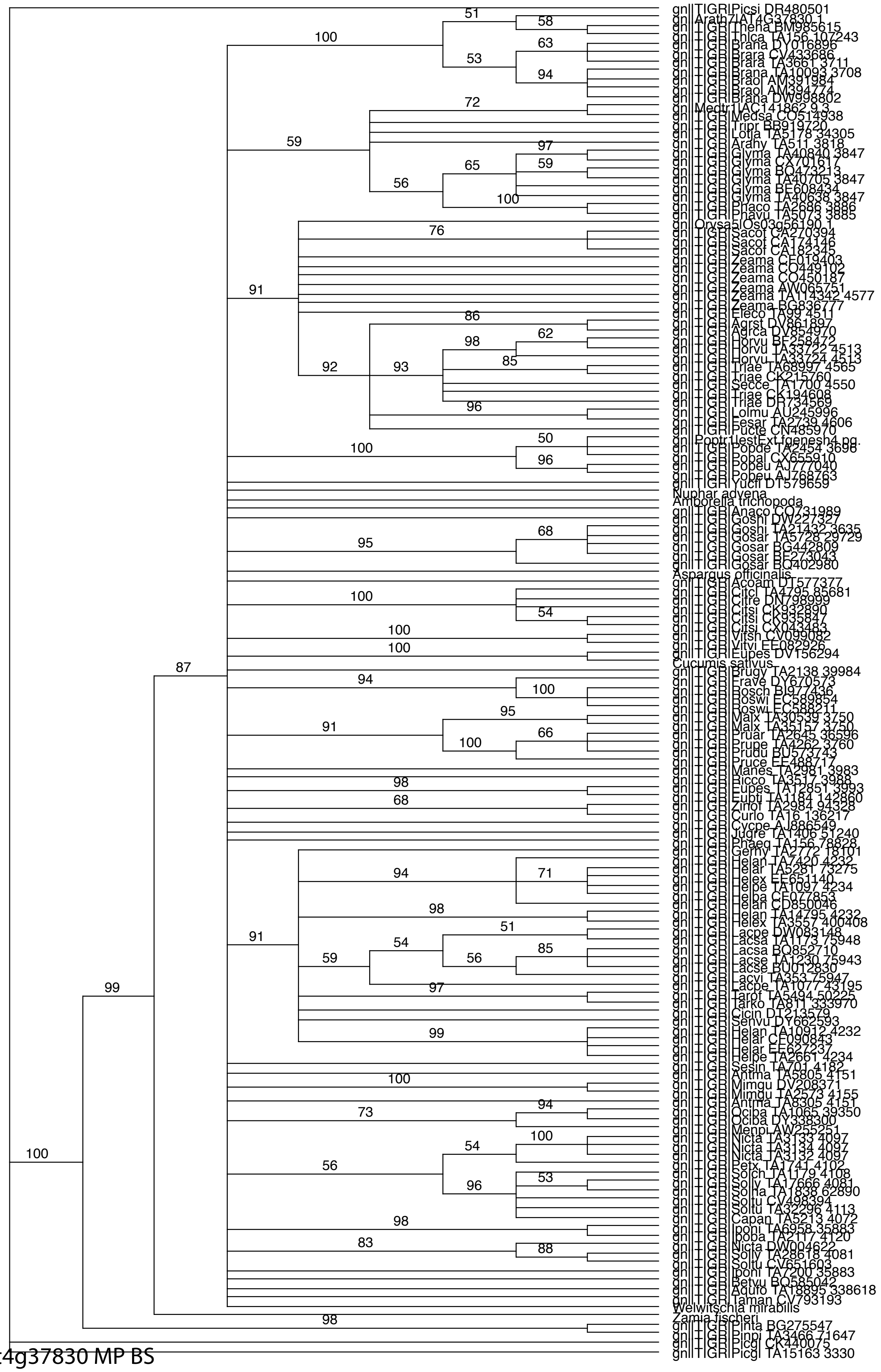

At4g37830 MP BS

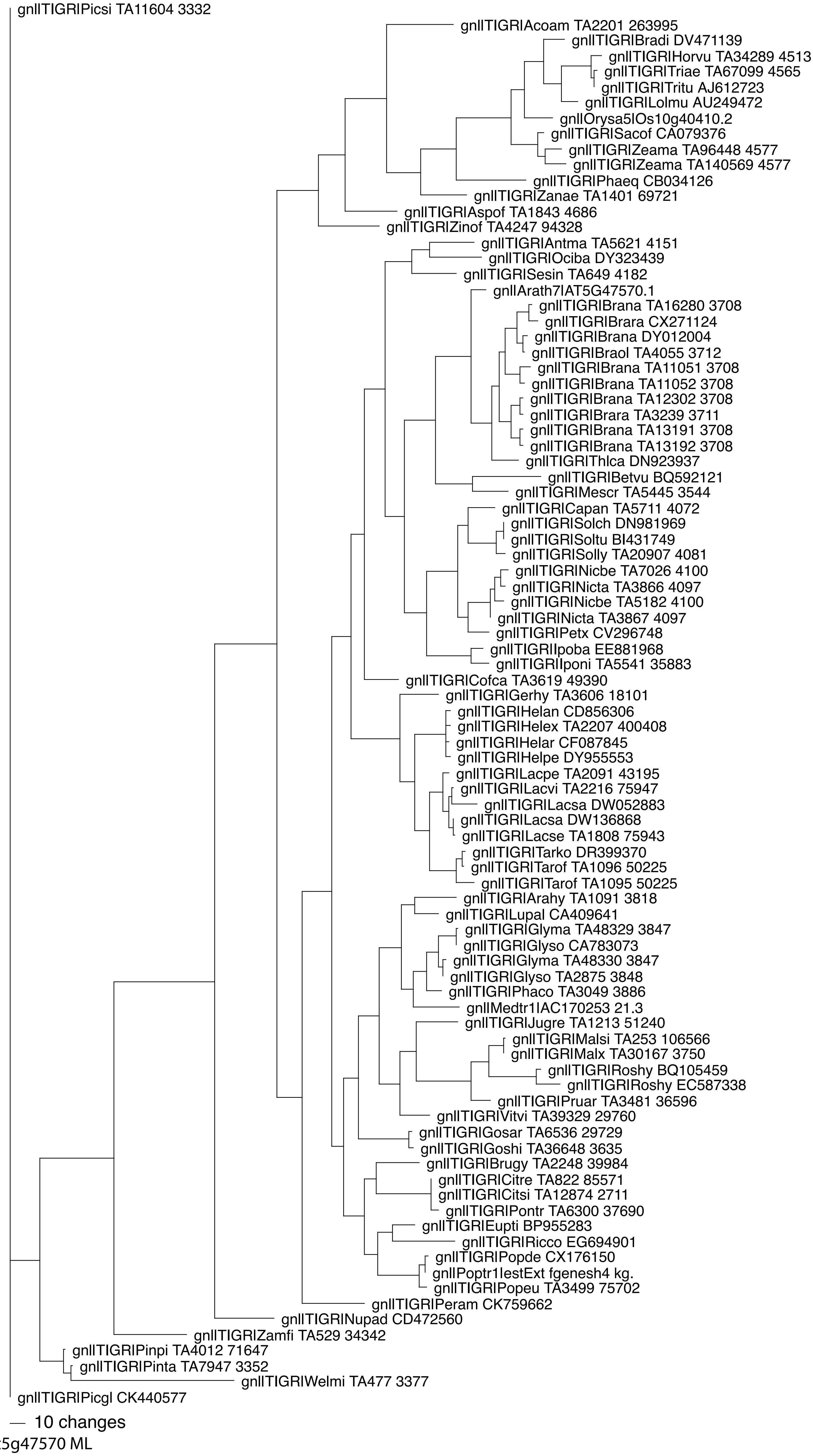

At5g47570 ML

Majority rule

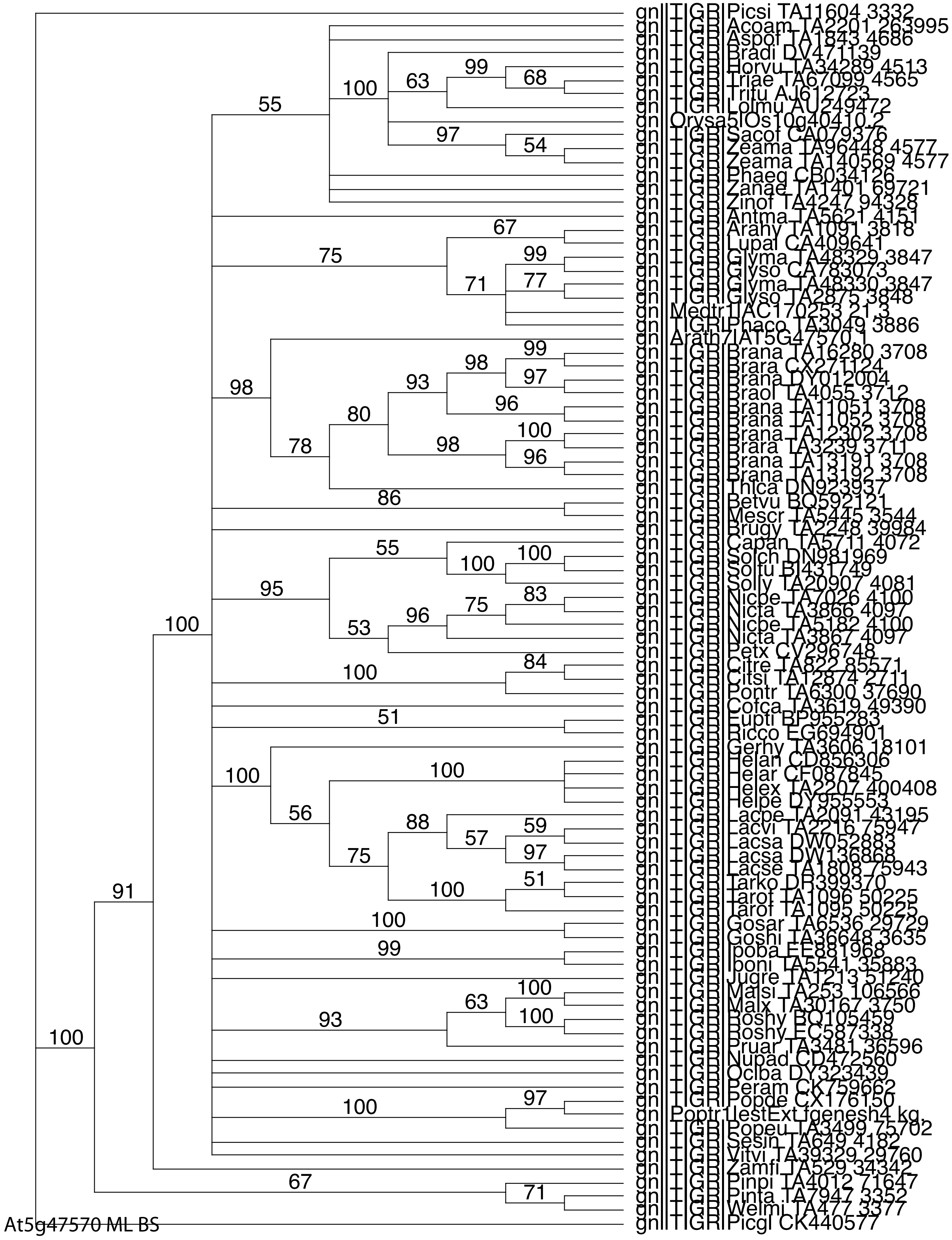

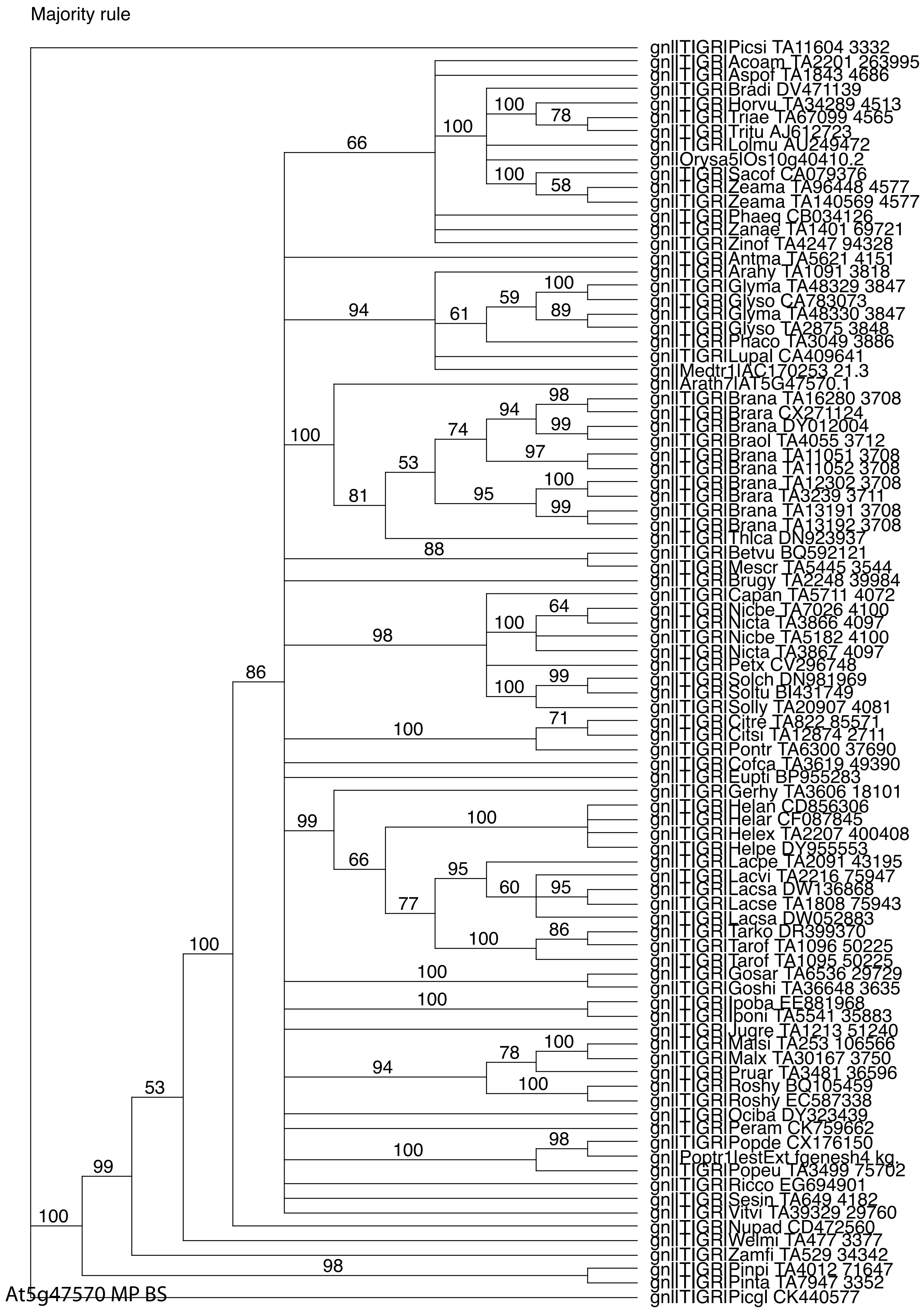
Majority rule
gnl|TIGR|Picsi TA11604 3332
gnl|TIGR|Acoam TA2201 263995
gnl|TIGR|Aspof TA1843 4686
gnl|TIGR|Bradi DV471139
gnl|TIGR|Horvu TA34289 4513
gnl|TIGR|Triae TA67099 4565
gnl|TIGR|Tritu AJ612723
gnl|TIGR|Lolmu AU249472
gnl|Orysa5|Os10g40410.2
gnl|TIGR|Sacof CA079376
gnl|TIGR|Zeama TA96448 4577
gnl|TIGR|Zeama TA140569 4577
gnl|TIGR|Phaeq CB034126
gnl|TIGR|Zanae TA1401 69721
gnl|TIGR|Zinof TA4247 94328
gnl|TIGR|Antma TA5621 4151
gnl|TIGR|Arahy TA1091 3818
gnl|TIGR|Glyma TA48329 3847
gnl|TIGR|Glyso CA783073
gnl|TIGR|Glyma TA48330 3847
gnl|TIGR|Glyso TA2875 3848
gnl|TIGR|Phaco TA3049 3886
gnl|TIGR|Lupal CA409641
gnl|Medtr1|AC170253 21.3
gnl|Arath7|AT5G47570.1
gnl|TIGR|Brana TA16280 3708
gnl|TIGR|Brara CX271124
gnl|TIGR|Brana DY012004
gnl|TIGR|Braol TA4055 3712
gnl|TIGR|Brana TA11051 3708
gnl|TIGR|Brana TA11052 3708
gnl|TIGR|Brana TA12302 3708
gnl|TIGR|Brara TA3239 3711
gnl|TIGR|Brana TA13191 3708
gnl|TIGR|Brana TA13192 3708
gnl|TIGR|Thlca DN923937
gnl|TIGR|Betvu BQ592121
gnl|TIGR|Mescr TA5445 3544
gnl|TIGR|Brugy TA2248 39984
gnl|TIGR|Capan TA5711 4072
gnl|TIGR|Nicbe TA7026 4100
gnl|TIGR|Nicta TA3866 4097
gnl|TIGR|Nicbe TA5182 4100
gnl|TIGR|Nicta TA3867 4097
gnl|TIGR|Petx CV296748
gnl|TIGR|Solch DN981969
gnl|TIGR|Soltu BI431749
gnl|TIGR|Solly TA20907 4081
gnl|TIGR|Citre TA822 85571
gnl|TIGR|Citsi TA12874 2711
gnl|TIGR|Pontr TA6300 37690
gnl|TIGR|Cofca TA3619 49390
gnl|TIGR|Eupti BP955283
gnl|TIGR|Gerhy TA3606 18101
gnl|TIGR|Helan CD856306
gnl|TIGR|Helar CF087845
gnl|TIGR|Helex TA2207 400408
gnl|TIGR|Helpe DY955553
gnl|TIGR|Lacpe TA2091 43195
gnl|TIGR|Lacvi TA2216 75947
gnl|TIGR|Lacsa DW136868
gnl|TIGR|Lacse TA1808 75943
gnl|TIGR|Lacsa DW052883
gnl|TIGR|Tarko DR399370
gnl|TIGR|Tarof TA1096 50225
gnl|TIGR|Tarof TA1095 50225
gnl|TIGR|Gosar TA6536 29729
gnl|TIGR|Goshi TA36648 3635
gnl|TIGR|Ipoba EE881968
gnl|TIGR|Iponi TA5541 35883
gnl|TIGR|Jugre TA1213 51240
gnl|TIGR|Malsi TA253 106566
gnl|TIGR|Malx TA30167 3750
gnl|TIGR|Pruar TA3481 36596
gnl|TIGR|Roshy BQ105459
gnl|TIGR|Roshy EC587338
gnl|TIGR|Ociba DY323439
gnl|TIGR|Peram CK759662
gnl|TIGR|Popde CX176150
gnl|Poptr1|estExt fgenesh4 kg.
gnl|TIGR|Popeu TA3499 75702
gnl|TIGR|Ricco EG694901
gnl|TIGR|Sesin TA649 4182
gnl|TIGR|Vitvi TA39329 29760
gnl|TIGR|Nupad CD472560
gnl|TIGR|Welmi TA477 3377
gnl|TIGR|Zamfi TA529 34342
gnl|TIGR|Pinpi TA4012 71647
gnl|TIGR|Pinta TA7947 3352
gnl|TIGR|Picgl CK440577
At5g47570 MP BS

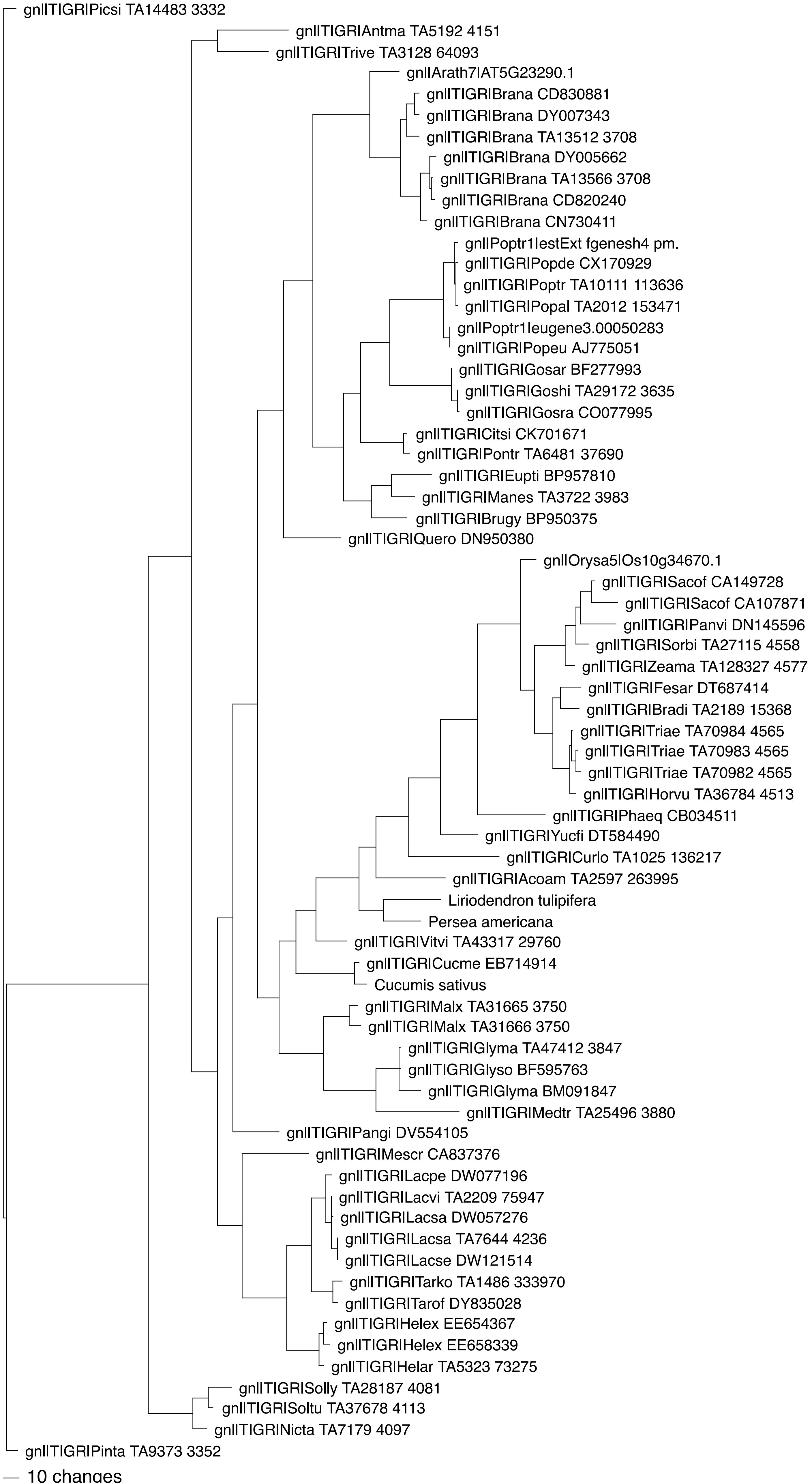

At5g23290 ML

Majority rule

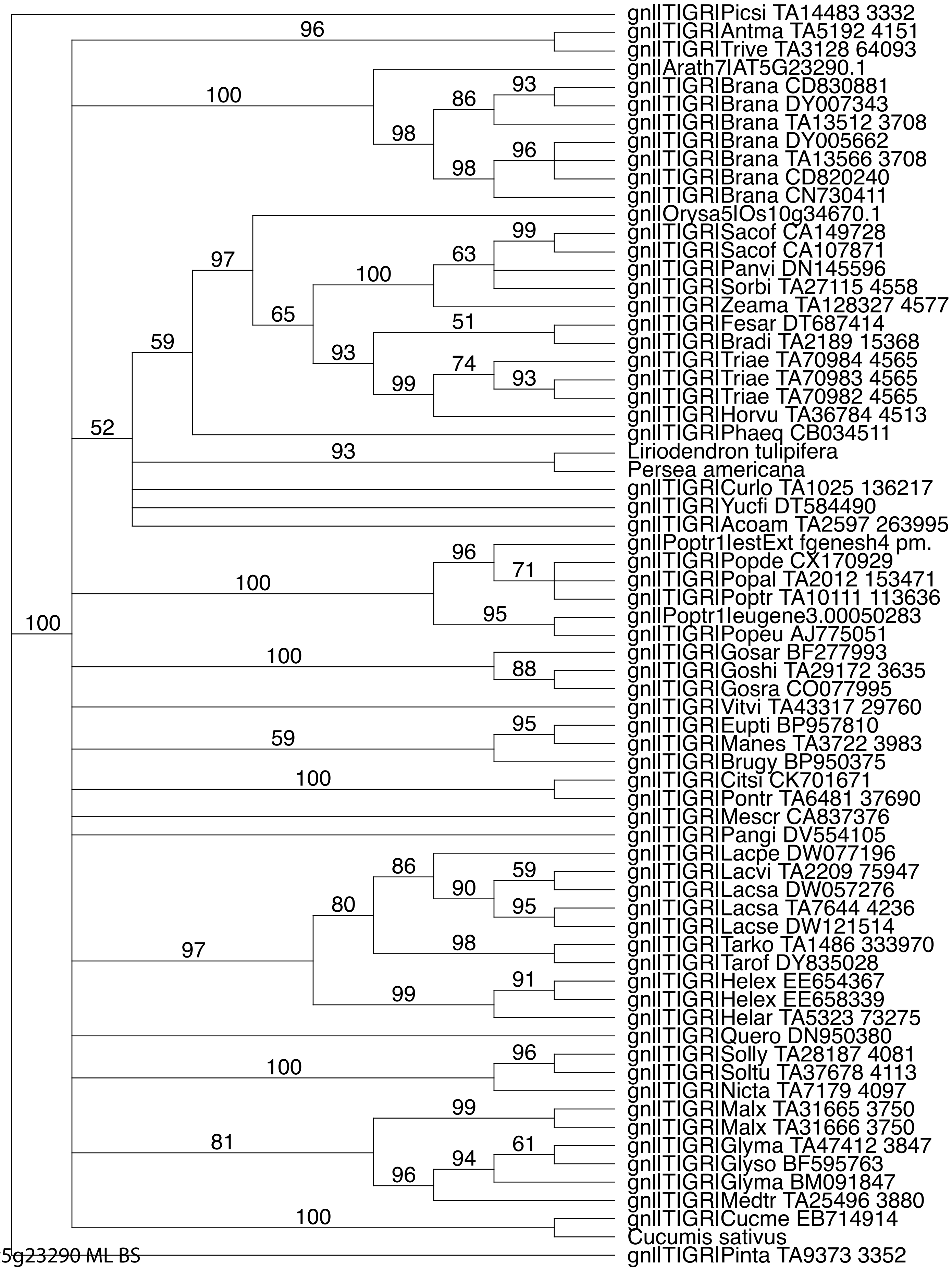

At5g23290 ML BS

Majority rule

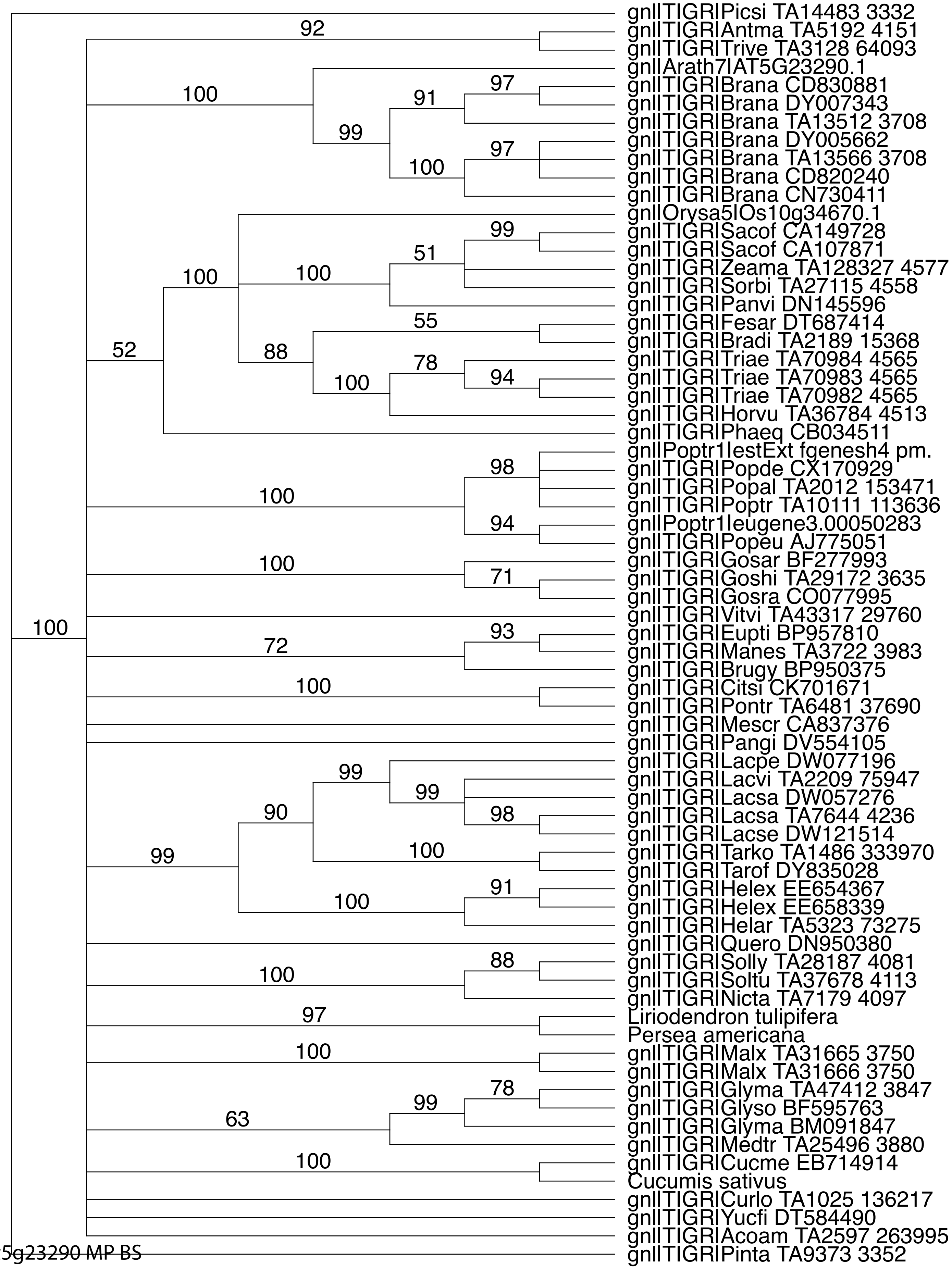

At5g23290 MP BS

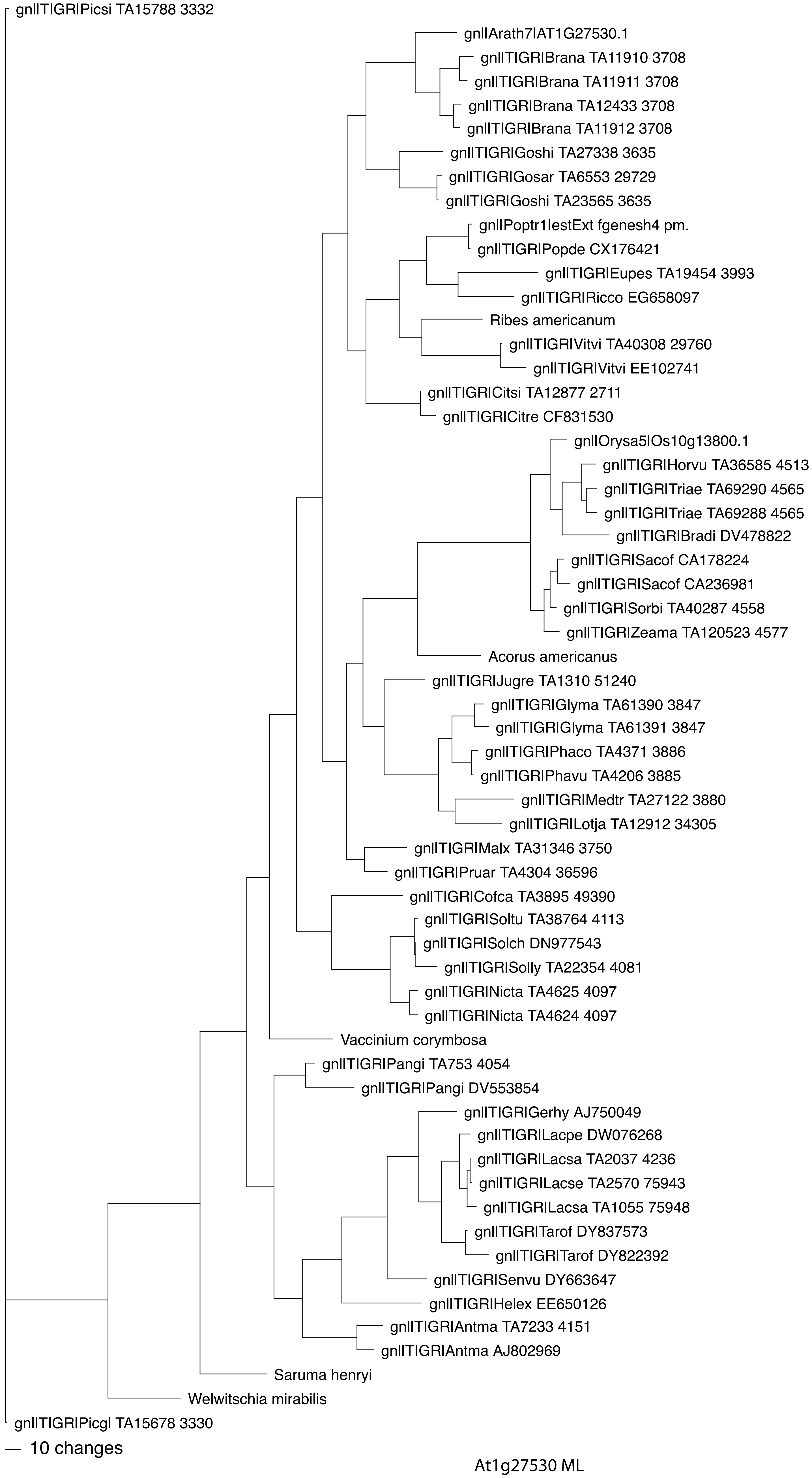

At1g27530 ML

Majority rule

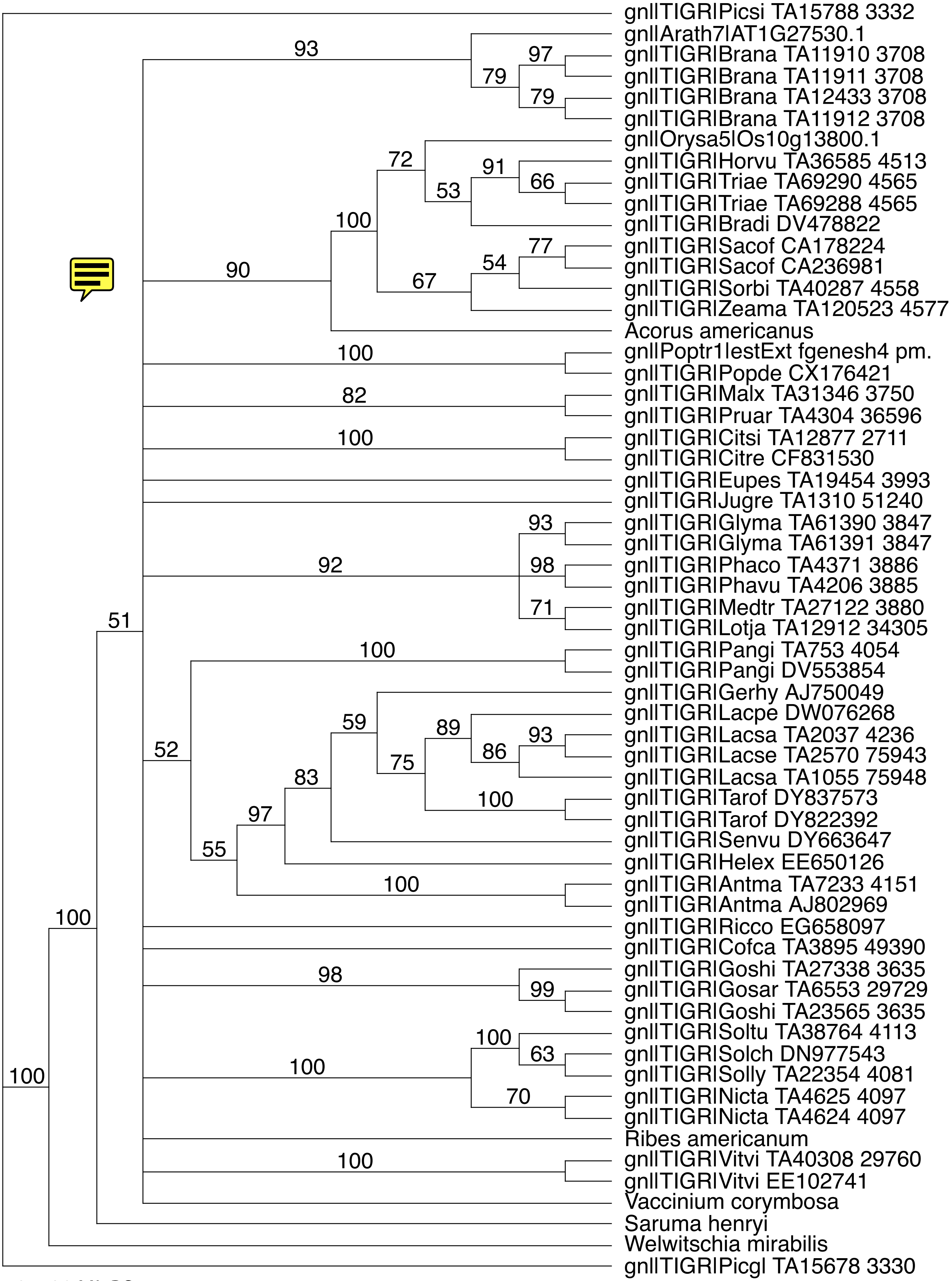

At1g27530 ML BS

Majority rule

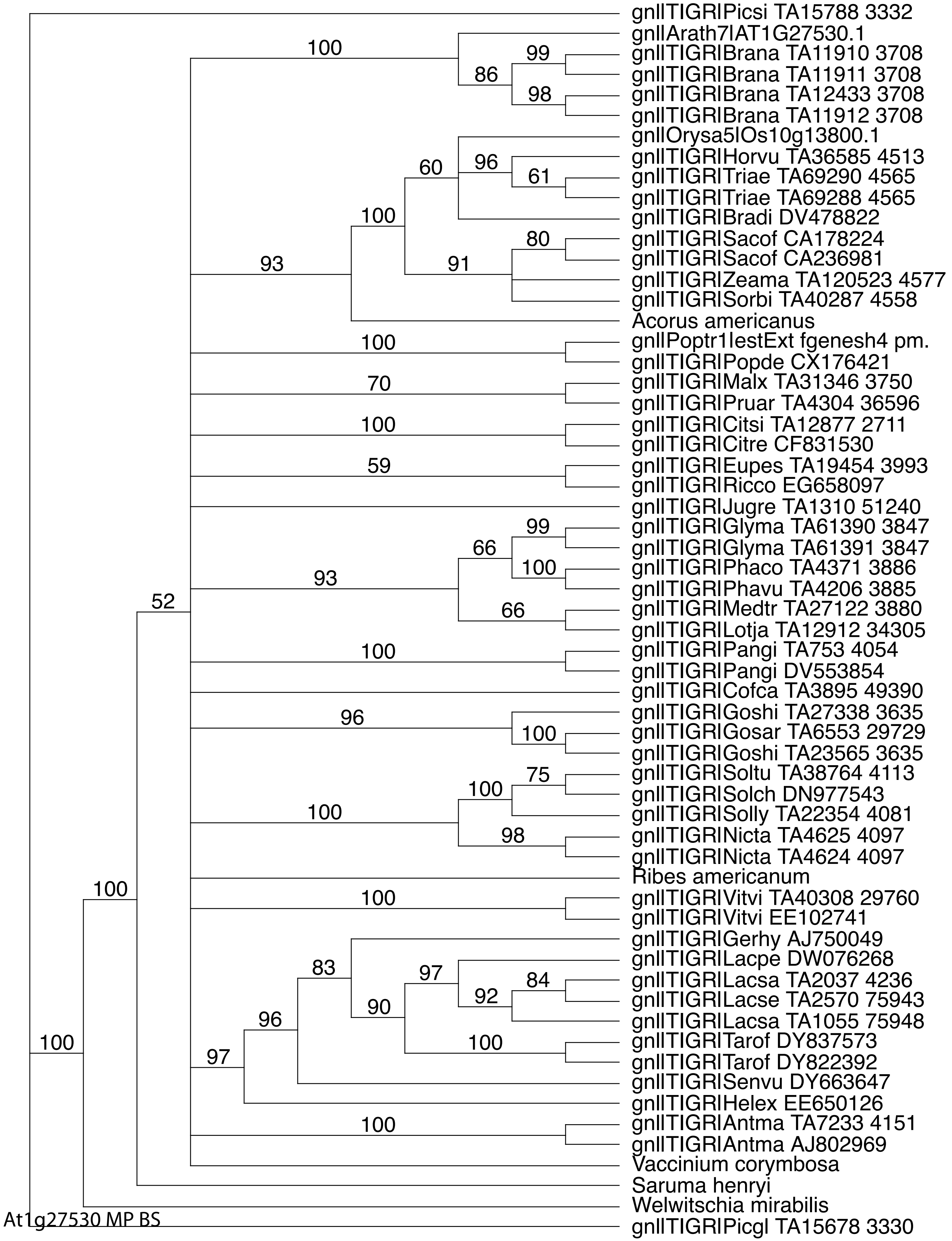

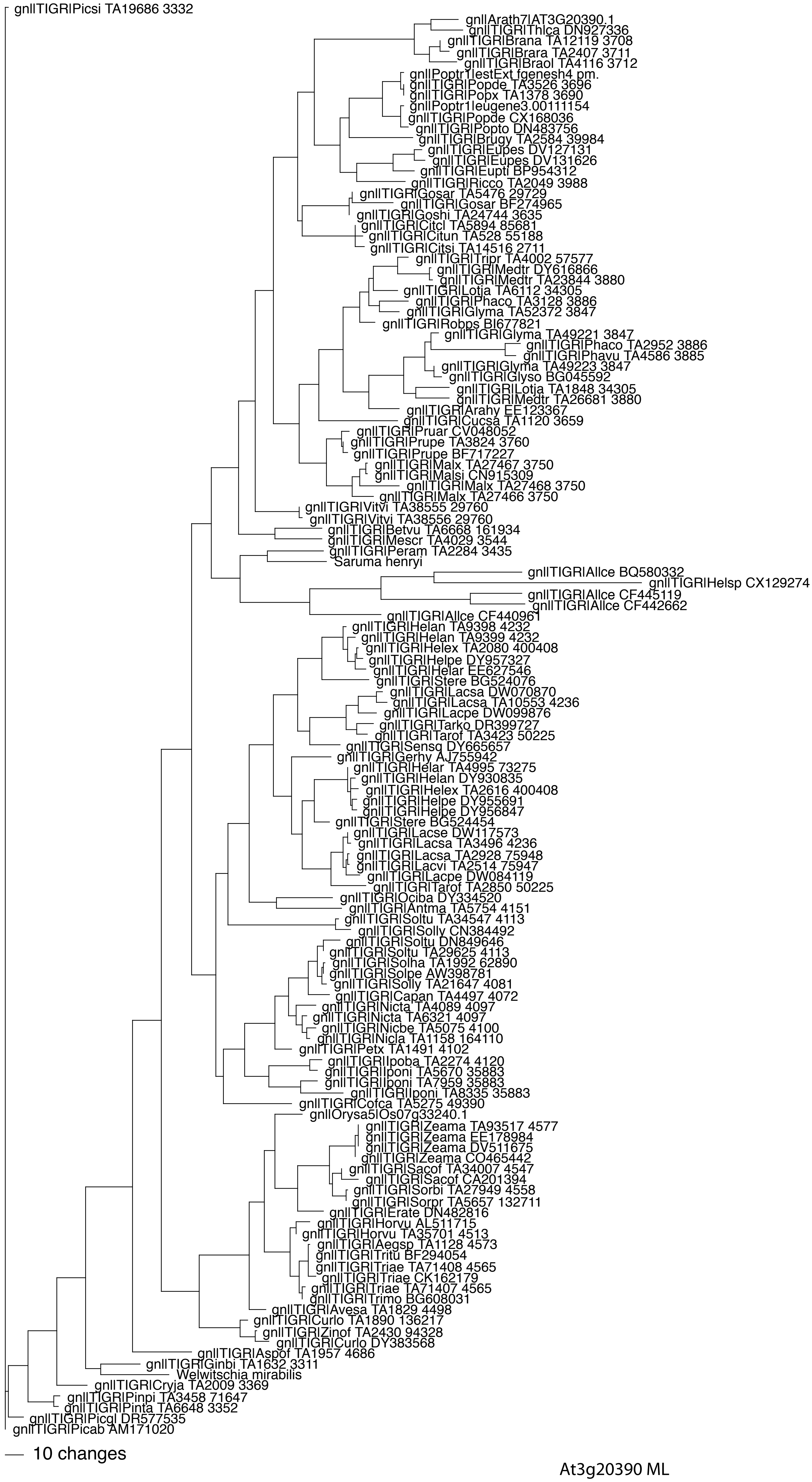

At3g20390 ML

Majority rule

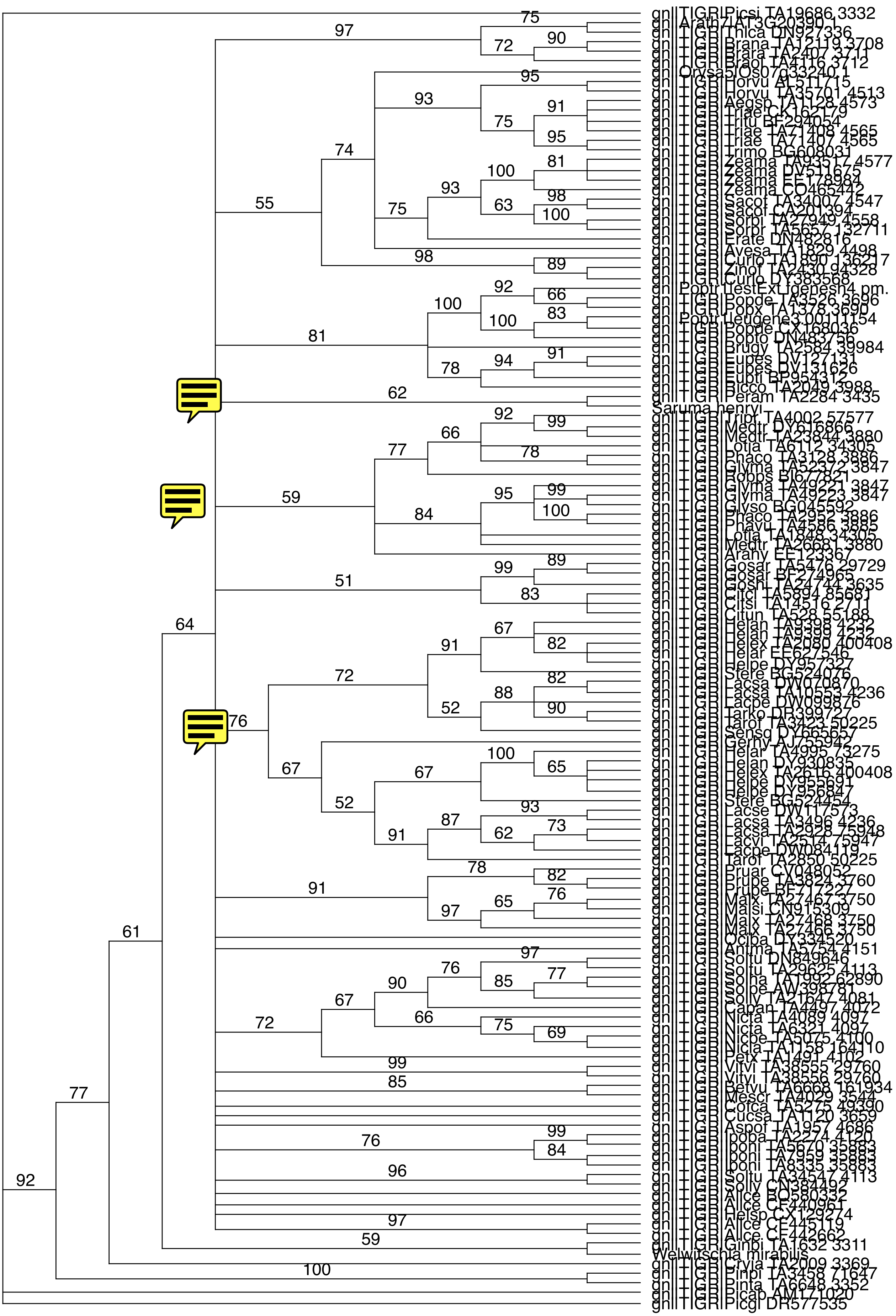

At3g20390 ML BS

Majority rule

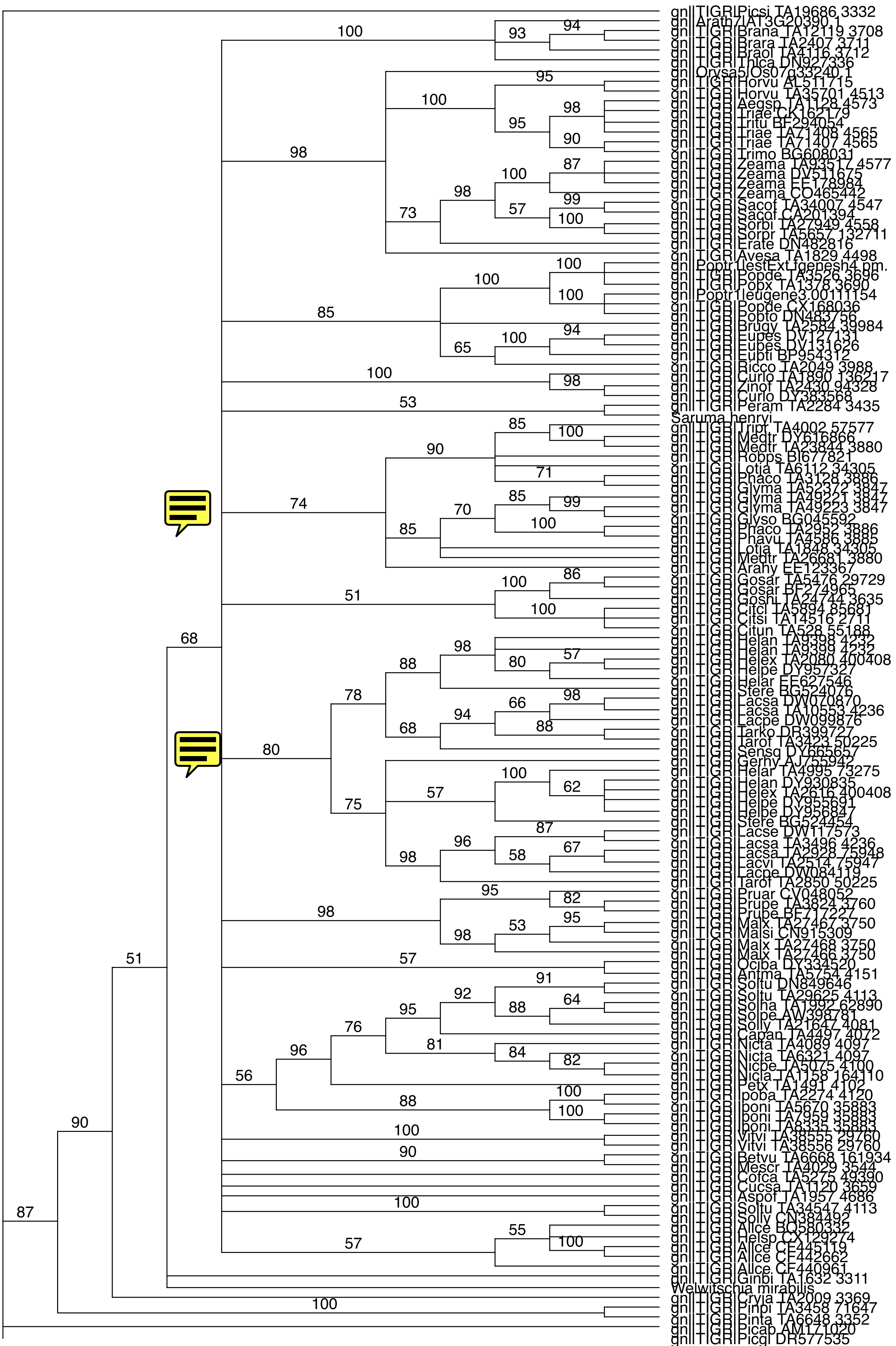

At3g20390 MP BS

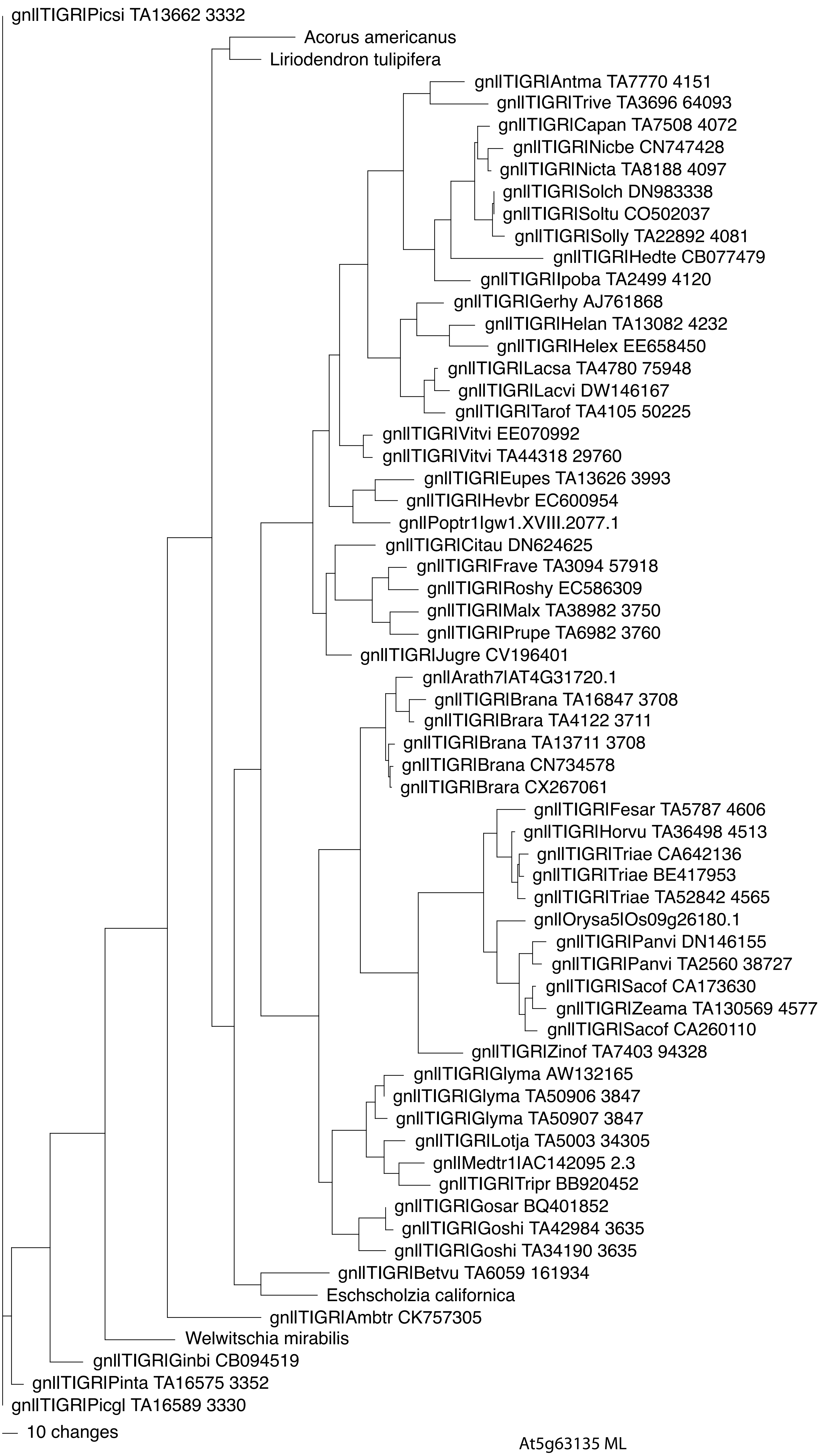

At5g63135 ML

Majority rule

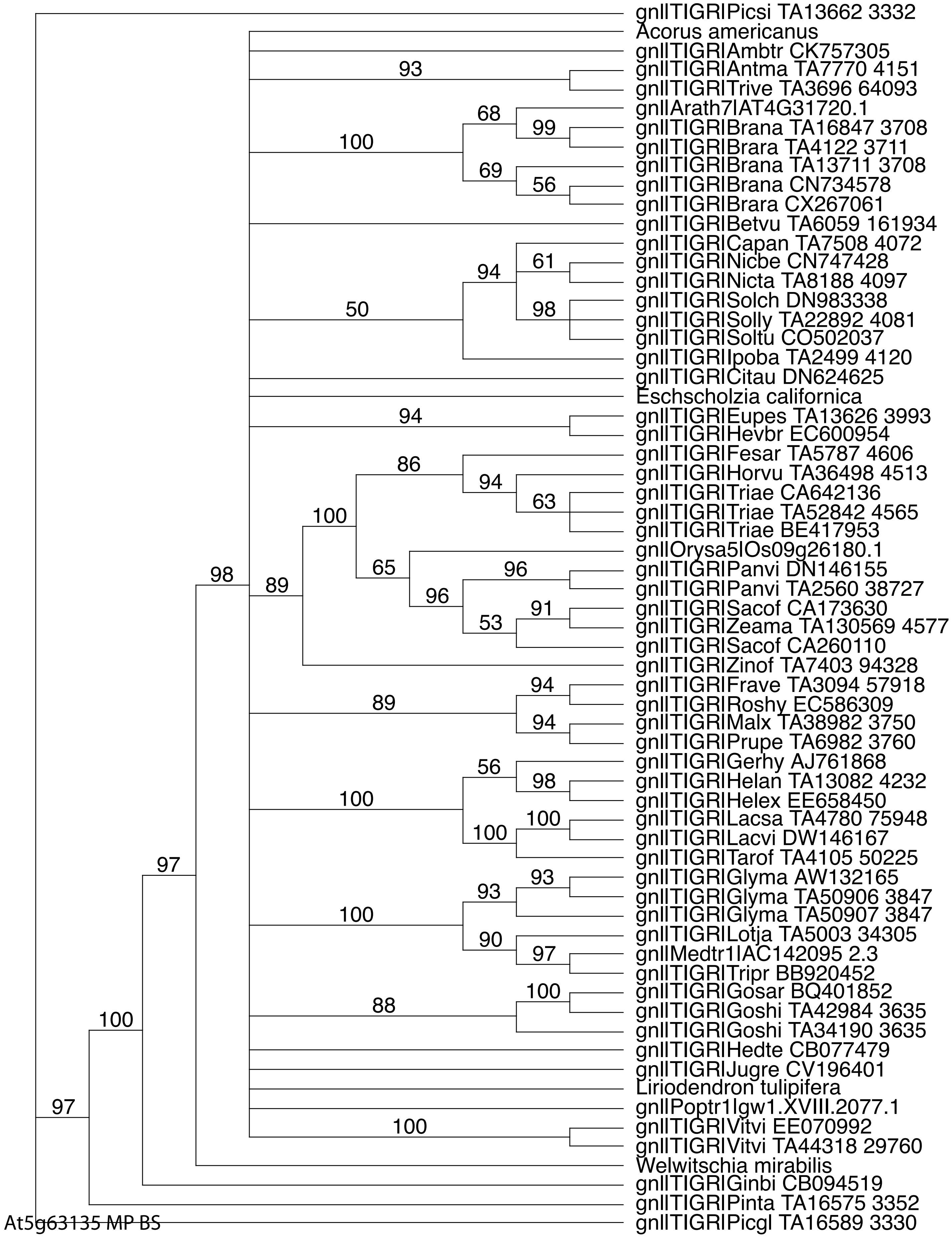

Majority rule

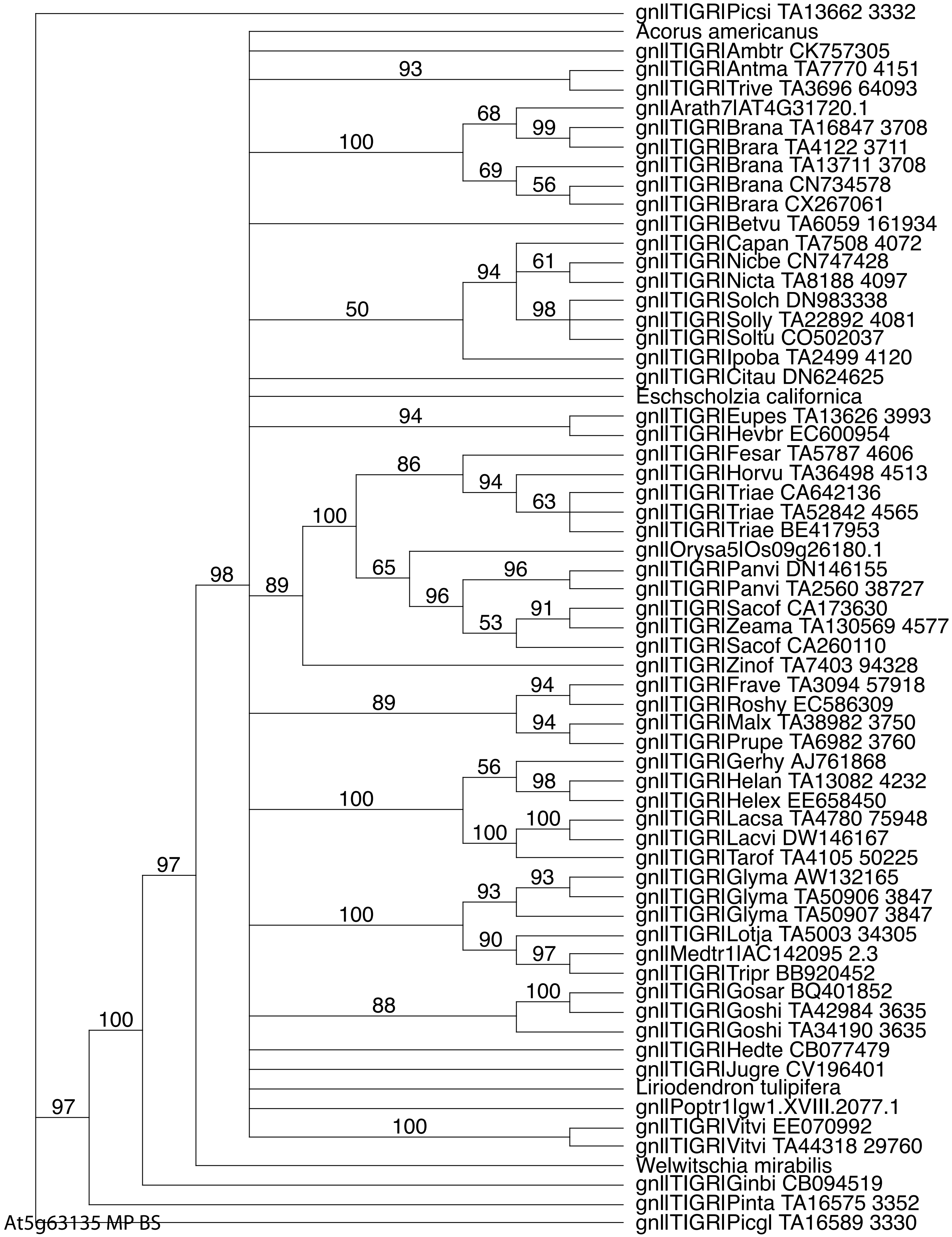

Majority rule

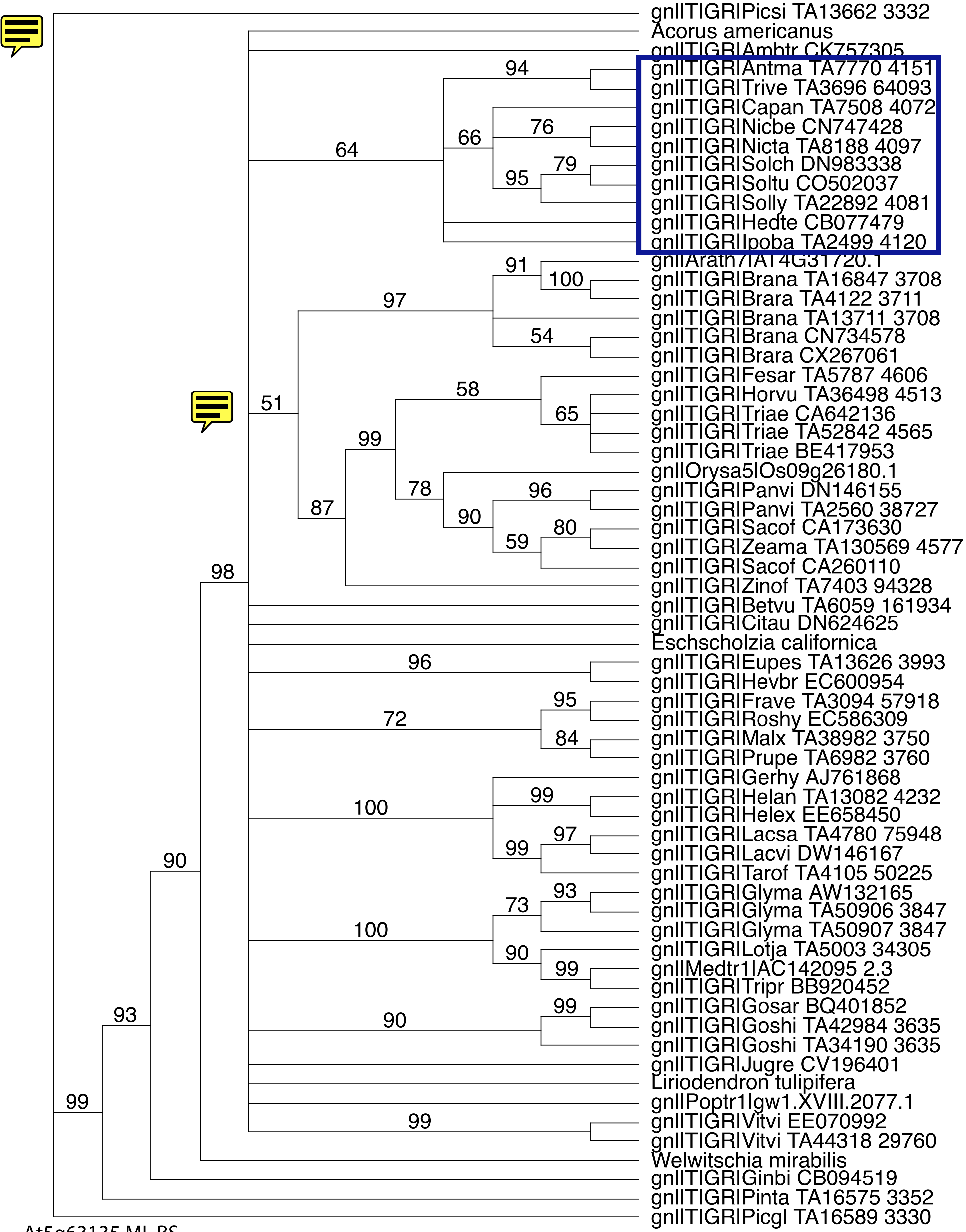

At5g63135 ML BS
